# A new genome scan for primary nonsyndromic vesicoureteric reflux emphasizes high genetic heterogeneity and shows linkage and association with various genes already implicated in urinary tract development

**DOI:** 10.1002/mgg3.22

**Published:** 2013-07-07

**Authors:** J M Darlow, M G Dobson, R Darlay, C M Molony, M Hunziker, A J Green, H J Cordell, P Puri, D E Barton

**Affiliations:** 1 National Centre for Medical Genetics, Our Lady's Children's HospitalCrumlin, Dublin, 12, Ireland; 2 National Children's Research Centre, Our Lady's Children's HospitalCrumlin, Dublin, 12, Ireland; 3 Institute of Genetic Medicine, Newcastle UniversityNewcastle upon Tyne, United Kingdom; 4 Merck & Co. Inc1 Merck Drive, Whitehouse Station, New Jersey, 08889; 5 National Children's HospitalTallaght, Dublin, 24, Ireland; 6 University College Dublin UCD School of Medicine and Medical Sciences, Our Lady's Children's HospitalCrumlin, Dublin, 12, Ireland

**Keywords:** CAKUT, genetic association, genetic linkage, ureteric bud, vesicoureteric reflux

## Abstract

Primary vesicoureteric reflux (VUR), the retrograde flow of urine from the bladder toward the kidneys, results from a developmental anomaly of the vesicoureteric valve mechanism, and is often associated with other urinary tract anomalies. It is the most common urological problem in children, with an estimated prevalence of 1–2%, and is a major cause of hypertension in childhood and of renal failure in childhood or adult life. We present the results of a genetic linkage and association scan using 900,000 markers. Our linkage results show a large number of suggestive linkage peaks, with different results in two groups of families, suggesting that VUR is even more genetically heterogeneous than previously imagined. The only marker achieving *P* < 0.02 for linkage in both groups of families is 270 kb from *EMX2*. In three sibships, we found recessive linkage to *KHDRBS3*, previously reported in a Somali family. In another family we discovered sex-reversal associated with VUR, implicating *PRKX*, for which there was weak support for dominant linkage in the overall data set. Several other candidate genes are suggested by our linkage or association results, and four of our linkage peaks are within copy-number variants recently found to be associated with renal hypodysplasia. Undoubtedly there are many genes related to VUR. Our study gives support to some loci suggested by earlier studies as well as suggesting new ones, and provides numerous indications for further investigations.

## Introduction

Primary vesicoureteric reflux (VUR), the retrograde flow of urine from the bladder toward the upper urinary tract, is the most common urological anomaly in children, and urinary tract infections (UTIs) and renal damage (known as reflux nephropathy) are common in VUR patients (Gargollo and Diamond 2007). Despite improvements in diagnosis and treatment of VUR, reflux nephropathy is still an important cause of childhood hypertension and chronic renal failure (Gargollo and Diamond 2007). Renal parenchymal damage can be congenital or acquired. Congenital reflux nephropathy occurs as a result of abnormal embryological development and is often seen in male infants with high-grade VUR (Patterson and Strife 2000). Exposure to UTIs in patients with congenital renal dysplasia can lead to progression of renal parenchymal damage (Gargollo and Diamond 2007).

The oft-quoted estimated prevalence of VUR is 1–2%, and appears to be derived from estimates that over 5% of children under 7 years have UTIs and that about 35% of children with UTIs have VUR (Scott et al. 1997). However, the prevalence of VUR may well be higher (Sargent 2000; Williams et al. 2008). The hereditary and familial nature of VUR is now well recognized, and numerous studies have shown that siblings of children with VUR have a much higher incidence of reflux than the general pediatric population (Chertin and Puri 2003). Diagnosis is made by micturating cystourethrogram (MCUG) to follow up findings either of appearance of hydronephrosis on pre- and postnatal ultrasound, or of recurrent UTIs, and screening of siblings of symptomatic children, or of children of affected parents, often reveals asymptomatic cases. Therefore, many cases must go undiagnosed, but the nature of the investigation is such that population screening is neither practical nor ethical. Furthermore, VUR can resolve spontaneously with age (Connolly et al. 1997; Chand et al. 2003; Menezes and Puri 2009). This not only frustrates efforts to estimate the incidence, but also frustrates efforts to investigate the genetics.

Prevalence rates of 27–51% in siblings of children with VUR and a 66% rate of VUR in offspring of parents with previously diagnosed reflux have been reported. The incidence of sibling VUR is maximal in patients who are younger than 3 years of age (Menezes and Puri 2009). Reflux in symptomatic siblings who are younger than 3 years of age is usually high grade and associated with a higher incidence of renal scarring (Menezes and Puri 2009). Because the prevalence of VUR is high in first degree relatives of VUR patients (Noe 1992; Noe et al. 1992; Kaefer et al. 2000; Hunziker and Puri 2012), most investigators believe that mutation of only one or two genes is required in most cases, rather than many, and that most mutations are dominant, though autosomal recessive inheritance has also been demonstrated (Ashraf et al. 2009) and X-linked inheritance has been proposed in some families (Middleton et al. 1975; Naseri et al. 2010). Mathematically, it cannot be ruled out that VUR inheritance is actually due to the combined effects of a large number of variants of small effect (Risch 1990), but the former belief is reinforced by results from mouse models, as well as the existence of human syndromes that include VUR and result from a single gene mutation (Puri et al. 2011).

Urinary tract development in the embryo begins with the formation of the ureteric bud, which is an outgrowth of the mesonephric (Wolffian) duct. Reciprocal signaling between the bud and the metanephric mesenchyme results in the growth of the ureteric bud to form the ureter and its branching to form the collecting ducts, and organization of the metanephric mesenchyme to form the kidney. Apoptosis occurs in the part of the mesonephric duct between the newly developed ureter and the urogenital sinus. The free end of the developing ureter inserts into the bladder wall and forms the vesicoureteric valve.

The ureterovesical junction (UVJ) acts as a valve and closes during micturation or when the bladder contracts. The UVJ is structurally and functionally adapted to allow the intermittent passage of urine and prevent the reflux of urine into the ureter. The main defect in patients with VUR is believed to involve the malformation of the UVJ, in part due to shortening of the submucosal ureteric segment due to congenital lateral ectopia of the ureteric orifice. The precise position at which the ureteric bud grows out from the mesonephric duct is critical not only to the position and angle at which the ureter is inserted into the bladder, but to the degree of renal dysplasia (due to the ureter growing into mesenchyme that is less able to form kidney) and if the budding is bifid, a duplex kidney will form (Mackie and Stephens 1975). Work with mouse embryos has revealed many of the genes involved in the precise control of ureteric budding and subsequent urinary tract and kidney development (Ichikawa et al. 2002; Murawski and Gupta 2006; Murer et al. 2007; Schedl 2007; Chen 2009; Uetani and Bouchard 2009; Song and Yosypiv 2011). Primary VUR could be due to mutations in one or more developmental genes that control these processes.

The term CAKUT was coined (Ichikawa et al. 2002) to emphasize the fact that Congenital Anomalies of the Kidney and Urinary Tract commonly occur together, and in both Mouse and Man they are commonly seen in the same individuals or the same sibships in which VUR occurs; embryological work in mice has shown that many of the same genes that are involved in initiating ureteric bud development are also involved in kidney development. It should be noted, though, that there are also many other genes that come into action at the later stages (Schedl 2007; Brunskill et al. 2011; Nishinakamura et al. 2011; Potter et al. 2011), and mutation of these genes can cause renal dysplasia without VUR. There are also various syndromes that include VUR and CAKUT along with anomalies of other organs, and for these a number of the genes have been identified (see reviews [Murer et al. 2007; Puri et al. 2011]). However, for nonsyndromic VUR, mutations accounting for only a small proportion of cases have been found, and most of these were in patients with CAKUT (*PAX2* [Nishimoto et al. 2001], *UPK3A* [Jenkins et al. 2005; Schonfelder et al. 2006], *UPK2* [Jenkins et al. 2006], *HNF1B*(*TCF2*) [Weber et al. 2006], *ROBO2* [Lu et al. 2007; Bertoli-Avella et al. 2008], *SIX2* [Weber et al. 2008], *BMP4* [Weber et al. 2008], *SOX17* [Gimelli et al. 2010] and *TNXB* [Gbadegesin et al. 2013]). Studies to search for genes involved in primary nonsyndromic VUR and/or CAKUT have included association, linkage, and exon-sequencing studies of candidate genes ([Jenkins et al. 2007; Saisawat et al. 2012; van Eerde et al. 2012] and references within reviews [Murawski and Gupta 2006; Song and Yosypiv 2011]), genome-wide linkage and association studies (Feather et al. 2000; Kelly et al. 2007; Sanna-Cherchi et al. 2007; Conte et al. 2008; Weng et al. 2009; Briggs et al. 2010; Cordell et al. 2010; Marchini et al. 2012), array-based comparative genomic hybridization (Weber et al. 2011), and gene expression studies (McMahon et al. 2008).

Candidate gene studies have had little success, and one likely reason for this, which comes out of the developmental genetic studies in other species, is that most of the genes so far known to be involved in urinary tract development are also involved in the development of other organs. It is logical, therefore, to expect that mutations that alter a protein's sequence are likely to affect all the organs in whose development it is involved, causing a syndrome of anomalies. Thus, of those genes listed above in which a few mutations have been found that cause nonsyndromic VUR or VUR and CAKUT, *PAX2* is also involved in eye development, and most mutations cause Renal Coloboma Syndrome (Bower et al. 2012), *HNF1B* is also involved in liver, pancreas, and genital development, and nephropathy patients with *HNF1B* mutations frequently have extrarenal phenotypes (Faguer et al. 2011), *ROBO2* is involved in axon guidance (Zhang et al. 2012) and cardiovascular development (Mommersteeg et al. 2013), and there is some doubt about whether heterozygous nonsynonymous variants can actually cause VUR on their own (Dobson et al. 2013), and *BMP4* mutations can result in eye defects, pituitary defects, brain malformations, and digital anomalies (Slavotinek 2011). Indeed, there are genes known to be involved in urinary tract development in which no mutations have been found that cause isolated VUR. For instance, mutations in *RET* can cause Hirschsprung's Disease (Wallace and Anderson 2011), Multiple Endocrine Neoplasia Type 2 (Pasquali et al. 2012), and isolated medullary carcinoma of thyroid (Zhou et al. 2012); one nonsynonymous variant was thought to cause VUR (Yang et al. 2008), but this was shown not to be so (Darlow et al. 2009).

However, developmental genes have many different noncoding regulatory elements, which regulate their expression in different tissues, and study of the effects of chromosomal rearrangements shows that these can be at distances of a megabase or more on either side of the gene, and may even be inside neighboring genes (Kleinjan and van Heyningen 2005). It therefore seems reasonable to expect that, though there may be some genes in which all mutations cause only VUR, and others in which some particular mutations may cause VUR without any phenotypic features relating to the other actions of those genes, many of the mutations that cause VUR and other developmental disorders of the urinary tract alone, not involving other organs, may be in noncoding DNA, and could be at some distance from the genes that they affect.

The genome scans quoted above have all had different results, posing the question of whether mutations at different loci have different relative frequencies in different populations. Since our first genome scan for VUR (Kelly et al. 2007), we have recruited almost as many more families from the same population. In this new study, we not only use more families, but more single-nucleotide polymorphism (SNP) markers, and we include the genotypes of healthy controls from the same population. These additions allow us not only to cover the genome in more detail, but to examine replicability of linkage results, by comparing two sets of families from the same population, and to test for genetic association.

## Materials and Methods

### Patients and families

The samples for this study were collected at Our Lady's Children's Hospital Crumlin and the National Children's Hospital, Tallaght, both in Dublin, Ireland. Ethical approval was granted by the ethics committees of both hospitals, and informed consent was obtained from all subjects and/or their parents. Families with two or more affected members with primary VUR of any grade were collected. All families are Caucasian and the majority considered to be of homogeneous Irish ancestry. Most index cases were referred because of recurrent UTIs and all were diagnosed by MCUG. Sibs of index cases were screened by MCUG. Fifteen parents and one grandparent of affected children were classed as affected because they had been diagnosed with primary VUR in the past.

Because of sample drop-out, affection status and family structure, not all samples could be used for all analyses, but there were 900 samples from 225 families (nine extended and 216 nuclear) that could be used for at least one of the three analyses, linkage, transmission disequilibrium test (TDT), and case–control association, of which 500 were from VUR patients (201 male and 299 female). Seventy-one patients had at least one additional urinogenital tract anomaly, 40 of them having at least one duplex kidney. Numbers of samples used for each analysis are given in sections below.

### Irish population controls

A DNA sample collection from peripheral blood samples from healthy members of the Irish population was established as the Irish Blood Transfusion Service – Trinity College Dublin (IBTS-TCD) BioBank. These samples had already been genotyped using the Affymetrix (Carlsblad, CA) Genome-Wide Human SNP Array 6.0 before our patients and families were genotyped (Purcell et al. 2009), and the CEL files were made available to us in order to determine control genotypes for this study.

### Genotyping and quality control

DNA samples were checked for quality by spectrophotometry (on a Nanodrop ND-1000, NanoDrop products, Wilmington, DE) and agarose gel electrophoresis. A few samples that were of low concentration or partially degraded (wider fragment size spread than highest quality genomic DNA) were sent for whole-genome amplification by Qiagen REPLI-g Services (Qiagen GmbH, Hilden, Germany). All samples were then diluted to a standard concentration, plated, and sent to Atlas Biolabs GmbH (Berlin, Germany) who rechecked DNA quality by gel analysis and then genotyped the samples on the Affymetrix Genome-Wide Human SNP Array 6.0.

Genotypes were “called” (determined from the raw fluorescence data) from the Affymetrix CEL files using Affymetrix Genotyping Console (version 4.1.1.834). CEL files with a contrast QC below 0.4 were removed from analysis, and those remaining were called using the Birdseed V2 algorithm and a two-step workflow. The initial round of genotype calling (which was used to remove poor samples prior to the second genotyping round) was performed using the Birdseed V2 algorithm with VUR and BioBank samples in a single batch; samples with a call rate below 95% or an autosomal SNP heterozygosity rate more than 3 SD from the mean were removed from further analysis. The second round of genotype calling was performed with VUR and BioBank samples in separate batches; no further removal of CEL files was necessary. Genotype data was generated at 834,482 SNPs across the genome. Markers that produced a genotype in at least 95% of the batch of CEL files were exported to PLINK format for use in further analysis.

The next stage of quality control was performed using PLINK (Purcell et al. 2007) version 1.07 with visualization performed in R (http://www.r-project.org). Samples with genotype call rates below 97% and average heterozygosities outside the range 0.30–0.32 (based on consideration of 831,367 autosomal SNPs with a Hardy–Weinberg equilibrium test *P-*value greater than 10^−8^) were excluded.

A set of 35,919 autosomal SNPs, that were successfully genotyped in at least 95% of individuals, had a Hardy–Weinberg equilibrium test *P-*value >10^−8^, minor allele frequencies greater than 0.4 and that had been pruned to show low levels of linkage disequilibrium (using the PLINK command “–indep 50 5 2”) were created and used to check relationships, sample duplications, and ethnicities. Genome-wide identity-by-descent (IBD) sharing was calculated (using the “–Z-genome” command in PLINK). All unexpected findings were checked by microsatellite analysis of the DNA using the PowerPlex® 16 System (Promega, Madison, WI) followed by rechecking patient information where necessary. Sample identities/plate maps were then corrected, and the genotype sets subjected to a further round of quality control. This revealed that several pairs of nuclear families, separately ascertained and recruited into the study, were related. These relationships were checked by contacting the families, and the relationships coded into the sample information. Multidimensional scaling of the samples together with 210 unrelated Phase II HapMap (Frazer et al. 2007) individuals from four populations (CEU, JPT, CHB, YRI) (using the same set of 35,919 autosomal SNPs) was performed and identified a family of four individuals that did not cluster with the CEU samples, suggesting non-European ancestry; these individuals were excluded. The “–check-sex” option in PLINK was used to check that the gender of our samples matched its expected value.

Within each of the VUR and BioBank cohorts, any SNPs with minor allele frequencies less than 0.01, that were successfully genotyped in less than 95% of individuals or that had a Hardy–Weinberg equilibrium test *P-*value less than 10^−8^ were excluded. Within the VUR cohort, SNPs showing greater than 10% Mendelian inheritance errors were excluded. For analyses involving both cohorts, A/T and G/C SNPs were removed to avoid possible strand flips. These exclusions resulted in a final set of 643,691 autosomal and X-chromosomal SNPs used for analyses performed within the VUR cohort, and 582,923 autosomal and X-chromosomal SNPs used for analyses involving the combined VUR and the BioBank cohorts.

### Linkage analysis

SNPs that passed quality control were used to perform multipoint parametric (model-based) and nonparametric (model-free) linkage analysis across the genome. A total of 199 families (with 467 affected individuals, 192 male, 275 female) were used in the linkage analysis, of which 118 (with 281 affected individuals, 116 male, 165 female) had been used in our previous genome scan (Kelly et al. 2007) and are referred to as “the old families,” and 81 (with 186 affected individuals, 76 male, 110 female) are new in this genome scan. For further details see Supp. Table S1. Six of the extended families were counted in the old group (several with new members added) and three in the new group. For reasons of computational efficiency, and to avoid using SNPs that are in linkage disequilibrium with one another (which can cause false-positive inflation of linkage evidence), the SNPs were first pruned to include only those with minor allele frequencies greater than 0.4 and low levels of linkage disequilibrium (using the PLINK command “–indep 50 5 2”) and then thinned to use the two SNPs with the highest heterozygosity in each 1-cM window, using the program MapThin (Howley and Cordell) at web address http://www.staff.ncl.ac.uk/richard.howey/mapthin/. Examination of the resulting information content plots (see Figs. S1 and S2) indicated that this thinned set of SNPs (*n* = 7051) provided adequate linkage information. The data set was then “wiped” by processing with the MERLIN (Abecasis et al. 2002) utility program PedWipe, which tests for and removes unlikely genotypes from the data. The program MERLIN was then used to calculate information content and test for linkage using a multipoint “equivalent LOD score” corresponding to the Kong and Cox exponential model likelihood-based allele-sharing test (Kong and Cox 1997), which we denote here as “ZLRLOD.” MERLIN was also used to perform parametric linkage analysis allowing for heterogeneity (an “HLOD” analysis), assuming a disease allele frequency of 0.01, under both recessive (with penetrances of 0.01, 0.01, and 0.99) and dominant (penetrances 0.01, 0.99, and 0.99) models (Abreu et al. 2002). (These calculations are not expected to be highly sensitive to the exact allele frequency chosen, so it was not a concern that frequency used probably did not match the unknown real underlying disease allele frequency.) The X chromosome was analyzed with the program MINX under the same parameters as used in MERLIN.

### Association analysis

PLINK was used to perform a TDT (Spielman et al. 1993) at the 643,691 SNPs passing our QC threshold on 410 parent-affected child trios from 186 families; of the 410 children, there were 170 boys and 240 girls. Visual assessment of significance was carried out through examination of Q–Q plots, which is broadly equivalent to use of a Bonferroni correction to assess the overall significance of a given result in light of the multiple tests performed. A likelihood-based association test similar to the TDT was also performed using UNPHASED (Dudbridge 2008) in order to ascertain the genotype relative risk conferred by each SNP.

Case–control analysis was performed at 582,923 SNPs passing QC in 500 VUR cases and 851 BioBank controls. Data was initially analyzed in the R package GenABEL (Aulchenko et al. 2007). In order to correct for the pedigree structure within the cases, an estimated kinship matrix was first computed, based on the genome-wide SNP data, and this was then passed to the “polygenic” function in order to estimate residuals of the trait for further use in analysis with the function “mmscore” (Chen and Abecasis 2007). This function performs a score test for association between a trait and genetic polymorphism, in samples of related individuals.

In addition to GenABEL, the programs ROADTRIPS (Thornton and McPeek 2010), EMMAX (Kang et al. 2010), and FaST-LMM (Lippert et al. 2011) were also used as alternative methods for correcting for sample structure within the autosomal data. EMMAX (efficient mixed-model association expedited) and FaST-LMM (factored spectrally transformed linear mixed models) are variance component approaches similar to the approach implemented via mmscore in GenABEL. The utility “emmax-kin” under the default BN argument was used to calculate kinship coefficients for passing to EMMAX, while the FaST-LMM algorithm was carried out using the default RRM (realized relationship matrix) argument to account for genetic similarity. For ROADTRIPS, the “KinInbCoef” utility was first used to obtain known (theoretical) kinship coefficients, which are then used by ROADTRIPS (together with the empirical covariance matrix estimated from genome-wide genotype data) to correct for known and unknown relatedness and population structure.

Following association analysis, any SNPs showing significant association with disease status were checked via visual inspection of the SNP intensities from the fluorescence data (“cluster plots”) and were also checked for consistency of allele frequencies and LD patterns in the region with the known patterns from CEU HapMap data. SNPs deemed to be unreliable on the basis of these checks were removed.

## Results

### Linkage analysis

In our previous genome scan (Kelly et al. 2007), we carried out analyses on a set of 129 families and on the subset remaining after removing 25 families in which one or more of the affected children (usually only one of them) had some additional anomaly of genitourinary tract development beyond isolated VUR, a duplex kidney in most cases. Comparison of the genome-wide linkage results for the two sets showed only small differences that could easily be explained by chance variation in the proportions of families with linkage to different loci, so we concluded that isolated VUR and VUR with additional urinary tract anomalies were not genetically distinct conditions, and this time we did not separate families in this way. However, we did separate the results from this new scan with new markers into those from families involved in the original scan (the “old” families) and those not previously genotyped (the “new” families), as well as combining them. We have also collected much information on family histories of VUR or other urinary tract problems, and we carried out “strict” analyses, in which only individuals with radiographically confirmed VUR or duplex kidney were considered affected, and “loose” analyses, in which parents with a history of VUR or kidney problems on their side of the family were counted as affected, on the bases that they were probably the parent transmitting VUR to the children and that VUR is usually dominant. The results showed that at least as often as not, the loose analyses did not achieve higher linkage scores (in addition to being harder to justify theoretically), and therefore only the results of strict analyses are reported. However, it is notable that of the 209 recruited nuclear families useful for linkage (including those that were parts of collected extended families), 111 had family history of VUR or kidney problems and/or an affected parent, some on both sides.

The “old families” group in this study is not precisely the same as in our first genome scan because (a) some new family members have been added, (b) some samples used last time were not plated, or failed quality control, and (c) some families independently ascertained have been found to be related, and so this time have been recognized as extended families. Also, this time a different set of markers has been used, and precise details of the parameter settings used may also be different. Thus, the results from the old families presented here should not be expected to be exactly the same as previously published, though there is a good correspondence.

**Figure 1:**
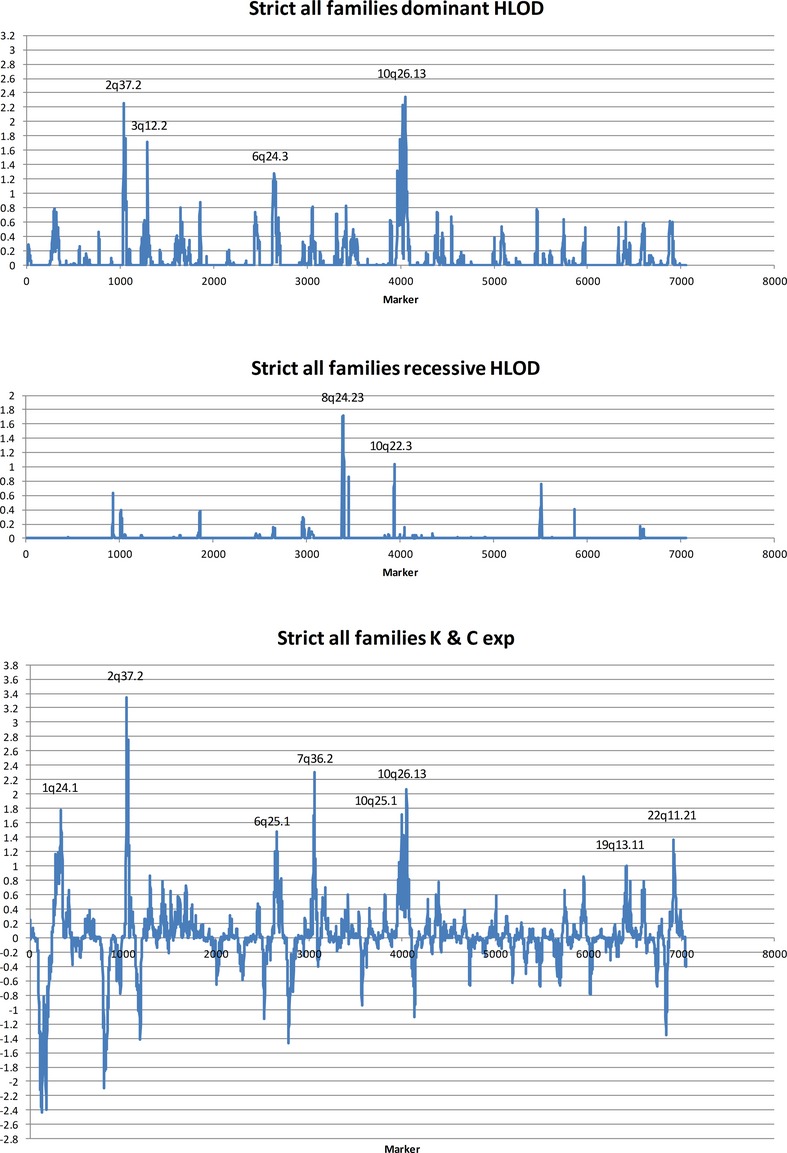
Plots of genetic linkage across the autosomes calculated as heterogeneity logarithm of odds (HLOD) on a dominant model, HLOD on a recessive model, and nonparametric linkage by the Kong and Cox exponential method.

**Figure 2:**
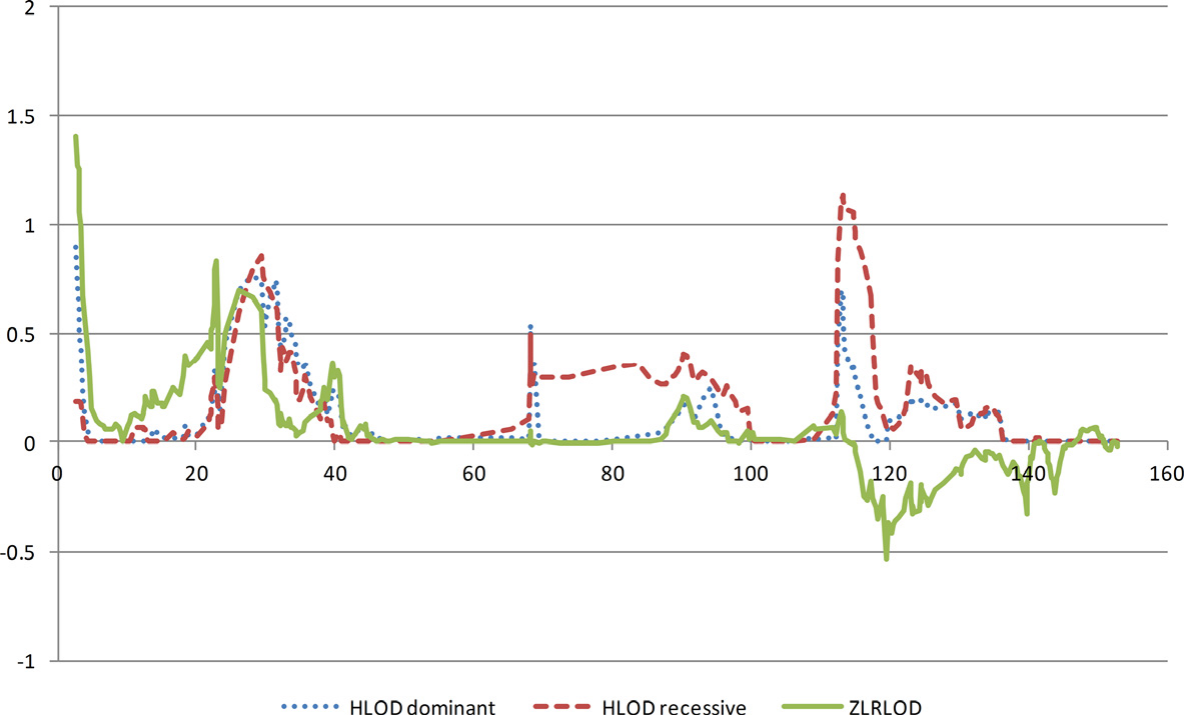
Linkage for all families on the X chromosome. *AGTR2* is within the recessive linkage peak but the highest marker is 2 Mb proximal to the gene.

Figures 1 and 2 show (for the full set of families) the results from three different methods of linkage analysis across the autosomes and the X chromosome, respectively: the heterogeneity logarithm of odds (HLOD) using a dominant and a recessive model of inheritance, and the equivalent LOD (ZLRLOD) from the nonparametric linkage (Kong & Cox exponential model) method (Kong and Cox 1997). The results confirm the previous findings that VUR is heterogeneous with suggestive linkage to numerous genomic loci. It is also clear that more loci fit a dominant model of inheritance than a recessive one, but that greater evidence of linkage is detected at more loci using a nonparametric approach. We noticed that the linkage pattern was not quite the same as we obtained in the first scan, so also examined the results for the (118) original (“old”) and (81) new families separately. We compared the dominant HLOD analyses of the “old” and “new” groups of families against the result for all families combined (Fig. S3) and made the same comparisons for the recessive HLOD analyses and the nonparametric linkage analyses (Figs. S4 and S5). These comparisons show very little similarity in the linkage patterns between the two groups of families recruited from the same population. One explanation of these results would be that the apparent linkage peaks are artifacts of chance due to the small sample sizes, but since evidence of genetic etiology of VUR and CAKUT is overwhelming, another interpretation is that VUR is even more heterogeneous than we had previously imagined, so that only small numbers of families in our sample are related to any one locus, and some loci will only be represented in one group or the other.

**Figure 3:**
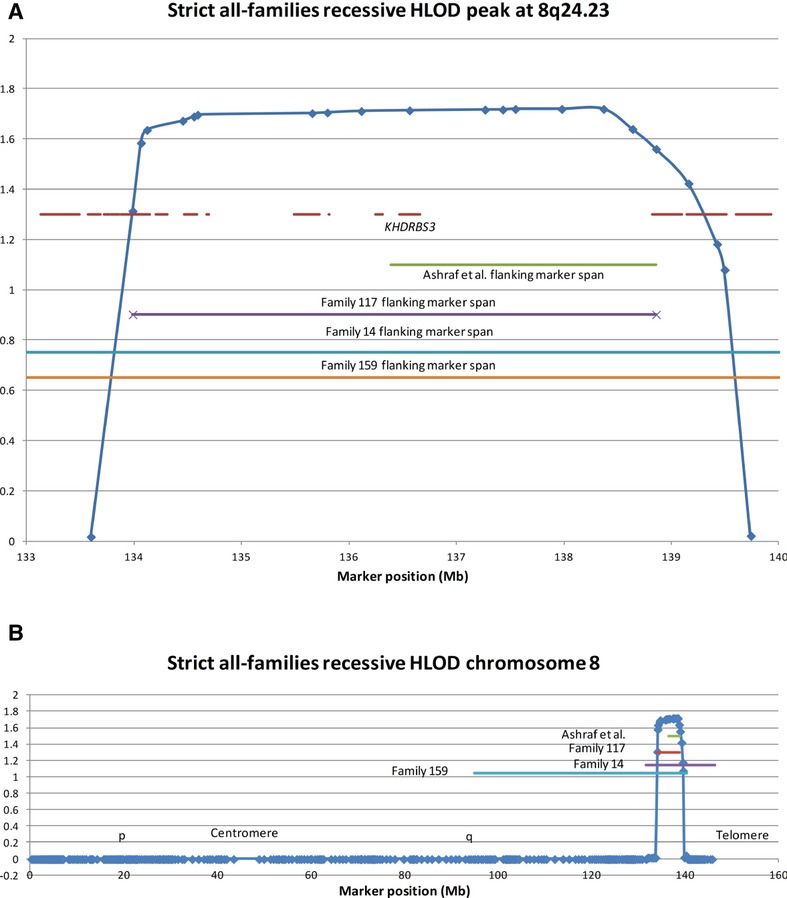
The recessive linkage peak on 8q (A) in detail – the dashes are genes; (B) in the context of the whole chromosome to show the lengths of haplotype sharing by siblings in the contributing families and in the previously published family of Ashraf et al. 2009.

Support for this interpretation is given by scrutiny of the recessive linkage peak at 8q24.23. This peak appears in the old but not the new families analysis, and the maximum HLOD score from the combination of all families is only 1.72, but we were interested in it because it corresponds to the position of recessive linkage reported by Ashraf et al. (2009). Inspection of the per-family recessive linkage to 8q24.23 revealed that there are just three families whose HLOD scores are >0.01. These are families 14, 117, and 159, which are all in the “old” group. Ashraf et al. (2009) investigated a single Somali family with 12 children, eight of whom had various manifestations of CAKUT including high-grade VUR, and found that there was a single region of the genome in which all of the affected children shared the same haplotype from their father and the same haplotype from their mother. This shared region was only 2.5 Mb and contained a single gene, *KHDRBS3*. This gene is in the very center of our own linkage peak (Fig. 3A). Family 117 has six affected children, five of whom were genotyped for this study, and achieves the highest per-family LOD score (2.24) and has the smallest genomic region common to all siblings (5 Mb), while each of the other families has three affected children and naturally they achieve lower LOD scores and have wider regions of overlap between siblings (Fig. 3B). Importantly, though, they all overlap the region of the Somali family completely. We also note that, in keeping with CAKUT in the Somali family, in Family 14, one of the children has bilateral hydrocele, and in each of the other two families, one of the children has a duplex kidney. Ashraf et al. (2009) sequenced all the exons of *KHDRBS3* and did not find the mutation causing VUR in their family. They did not examine the promoter, so we examined 1000 bp upstream and the 5′ untranslated region in our three linked families as a first easy step in searching noncoding DNA. We found no new variants that segregated with VUR either. There is at least another 2.5 Mb waiting to be examined. None of the families gave a history of consanguinity, and examination of the genotypes of the SNPs in the region confirmed this, so we expect to find between two and six different recessive mutations between the three families. We should also add that in all three of our families all of the children were affected. However, this is probably ascertainment bias rather than indicating preferential transmission of the pathogenic allele, because families are only recruited if at least two children are affected, and only three families with only two children affected each would be below the level of detection as a linkage peak. Indeed the peak we did detect would probably have been ignored if it had not coincided with another study.

Abreu et al. (2002) suggest that an HLOD of approximately 1.2 for a fully penetrant autosomal dominant genetic model corresponds to a *P*-value of 0.01 and genome-wide results exceeding this level are reported in Table 1 for exploratory purposes, as this model could be considered a rough approximation of a genetic model of VUR. ZLRLOD results with *P-*value <0.02 are shown. With these thresholds, inevitably some of the results reported will be false positives, but raising the thresholds is likely to remove some genuine linkages achieving low significance due to the small numbers of families. Supplementary figures show superimposed linkage plots of nonparametric and HLOD dominant and recessive analyses for individual autosomes for all families (Fig. S6) and for the old and new groups of families (Figs. S7 and S8).

**Table 1:** All linkage results of HLOD >1.2 or ZLRLOD *P* < 0.02

	All families			Old families			New families		
Chr pos	Marker	Score	Analysis	Marker	Score	Analysis	Marker	Score	Analysis
1q24.1	rs2281962	1.782, *P* = 0.002086	ZLRLOD						
1q24.3				rs12401697	1.534, *P* = 0.00393	ZLRLOD			
2p25.1							rs7569485	1.469, *P* = 0.004642	ZLRLOD
2q37.2	rs1881187	3.341, *P* = 4.38 × 10^−5^	ZLRLOD	rs1881187	2.975, *P* = 0.00011	ZLRLOD			
	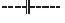	2.2539, α = 0.2655	HLOD dom						
2q37.3				rs6543544	1.7681, α = 0.3139	HLOD dom			
3q12.2	rs7429915	1.7189, α = 0.1783	HLOD dom	rs6803634	1.4233, α = 0.1958	HLOD dom			
3q27.3							rs10513807	1.295, *P* = 0.0073	ZLRLOD
4p16.3							rs3762867	1.247, *P* = 0.00829	ZLRLOD
4q22.3							rs11729799	1.7548, α = 0.3965	HLOD dom
							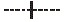	1.944, *P* = 0.00139	ZLRLOD
4q25							rs3866823	2.8711, α = 0.4747	HLOD dom
							rs17042944	2.304, *P* = 0.00056	ZLRLOD
4q34.2							rs17063114	1.9684, α = 0.2731	HLOD dom
							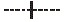	1.396, *P* = 0.00561	ZLRLOD
4q34.3							rs2378811	1.3471, α = 1	HLOD rec
5q14.1							rs6867021	1.164, *P* = 0.0103	ZLRLOD
6q24.3	rs12199542	1.1775, α = 0.1946	HLOD dom						
6q25.1	rs2256135	1.478, *P* = 0.00454	ZLRLOD						
6q25.2				rs9478613	2.1001, α = 0.3201	HLOD dom			
6q27				rs6924357	1.471, *P* = 0.004628	ZLRLOD			
7q36.2	rs6973441	2.306, *P* = 0.000559	ZLRLOD	rs2533241	2.922, *P* = 0.000122	ZLRLOD			
				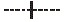	1.3149, α = 0.2768	HLOD dom			
8q24.23	rs11776993-	1.6894, α = 0.4702	HLOD rec	rs11776993-	1.6861, α = 0.4922	HLOD rec			
	rs4397435	1.7192, α = 0.4785		rs4397435	1.7232, α = 0.4985				
10q21.3							rs2244205	1.5084, α = 0.2385	HLOD dom
10q25.1	rs1245911	1.7553, α = 0.2346	HLOD dom	rs1411291	1.6984, α = 0.2853	HLOD dom			
	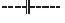	1.72, *P* = 0.002443	ZLRLOD	rs1372189	1.079, *P* = 0.01291	ZLRLOD			
10q26.11	rs10886146	2.2317, α = 0.2623	HLOD dom	rs10886146	1.6067, α = 0.2627	HLOD dom			
	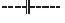	1.423, *P* = 0.005232	ZLRLOD				rs10886146	1.034, *P* = 0.01456	ZLRLOD
10q26.13	rs4962418	2.3394, α = 0.2868	HLOD dom						
	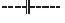	2.064, *P* = 0.001024	ZLRLOD						
10q26.3				rs4751013	1.7937, α = 0.3447	HLOD dom			
				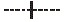	2.183, *P* = 0.007608	ZLRLOD			
11q25							rs497747	1.03, *P* = 0.01472	ZLRLOD
12p13.31							rs12582976	1.7686, α = 0.369	HLOD dom
12p13.2							rs7137455	1.122, *P* = 0.01149	ZLRLOD
13q33.2				rs9514424	1.236, *P* = 0.008512	ZLRLOD			
15q26.2				rs6416595	1.2433, α = 0.1198	HLOD dom			
16q24.1				rs7197843	1.213, *P* = 0.009058	ZLRLOD			
				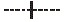	1.1806, α = 0.2435	HLOD dom			
19q13.11	rs529579	1.006, *P* = 0.01569	ZLRLOD						
19q13.33				rs352822	1.301, *P* = 0.007184	ZLRLOD			
20p12.1				rs2876409	2.167, *P* = 0.000792	ZLRLOD			
				rs6110544	1.7246, α = 0.3133	HLOD dom			
21q22.3				rs8129605	1.252, *P* = 0.008167	ZLRLOD			
22q11.21	rs5746685	1.368, *P* = 0.006037	ZLRLOD				rs861857	1.774, *P* = 0.00213	ZLRLOD
Xp22.33	rs311194	1.408, *P* = 0.00544	ZLRLOD						
Xq23	rs5929434	1.1375, α = 0.178	HLOD rec						

### Association analysis

**Figure 4:**
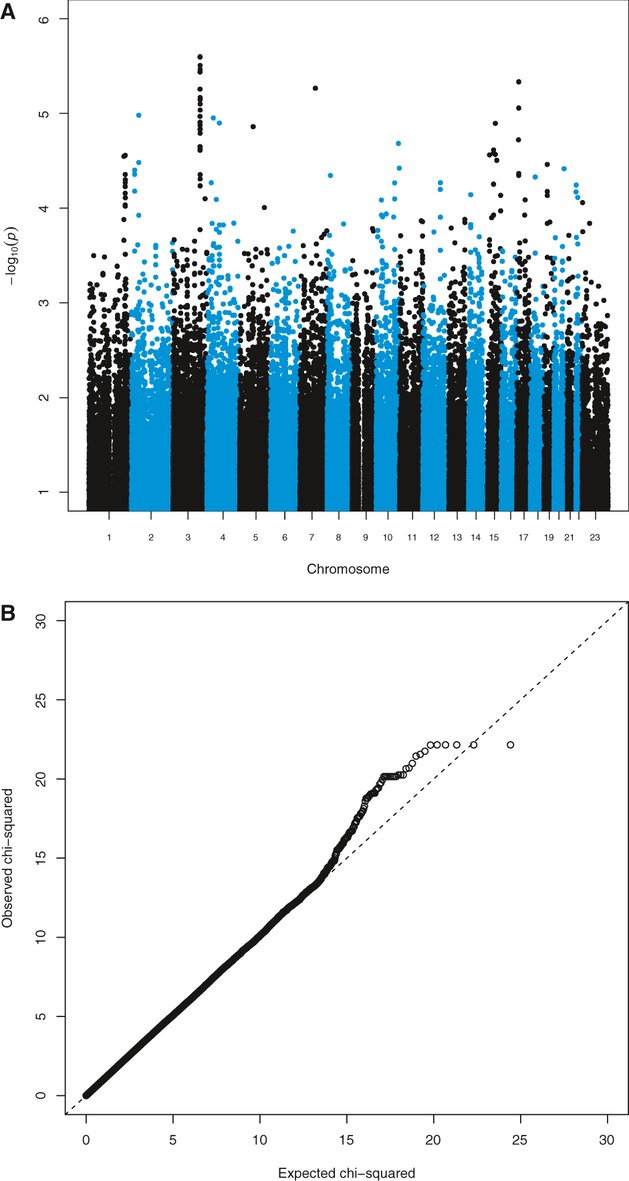
Transmission disequilibrium test results: (A) Manhattan plot–stripes are chromosomes 1–22 and X; (B) QQ plot.

**Figure 5:**
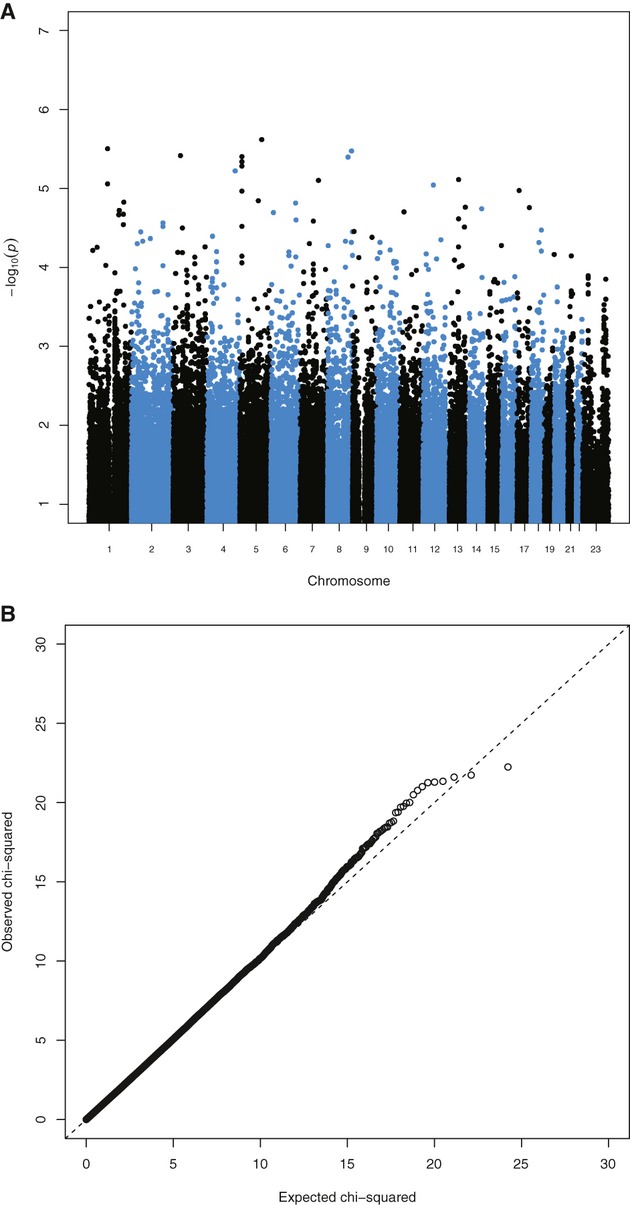
Case–control association results: (A) Manhattan plot; (B) QQ plot.

None of the results of the family-based association study (TDT) reach genome-wide levels of significance. Table 2 shows the results reaching suggestive significance (*P* < 10^−5^) along with results from the previous U.K./Slovenian GWAS analysis (Cordell et al. 2010) for those of our SNPs that they also genotyped. Figure 4 shows Manhattan and QQ (quantile–quantile) plots of our genome-wide TDT results. Table 3 shows the top results using all four computer programs (see Methods) for the case–control association study, while Figure 5 shows Manhattan plots and QQ for one of them (FaSTLMM, results for the other methods were similar, data not shown). Arguably, the most convincing case–control association is on 5p, where there are three adjacent markers reaching suggestive significance, though there are various single markers achieving more significant results on other chromosomes. However, none of the results reported reach genome-wide significance (i.e., would withstand correction for the multiple tests performed). There was no evidence of association with any marker on chromosome X.

**Table 2:** Top results (−log_10_(*P*) > 5) from TDT analysis using PLINK for our own study and from the Cordell et al. (2010) U.K./Slovenian study for SNPs that were also on their array

	This study																UK/Slovenian study		
Ch	SNP	Position (bp, hg19)	A1	A2	T	U	OR for A1 versus A2	CHISQ	PLINK *P*	Unphased allele test *P*	Unphased genotype test *P*	A1 het OR	A1 hom OR	T	U	OR	CHISQ	*P*
3	rs3925065	157667114	A	G	261	164	1.591	22.14	2.54E-06	1.35E-06	7.70E-06	1.580	2.796					
3	rs9290011	157684647	T	C	258	163	1.583	21.44	3.66E-06	1.66E-06	1.03E-05	1.643	2.784	300	273	1.099	1.272	0.2593
3	rs9856195	157693601	G	C	256	163	1.571	20.64	5.54E-06	2.65E-06	1.62E-05	1.642	2.722	298	271	1.100	1.281	0.2577
3	rs827171	157958667	A	G	156	246	0.634	20.15	7.16E-06	6.48E-06	3.58E-05	0.600	0.393					
3	rs827113	157979704	T	C	156	246	0.634	20.15	7.16E-06	6.48E-06	3.58E-05	0.600	0.393					
3	rs13066362	158009477	G	A	156	246	0.634	20.15	7.16E-06	6.48E-06	3.58E-05	0.600	0.393					
3	rs827129	158013249	G	A	156	246	0.634	20.15	7.16E-06	6.48E-06	3.58E-05	0.600	0.393	277	290	0.9552	0.2981	0.5851
3	rs864332	158020378	G	A	156	246	0.634	20.15	7.16E-06	6.48E-06	3.58E-05	0.600	0.393	277	296	0.9358	0.6300	0.4273
3	rs1193510	158030216	G	A	156	246	0.634	20.15	7.16E-06	6.48E-06	3.58E-05	0.600	0.393	283	297	0.9529	0.3379	0.5610
3	rs1193509	158030243	C	T	156	246	0.634	20.15	7.16E-06	6.48E-06	3.58E-05	0.600	0.393	293	306	0.9575	0.2821	0.5953
3	rs827135	158064909	C	T	156	246	0.634	20.15	7.16E-06	6.48E-06	3.58E-05	0.600	0.393	279	288	0.9688	0.1429	0.7055
3	rs827106	158078063	C	T	155	245	0.633	20.25	6.80E-06	6.49E-06	3.59E-05	0.600	0.393					
3	rs6791431	158094357	T	A	160	256	0.625	22.15	2.52E-06	8.60E-07	4.81E-06	0.551	0.346					
3	rs6778370	158094422	C	T	160	256	0.625	22.15	2.52E-06	8.60E-07	4.81E-06	0.551	0.346					
3	rs2362965	158109379	T	A	160	256	0.625	22.15	2.52E-06	8.60E-07	4.81E-06	0.551	0.346					
3	rs9876322	158119238	C	A	160	256	0.625	22.15	2.52E-06	8.60E-07	4.81E-06	0.551	0.346	304	305	0.9967	0.0016	0.9677
3	rs12107103	158126282	T	C	145	231	0.628	19.67	9.20E-06	3.44E-06	1.88E-05	0.564	0.360					
3	rs12107104	158126327	T	G	160	256	0.625	22.15	2.52E-06	8.60E-07	4.81E-06	0.551	0.346					
3	rs4680433	158136848	G	A	160	255	0.628	21.75	3.11E-06	1.09E-06	6.09E-06	0.555	0.350	301	301	1	0	1
3	rs4680436	158159317	G	A	158	252	0.627	21.55	3.45E-06	1.29E-06	7.77E-06	0.570	0.350					
3	rs11714869	158194720	A	G	250	159	1.572	20.25	6.81E-06	1.42E-06	7.12E-06	1.886	2.980					
3	rs939117	158200869	G	C	250	159	1.572	20.25	6.81E-06	1.42E-06	7.12E-06	1.886	2.979					
3	rs4510417	158209553	A	T	248	158	1.570	19.95	7.95E-06	1.59E-06	7.19E-06	1.919	2.972					
7	rs13239130	91046993	T	C	208	125	1.664	20.69	5.41E-06	9.94E-06	3.85E-05	1.821	2.584					
17	rs1859972	12982299	C	A	238	148	1.608	20.98	4.63E-06	8.83E-06	9.62E-08	1.110	3.052					
17	rs11652547	12989386	G	C	235	148	1.588	19.76	8.77E-06	1.63E-05	2.91E-07	1.113	2.924					

Ch, chromosome; A1/A2, minor/major allele; T, transmissions of A1 (from a heterozygous parent to an affected offspring); U, nontransmissions of A1; OR, odds ratio; het, heterozygote; hom, homozygote.

**Table 3:** Top results from the case–control analysis

SNP	Affymetrix ID	Chr	Position (bp hg19)	A1	A2	ROADTRIPS	GenABEL	EMMAX	FaST-LMM	Gene
rs17034354	SNP_A-8672768	1p13.3	109743167	C	A	1.00E-05	5.97E-06	3.43E-06	3.13E-06	*KIAA1324*
rs17034458	SNP_A-8674690	1p13.3	109744268	T	G	1.99E-05	1.61E-05	9.72E-06	8.77E-06	*KIAA1324*
rs13069836	SNP_A-8331728	3p22.1	42307909	G	T	4.40E-06	4.51E-06	4.11E-06	3.84E-06	
rs3774473	SNP_A-2101306	3p21.1	53638851	A	G	9.89E-06	5.96E-05	3.67E-05	3.16E-05	*CACNA1D*
rs4464522	SNP_A-8344968	4p15.1	31240742	C	T	4.27E-06	6.73E-05	4.93E-05	4.03E-05	
rs1458482	SNP_A-2143013	5p15.2	11922555	T	A	5.97E-05	7.25E-06	4.37E-06	3.95E-06	
rs6884647	SNP_A-2127849	5p15.2	11929913	T	G	6.64E-05	8.33E-06	5.05E-06	4.58E-06	
rs1379901	SNP_A-1980728	5p15.2	11949315	T	C	7.91E-05	9.46E-06	5.68E-06	5.19E-06	
rs255630	SNP_A-8298131	5q23.3	127677188	C	T	9.38E-07	1.85E-06	3.31E-06	2.40E-06	*FBN2*
rs11166930	SNP_A-8643016	8q24.3	140824621	C	T	1.59E-06	3.74E-06	4.54E-06	3.35E-06	*TRAPPC9*
rs9635133	SNP_A-8633505	13q34	112988189	G	A	8.24E-06	3.88E-05	2.19E-05	1.73E-05	

Genes are named if the marker is within a gene.

## Discussion

### Linkage results

Having added many more families to the study since our previous genome scan, we had hoped that linkage might improve in some of our linked regions, giving us increased confidence and tighter mapping. Instead, the new families showed linkage at different positions, with only a little, or even no linkage in the regions highlighted in the old families, resulting in modification of the larger peaks in size and position, loss of all of the smaller peaks, and addition of some new peaks in the combined set. Overall, the picture is of a large number of regions of increased allele-sharing by affected family members, with some reaching higher scores than others, but none very high, and it seems that VUR may be even more genetically heterogeneous than we previously supposed, and this makes the results more difficult to interpret.

As our families are predominantly small nuclear families, every family inevitably contributes regions of allele-sharing where VUR genes do not lie. In the nonparametric analysis, as in single-locus LOD analysis, families can contribute negative linkage scores in positions where alleles are not shared, so in regions in which there is in fact no VUR gene, apparent linkage due to chance allele-sharing in some families may be canceled out by lack of any sharing in others. However, as VUR is heterogeneous, some canceling will also occur at loci where there are VUR genes due to lack of allele sharing at those loci by families with mutations at different loci. Thus, in nonparametric analysis of a multifamily cohort for a highly heterogeneous condition, chance can obscure real linkages as well as creating false linkage peaks of similar size.

In parametric linkage analysis, calculation of LOD on the basis of a single dominant gene can result in negative scores across the whole genome if different families actually have mutations at several different loci. This was the case in our original genome scan and again in this one. The HLOD method takes account of heterogeneity, and there are no negative scores, making it possible to achieve positive scores at positions where several sibships share alleles, but it is evident from our own data that the results of combining families that do and do not exhibit sharing at a particular locus are entirely unpredictable. We observe that when there is an HLOD linkage peak in one group of families and no linkage in the other group, the result in all families combined may be that the peak appears again exactly the same size, or half the size or completely disappears, and if there is a small peak in each group, the result in all families combined may also be small, or may be increased. Thus, chance can also have a considerable effect upon parametric linkage results in highly heterogeneous conditions.

As greater numbers give greater statistical power, it seems only sensible to concentrate, for the purposes of further investigation, upon regions in which linkage peaks are seen in the full set of families. These consist of a small number of relatively large (but not highly significant) peaks, and a larger number of very small peaks (Fig. 1). It seems unlikely that the larger peaks are sufficient to account for the positions of the pathogenic mutations in all of the families. Therefore, it seems reasonable to suppose that while some of the small peaks are due to chance sharing of haplotype blocks between siblings, with overlap of shared regions between two or three families, others may be due to the presence of genuine VUR mutations in only two or three families at the particular locus, and from the linkage results alone it is impossible to distinguish between these. As more genes involved in VUR and nonsyndromic CAKUT are discovered, it should become easier to identify likely genuine linkage peaks by the presence of good candidate genes that are similar to the known ones, or that produce proteins or RNAs that interact with them. We have identified relevant genes close to some of our peaks (Table 4), though of course we shall not know which of these are genuine until we have identified pathogenic mutations.

**Table 4:** Correspondences of our linkage peaks to known or candidate urinary tract development genes (names in italics) or linkage peaks (denoted by “HLOD” or “NPL” i.e., nonparametric linkage) or cytogenetic findings (“cyto”) or copy-number variants (CNV) of other studies

Chr pos	Marker	hg19 position	Linkage score	Analysis	Group	Gene/linkage/cyto	Distance	References
1q24.1	rs2281962	167,059,760	1.782, *P* = 0.002086	ZLRLOD	All	* *		
1q24.3	rs12401697	171,388,270	1.534, *P* = 0.00393	ZLRLOD	Old	* *		
2p25.1	rs7569485	9,738,774	1.469, *P* = 0.004642	ZLRLOD	New	* *		
2q37.2	rs1881187	236,567,730	3.341, *P* = 4.38 × 10^−5^	ZLRLOD/dom	A/O	Cyto	0	1
2q37.3	rs6543544	239,892,918	1.7681, α = 0.3139	HLOD dom	Old	* *		
3q12.2	rs7429915	100,284,007	1.7189, α = 0.1783	HLOD dom	All	* *		
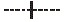	rs6803634	100,869,854	1.4233, α = 0.1958	HLOD dom	Old	* *		
3q27.3	rs10513807	186,656,113	1.295, *P* = 0.0073	ZLRLOD	New	*ETV5*	829.2 kb	2
4p16.3	rs3762867	329,686	1.247, *P* = 0.00829	ZLRLOD	New	* *		
4q22.3	rs11729799	97,070,789	1.7548, α = 0.3965	ZLRLOD/dom	New	*BMPR1B*	991.2 kb	3-5
4q25	rs3866823	111,782,436	2.8711, α = 0.4747	Dom/ZLRLOD	New	*PITX2*	219 kb	6 7
4q34.2	rs17063114	177,401,450	1.9684, α = 0.2731	Dom/ZLRLOD	New	* *		
4q34.3	rs2378811	179,110,516	1.3471, α = 1	HLOD rec	New	* *		
5q14.1	rs6867021	79,229,646	1.164, *P* = 0.0103	ZLRLOD	New	NPL	2.56 Mb	8
6q25.2	rs9478613	155,424,250	2.1001, α = 0.3201	HLOD dom	Old	* *		
7q36.2	rs6973441	153,231,487	2.306, *P* = 0.000559	ZLRLOD	All	CNV, Cyto	0, 0	9, 1
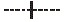	rs2533241	153,421,319	2.922, *P* = 0.000122	ZLRLOD/dom	Old	* *		
8q24.23	rs11776993-	134,555,009-	1.6894, α = 0.4702	HLOD rec	A/O	*KHDRBS3*	0	10
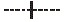	rs4397435	138,367,692	1.7232, α = 0.4985	HLOD rec	A/O	* *		
10q21.3	rs2244205	65,677,634	1.5084, α = 0.2385	HLOD dom	New	* *		
10q25.1	rs1245911	111,533,009	1.7553, α = 0.2346	Dom/ZLRLOD	A/O	* *		
10q26.11	rs10886146	119,579,602	2.2317, α = 0.2623	Dom/ZLRLOD	A/O/N	*EMX2*	270.5 kb	11, 12
10q26.13	rs4962418	126,697,086	2.3394, α = 0.2868	Dom/ZLRLOD	All	HLOD	583.3 kb	13
10q26.3	rs4751013	130,639,298	2.183, *P* = 0.007608	ZLRLOD/dom	Old	* *		
11q25	rs497747	131,796,712	1.03, *P*** = **0.01472	ZLRLOD	New	* *		
12p13.31	rs12582976	5,660,337	1.7686, α = 0.369	HLOD dom	New	* *		
12p13.2	rs7137455	12,664,203	1.122, *P* = 0.01149	ZLRLOD	New	* *		
13q33.2	rs9514424	106,411,268	1.236, *P* = 0.008512	ZLRLOD	Old	NPL, NPL, cyto	619.5 kb, 1.48 Mb, 0	8, 14, 15
15q26.2	rs6416595	94,368,497	1.2433, α = 0.1198	HLOD dom	Old	*RGMA*	736 Kb	16
16q24.1	rs7197843	84,742,878	1.213, *P* = 0.009058	ZLRLOD/dom	Old	*FOXC2*	1.86 Mb	17
19q13.11	rs529579	34,668,294	1.006, *P* = 0.01569	ZLRLOD	All	cyto	0	18
19q13.33	rs352822	49,759,232	1.301, *P* = 0.007184	ZLRLOD	Old	* *		
20p12.1	rs2876409	15,467,075	2.167, *P* = 0.000792	ZLRLOD	Old	CNV, cyto	0, 0	9, 19
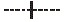	rs6110544	15,166,775	1.7246, α = 0.3133	HLOD dom	Old	* *		
21q22.3	rs8129605	45,617,878	1.252, *P* = 0.008167	ZLRLOD	Old	CNV	0	9
22q11.21	rs5746685	19,192,596	1.368, *P* = 0.006037	ZLRLOD	All	*TBX1*, CNV, cyto	551.6 kb	20, 9, 21, 22
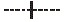	rs861857	21,982,340	1.774, *P* = 0.00213	ZLRLOD	New	* *		
Xp22.33	rs311194	2,720,702	1.408, *P* = 0.00544	ZLRLOD	All	*PRKX*	801.7 kb	23–28

“0” indicates that the gene is within the linkage plateau, or that the linkage peak is within the CNV or cytogenetically defined region. Key to references: 1, Weber et al., 2011; 2, Costantini 2010; 3, Miyazaki et al. 2000; 4, Weber et al. 2008; 5, Martinez et al. 2001; 6, Acharya et al. 2011; 7, Hasegawa et al. 2010; 8, Briggs et al. 2010; 9, Sanna-Cherchi et al. 2012; 10, Ashraf et al. 2009; 11, Miyamoto et al. 1997; 12, Boualia et al. 2011; 13, Cordell et al. 2010; 14, Vats et al. 2006; 15, Freedman et al. 2005; 16, Xia et al. 2007; 17, Kume et al. 2000; 18, Davidsson et al. 2010; 19, Stefanou et al. 2006; 20, Fu et al. 2012; 21, Lipson et al. 1991; 22, Wu et al. 2002; 23, Li et al. 2002; 24, Klink et al. 1995; 25, Schiebel et al. 1997; 26, Kolon et al. 1998; 27, Rosser et al. 2009; 28, Naseri et al. 2010.

In the nonparametric linkage analysis, the main peaks from our previous study (on 1q, 2q, 6q, 7q, and 10q) have become only slightly larger or slightly smaller in the combined data, as well as shifting slightly in position, and all the smaller peaks have fallen below recognition level of *P* < 0.02, but two new peaks have been added on 19q and 22q.

In the dominant HLOD analysis, the same pattern occurs, but there is a slightly different collection of most prominent peaks. The 10q peak is larger than the 2q one, instead of the other way round, as in the nonparametric analysis, but they are again the largest two. However, the peak on 1q, though present in both old and new families, never reaches an HLOD score of 1.2, even in the combined result. The peak on 6q becomes smaller, and the one on 7q, seen in the old families, is reduced to 0.82 by the addition of the new families, but there is a peak on 3q in the old families which is slightly increased in the combined data, with a small contribution from the new families. As in the nonparametric analysis, there are peaks of linkage in the new families that did not appear in the old families, but none of these survives >1.2 in the combined results, while the peak on 22q seen in the nonparametric analysis, reaches only 0.34 in the all families HLOD dominant analysis.

The very small amount of recessive linkage does not account for much of the difference between the results of the nonparametric and dominant HLOD analyses, either in magnitude or position. For instance, in the combined data the linkage on 1q reaches 1.78 with a p value of 0.002 in the nonparametric analysis (and is on 1q24.1), but only reaches 0.74 HLOD (and that 3.3 Mb away on 1q23.3), while on 3q12.2 nonparametric linkage only reaches 0.86 with *P* = 0.024, yet it reaches HLOD 1.72 in exactly the same position. This may be due partly to differences in the ways that the different statistical methods work, but also to the particular values chosen for parameters such as allele frequency and penetrance for the HLOD analyses.

**Figure 6:**
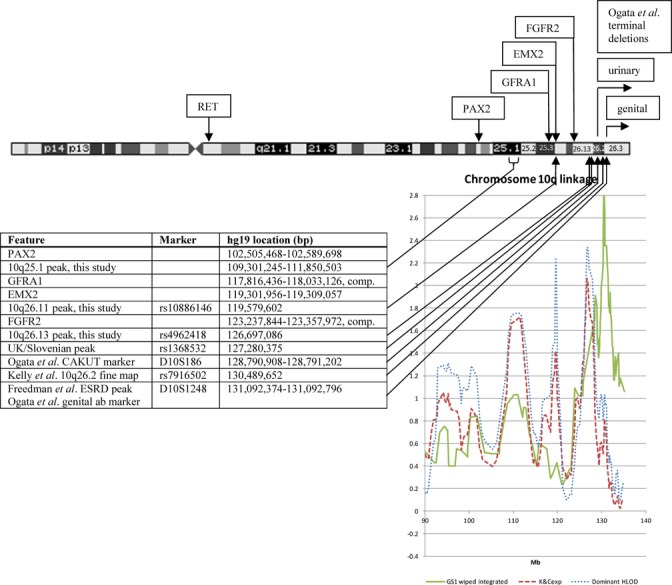
Chromosome 10 showing the relationship of genetic results to the genes known to be involved in urinary tract development. The sharp spikes of linkage are each composed of several markers and the marker with the highest linkage is quoted. Solid line, “Kelly et al. (2007) fine map” in the table, “GS1 wiped integrated” – After the scan published as Kelly et al. (2007) (genome scan 1, GS1) further markers were genotyped for fine mapping, and this is the result of a “wiped” analysis of the combined sets of original and fine-mapping markers; dashed line, nonparametric (Kong and Cox exponential, all families in the present study); dotted line, HLOD dominant (all families).

On chromosome 10, there are five genes that are already known to be involved in urinary tract development, mainly from mouse work, and there appears to be another, as yet unidentified, near the q telomere, because a series of patients with terminal deletions of 10q and urinary tract and/or genital anomalies was reported (Ogata et al. 2000) including cases with VUR. The known genes were found to be present in two copies in these patients, and the authors identified the limits of deletion in the collected urinary tract anomaly patients and in the overlapping set of genital anomaly patients by microsatellite markers. The marker for the former is slightly further from the telomere than the latter, and the authors commented that there might be a single gene or two different genes accounting for these anomalies. Subsequently, the results of a genome scan for end stage renal failure were published (Freedman et al. 2005), and the marker of one of the peaks of linkage was the same as the more terminal of the two markers of the deletion study (see Fig. 6). It seems reasonable to suppose that this might point to the same gene, as VUR/CAKUT is one of the causes of renal failure. Our linkage peak in our first genome scan (refined subsequently by fine mapping) was also inside the published deletion region, and we presumed that it also indicated the same gene. However, in the present study, our peak dominant HLOD (2.34) and peak NPL (2.06) on 10q are both at rs4962418, which is about 4 Mb proximal to our previous result, outside the deletion region and closer to one of the known genes, *FGFR2*. It is also only about 600 kb from rs1368532, the peak dominant marker (HLOD 2.44) on 10q in the joint U.K.-Slovenian data (Cordell et al. 2010). Mice with conditional deletion of *Fgfr2* in metanephric mesenchyme are prone to develop VUR (Hains et al. 2010), so it may well be that the mutations that we are seeking in this region cause downregulation of *FGFR2* in the developing urinary tract. However, the standard deviation of peak linkage position at such scores is around 15–20 cm (Cordell 2001) so it is still an open question as to where the mutations may lie. Indeed, in the present study, there is another spike of dominant linkage (HLOD 2.23) about 7 Mb toward the centromere, and close to *EMX2*, and another of dominant and nonparametric linkage of 1.75 and 1.72, respectively, between *PAX2* and *GFRA1*, so it may even be that the mutations indicated by linkage of VUR to 10q do not all relate to the same gene. Figure 6 illustrates all these findings.

Though the linkage close to *EMX2* is only a very narrow spike, the peak marker is the only one of the 7051 genome-wide markers used for linkage that achieves a ZLRLOD result with *P* < 0.02 in both the old and new groups of families (see Fig. 6 and Tables 1 and 4). It is at the edge of a highly conserved noncoding region including a DNAse I hypersensitivity cluster recognized by the ENCODE Project (Maher 2012), and we are hopeful of finding mutations in this region in some of our families.

In contrast to 10q, our other main linkage peak, on 2q (by far our most significant with *P* = 4.38 × 10^−5^), is not close to any known urinary-tract developmental genes and linkage has not been found by any other group studying VUR. The same applies to our nonparametric linkage regions on 1q24.1, 4p16, and 7q36.2. However, since our first genome scan, in which the 2q37 and 7q36 linkage peaks were both found, a patient with duplex kidney and VUR (and microcephaly, psychomotor retardation, and dysmorphic features) has been found to have an unbalanced 2;7-translocation with a terminal 9.52 Mb gain in chromosomal band 2q37.1-q37.3 and a terminal 5.65 Mb loss in 7q36.2-q36.3 (Weber et al. 2011), possibly confirming at least one of these loci. Both of them are amongst nine of our linkage peaks that lie in the same broad chromosomal bands (2p25, 2q37, 4p16, 5q14, 7q36, 20p12, 21q22, 22q11, and Xp22) as genomic copy-number variations (CNVs) identified in a recent association study of patients with renal hypo/dysplasia (Sanna-Cherchi et al. 2012), but our linkage peak on 7q36.2 is one of four that lie within the described CNVs, the others being those on 20p12.1, 21q22.3, and 22q11.21. Thus, there is some support from cytogenetic or CNV results for some of our linkage peaks that are in positions in which other studies have not found linkage.

Our recessive linkage peak on 8q24.23, though only representing three families, and mainly just one of them, is clearly interesting because it agrees exactly with a previous report (Ashraf et al. 2009). The authors sequenced all exons of the only gene in their linkage region, *KHDRBS3*, and found no mutation, suggesting that a regulatory mutation might be the cause of the phenotype. We have found no mutation in the 5′-untranslated region nor in 1 kb of promoter sequence in our three families, and will probably need to search the introns and several megabases of sequence beyond the gene for a possible enhancer or repressor mutation in addition to sequencing the exons in all of our three families. Other correspondences between our linkage peaks and those of other studies, or with cytogenetic findings or candidate genes are shown in Table 4. Because our linkage scores are low, and therefore inevitably some will be false positives, we cannot be certain which may be real, but there is just one other that seems likely to be real, though small, and may even prove to be the most interesting finding in the study.

Very close to the small nonparametric linkage peak on the X chromosome is the gene *PRKX*, encoding a serine–threonine protein kinase that is developmentally regulated and, in the developing urinary tract is restricted to the ureteric bud epithelium, and may be involved in renal epithelial morphogenesis (Li et al. 2002). Abnormal recombination between this gene and a related pseudogene on chromosome Y is a frequent cause of sex reversal disorder in XX males and XY females (Klink et al. 1995; Schiebel et al. 1997). This is interesting because in the course of our quality control checks on our SNP genotyping data, we found a sample for which phenotypic and genotypic gender did not match. Microsatellite analysis confirmed that this child was an XX male. The child was investigated and found to have an Yp;Xp translocation, a normal X and no Yq. Whether a mutation of *PRKX* is related to the VUR in this boy and his sister is currently unknown, but it is an intriguing possibility. It would have to be a dominant mutation, and on the father's X chromosome, if it also increased the liability to translocation in the generation of a gamete. However, this is perfectly possible as (a) X-linked dominant VUR has recently been reported (Naseri et al. 2010) and it can be seen from Figure 2 that our linkage is dominant, and (b) VUR has previously been reported in an XX male (Kolon et al. 1998). The instability region is not inside the gene but adjacent to it on the centromeric side (Rosser et al. 2009). This means that the gene would be lost in the translocated chromosome of the boy, but it is possible that the same mutation that increased the instability of the instability region could inactivate the adjacent copy of the gene in his sister. (The outcome after that might depend upon X-chromosome inactivation).

Though we found linkage fairly close to two of the nonparametric linkage peaks found by Briggs et al. (2010) (see Table 4), our results did not replicate the third, on chromosome 18. Neither did we find linkage corresponding to peaks on 1p33-p32 (Sanna-Cherchi et al. 2007), 1p36.2-p34.3 nor 4q26-q32.3 (Conte et al. 2008). The latter lay between linkage peaks on 4q in our families. Neither did we have linkage corresponding to the recessive peak of Weng et al. (2009) on 12p11-q13 nor the recessive peaks of Cordell et al. (2010) on 3q or 5q, though our nonparametric linkage peak on 19q in the old families (at 49,759,232) is only about 3.4 Mb from their recessive linkage peak on that chromosome (at 53,149,967) and our nonparametric peak on 2p in the new families (at 9738,774) is only 2 Mb from their recessive peak at 11,808,864. Thus, overall, though we found linkage in some positions where other studies found linkage, and linkage close to but not overlapping other places where others found linkage, our study did not confirm various others at all.

In summary (1) We found almost no correspondence between the linkage patterns in the two groups of families (“old” and “new”) from the same population, with just a single SNP, close to *EMX2* achieving *P* < 0.02 in both groups in one analysis type, ZLRLOD. (2) Though some of our linkage peaks corresponded with linkage peaks of other studies, we did not replicate many of other results and found various results not found in other studies. (3) The combined conclusion of points 1 and 2 is that VUR is extremely genetically heterogeneous. However, it is also no doubt true that some of the apparent linkage peaks in our own and other studies will later prove to be artifacts, but at this stage it cannot be determined which ones these will be. (4) Each of our linkage analyses detected some linkage in places where linkage was not found by the other analyses. (5) Some of our linkage peaks were close to genes known to be involved in urinary tract development, and some were close to plausible new candidates, but in other peaks, including our highest linkage, on 2q, there is no obvious candidate.

### Association results

Although none of the results from our genome-wide association analysis are highly compelling, some are worthy of further discussion. Firstly, the result of 23 adjacent suggestively significant SNPs on 3p in the TDT analysis is very interesting. Most of these markers are in the gene *RSRC1*, which produces a protein that takes part in RNA splicing, something that cannot be ruled out from relevance to VUR as another RNA-splicing protein KHDRBS3 appears to be involved in VUR, but adjacent to *RSRC1* is a gene far more likely to be involved in VUR, and it is *SHOX2*. *Shox2* regulates *Bmp4* expression in the developing mouse heart (Puskaric et al. 2010), and *Bmp4* is also one of the regulators of ureteric bud growth (Miyazaki et al. 2000). Furthermore, GUDMAP (Genitourinary development molecular anatomy project) (McMahon et al. 2008; Harding et al. 2011) kidney development arrays show that *Shox2* is expressed in the metanephric mesenchyme (which secretes factors that regulate ureteric bud outgrowth) so it is highly likely that *SHOX2* also regulates *BMP4* in the developing urinary tract, and therefore could be involved in VUR. The gene lies between the third and fourth SNPs listed in Table 2. Our results for the first three SNPs show a clear dosage effect, the odds ratio for the homozygotes being almost twice that for the heterozygotes. The results for the last three SNPs are similar, though not quite as marked, the odds ratios being about 1.5 times as great as for the heterozygotes. These results suggest the possibility that there might be relevant regulatory elements in these areas of the genome. However, there was no association between VUR and this region in the U.K./Slovenian study (Table 2), so either the association in our data is due to chance (not unlikely, in view of the fact that it does not reach a genome-wide level of significance) *or* there is a particular mutation which is common in Ireland on one haplotype bearing these SNPs while in the U.K. and Slovenia mutations on other haplotypes are as common and balance the ratio so that no association is seen, *or* the difference is due to the fact that the U.K./Slovenian collaboration deliberately excluded families in which the index case or an affected sibling had additional structural anomalies of the urinary tract, whereas we intentionally included them.

None of our other mildly significant TDT results can be checked against the U.K./Slovenian study because two of the SNPs were not on their array and the other failed quality control. The closest gene to the two adjacent markers on chromosome 17 is *ELAC2*. GUDMAP shows that it is highly expressed in the ureteric bud, and is also expressed in the metanephric mesenchyme. The protein produced by this gene interacts with SMAD2, though so far only SMAD4 has been implicated in urinary tract development (Oxburgh et al. 2004). The other two markers had no significant neighbors. None of the genes on either side of the one on Chromosome 4 appear related to urinary tract development, but the nearest gene to the one on Chromosome 7 (7q21.13), less than 150 kb away, is *FZD1* (Frizzled 1). The frizzled proteins act as receptors in Wnt signaling (first established in Drosophila [Bhanot et al. 1996]) and WNT signaling is involved in regulating ureteric bud growth (see review [Michos 2009]). GUDMAP shows that *Fzd1* is expressed in the metanephric mesenchyme at mouse embryonic day 11.5, so this would be an intriguing avenue for further investigation, should this association be genuine.

Turning to the case–control association results, there is just one instance of three adjacent markers achieving the suggestive significance threshold of *P* < 1 × 10^−5^ and the most significant of these is just 18 kb from *CTNND2*, Catenin δ2. This is a member of the armadillo/β-catenin superfamily and has been implicated in brain and eye development (Zhang et al. 2010) (just as various other genes are known to be involved in the development of the urinary tract and eye or brain, e.g., *PAX2*, *FOXC2,* and *SHH*). It has also been found to be highly enriched in the ureteric bud tip compared with the ureteric stalk, and to be one of the most highly induced targets of GDNF signaling in the mouse ureteric bud (embryonic day 11.5) (Schmidt-Ott et al. 2005) (and GUDMAP confirms its expression at this time as well as being strongly expressed specifically in the ureteric tip on day 15.5) so it is a good candidate. There are two adjacent significant results on 1p13.3 in *KIAA1324 alias EIG121L*, “Estrogen-induced gene 121-like,” which has been found to be expressed in various tissues during early Xenopus development (Araki et al. 2007) but has not so far been implicated in mammalian urinary tract development.

All the rest of the significant results are from isolated SNPs (whose adjacent SNPs show some elevation but do not reach the threshold). Whereas, apparently significant SNPs whose adjacent SNPs showed no association at all were rejected, and not reported, as the results most likely resulted from incorrect genotyping (see the very last paragraph of the Materials and methods section), these SNPs, with some elevation of probability of neighboring SNPs, represent the lowest level of possible real association. Some of them are within 0.5 Mb of possible candidate genes (Table 5) and several others (not identified in the table) are within 2 Mb of good candidate genes, but this may just be chance. There are already many genes known to be involved in urinary tract development, and these are spread across the genome. Therefore, if one were to choose points at random in the genome, some would come close to such genes by chance. For this reason, we can never be sure whether a weak association or a small linkage peak is real until the pathogenic mutations have been identified and proven, and all that a study such as ours can ever do is to suggest parts of the genome in which to start searching for mutations, which was of course the aim of the study. Indeed it is all that any linkage or association study can do, but, in a disorder of high genetic heterogeneity such as VUR, the results are inevitably all the more precarious.

**Table 5:** Candidate genes within 1 Mb of top markers in the case–control association analysis

SNP	Chr	roadtrips	genabel	emmax_BN	FastLMM_RRM	Candidate gene	Distance	References	Exp
rs17034354	1p13.3	*–*	5.97E-06	3.43E-06	3.13E-06				
rs17034458	1p13.3	*–*	*–*	9.72E-06	8.77E-06				
rs13069836	3p22.1	4.40E-06	4.51E-06	4.11E-06	3.84E-06				
rs3774473	3p21.1	9.89E-06	*–*	*–*	*–*				
rs4464522	4p15.1	4.27E-06	*–*	*–*	*–*	*PCHD7*	92 kb	29	✓
rs1458482	5p15.2	*–*	7.25E-06	4.37E-06	3.95E-06	*CTNND2*	18 kb	30, 31	✓
rs6884647	5p15.2	*–*	8.33E-06	5.05E-06	4.58E-06	*CTNND2*			
rs1379901	5p15.2	*–*	9.46E-06	5.68E-06	5.19E-06	*CTNND2*			
rs255630	5q23.3	9.38E-07	1.85E-06	3.31E-06	2.40E-06				
rs11166930	8q24.3	1.59E-06	3.74E-06	4.54E-06	3.35E-06	*TRAPPC9*	0	32	✓
rs9635133	13q34	8.24E-06	*–*	3.46E-12		*SOX1*	262 kb	33, 34	✓

Exp, expression at relevant time in relevant tissues in mouse embryo recorded in GUDMAP. Key to references: 29, Berndt et al. 2011; 30, Zhang et al. 2010; 31, Schmidt-Ott et al. 2005; 32, Yoshida et al. 2009; 33, Gimelli et al. 2010; 34, Reginensi et al. 2011.

### Future studies

As we have said, a linkage or association study can only show one where to look for mutations. The next step is to sequence the DNA in the promising areas. Then comes the more difficult step of identifying which of the genetic variants may be pathogenic, from the very large number of variants that will inevitably be found. It is easy enough to assign pathogenicity for nonsense mutations, homozygous missense mutations, and splice-site mutations, but for heterozygous missense mutations (remembering that most VUR is autosomal dominant), functional studies are necessary, as one cannot just assume that the normal allele on the other chromosome will not be sufficient, or that it will be inhibited by the product of the mutated allele. For variants in noncoding DNA, where we expect the majority of mutations to be, it can be difficult even to guess which variants are worth investigating. The ENCODE project (Maher 2012) has identified many potential regulatory regions across the genome, but every cell type at every stage of differentiation and function uses different sets of regulators, and the results so far, though extensive, are far from covering every type of investigation in every type of cell at every stage of development, so many of the regulatory DNA sequences relevant to VUR have probably not yet been defined as regulatory elements.

The first step after sequencing and identifying variants, is to “filter” the variants by reference to the SNP database (dbSNP) and other variant databases, by checking the evolutionary conservation of the bases concerned, and by using all the pathogenicity predictors available, including location within likely regulatory elements defined by ENCODE. A systems biology approach might also help. Pathway software might give clues about the affected pathways in VUR by finding interactions between unsuspected candidate genes close to our association results and within our linkage regions, and visualize them in a molecular network. However, many genes still have very little annotation and they would not be included. Then from the shortlist of variants in highly conserved positions that are absent from or rare in variant databases, the next step is to check whether each variant segregates with VUR in the family or families in which it is found. Then, for those that do, the next step is to screen control DNA samples from the same population, and for this we have Irish BioBank controls from blood donors. This will eliminate any variants that are just as common in the general Irish population as they are in VUR patients. After that the remaining variants have to be tested by making the mutations in human cells in culture and observing the effects on gene expression, and after that, the variants that remain promising have to be tested by making transgenic mice and observing whether they develop VUR or CAKUT. We have collaborators ready to do the cell and mouse studies when we have selected promising variants.

As mentioned above, the genotyping for this study revealed that a number of our independently ascertained families are genetically related, and these provide one avenue for study. We have already performed whole-genome sequencing on members of one of these extended families, and screened the variants, including using the SNP genotyping data from the genome scan to identify the genomic regions linked to VUR in that particular family, and are currently carrying out BioBank control screens of the shortlisted variants prior to functional studies. Subsequently, we hope to be able to do the same with DNA from our other extended families. We also intend to sequence the genome right across the major linkage peaks in all our patients, and hope that by this we may be able to discover new relevant regulatory elements by clustering of rare novel conserved variants from different families at particular restricted sites. Then functional studies will again be needed. We also intend to sequence across the 8q recessive linkage region in our three families that show linkage to that, and similarly screen variants found there. As will have been learnt from the linkage and association sections above, there are various other interesting findings which we also hope to pursue in due course.

## Conclusions

The observations that VUR is clearly inherited with high incidence in first degree relatives, and yet linkage studies using large numbers of small families achieve few if any results reaching conventional significance levels, and that studies of smaller numbers of larger families produce conflicting results, can all be explained by there being many genes involved in urinary tract development, disruption of the activity of any one of which may cause VUR and/or CAKUT, so that in large cohorts of families, only a small proportion of them are likely to have mutations at any one locus, and small groups of families will sample different small numbers of loci. Likewise, a candidate gene linkage study is likely to detect very low linkage, even if the gene studied is involved in VUR, because most of the families being tested will be linked to other loci. Also, candidate exon sequencing will often find few mutations (a) because most of the patients have mutations at other loci and (b) because many of the mutations are probably not in the exons, and may be at some distance away from the gene. Most of the conserved DNA in the genome is noncoding, and though it is easy to determine the pathogenicity of a coding mutation, concentrating upon coding sequences inevitably means that most mutations will not be found.

With small linkage peaks in studies such as ours, there is inevitably a low signal-to-noise ratio, such that some genuine linkages may be missed while some spurious peaks may appear, and the accuracy of the position of a linkage peak in relation to the true site of mutations is also low, but some of the findings from this study are so striking that they seem likely to be true, and provide numerous leads for further study.

It will not be easy to identify functional mutations in noncoding sequences and to demonstrate that they do indeed regulate the genes concerned and do lead to VUR and/or CAKUT, but the recent publications from the ENCODE project should at least help us to identify the best candidate regulatory elements. This, combined with the availability of high-throughput DNA sequencing technologies, should allow the causative genetic changes to be identified.

We concluded from the heterogeneity uncovered by our first genome scan (Kelly et al. 2007) that a simple genetic test for VUR would not be possible, but in the meantime technology has advanced rapidly and already there are tests for panels of mutations in multiple genes involved in other disorders, so in time such testing may also become possible for VUR.

## Supporting Information

Figure S1 Information content across the autosomes given by the thinned set of SNPs (n = 7051) used for linkage analysis.

Figure S2 Information content across the X chromosome by the SNPs used for linkage.

Figure S3 HLOD analysis with a dominant model of inheritance for the original families (top) the new and all families together.

Figure S4 HLOD analysis with a recessive model of inheritance.

Figure S5 Nonparametric analyses for the old, new, and all families.

Figure S6 Linkage in all families ______ HLOD dominant; ------- HLOD recessive; ——ZLRLOD.

Figure S7 Linkage in the “old” families ______ HLOD dominant; ------- HLOD recessive; —— ZLRLOD

Figure S8 Linkage in the “new” families ______ HLOD dominant; ------- HLOD recessive; —— ZLRLOD.
