# Supplementary material for: A new genome scan for primary nonsyndromic vesicoureteric reflux emphasizes high genetic heterogeneity and shows linkage and association with various genes already implicated in urinary tract development
Source: Mol Genet Genomic Med. 2013 Jul 7;2(1):7–29. doi: 10.1002/mgg3.22 (PMC3907909; doi:10.1002/mgg3.22)
Supplement: Figure S1 — Information content across the autosomes given by the thinned set of SNPs (n = 7051) used for linkage analysis. [file mgg30002-0007-sd1.pdf]

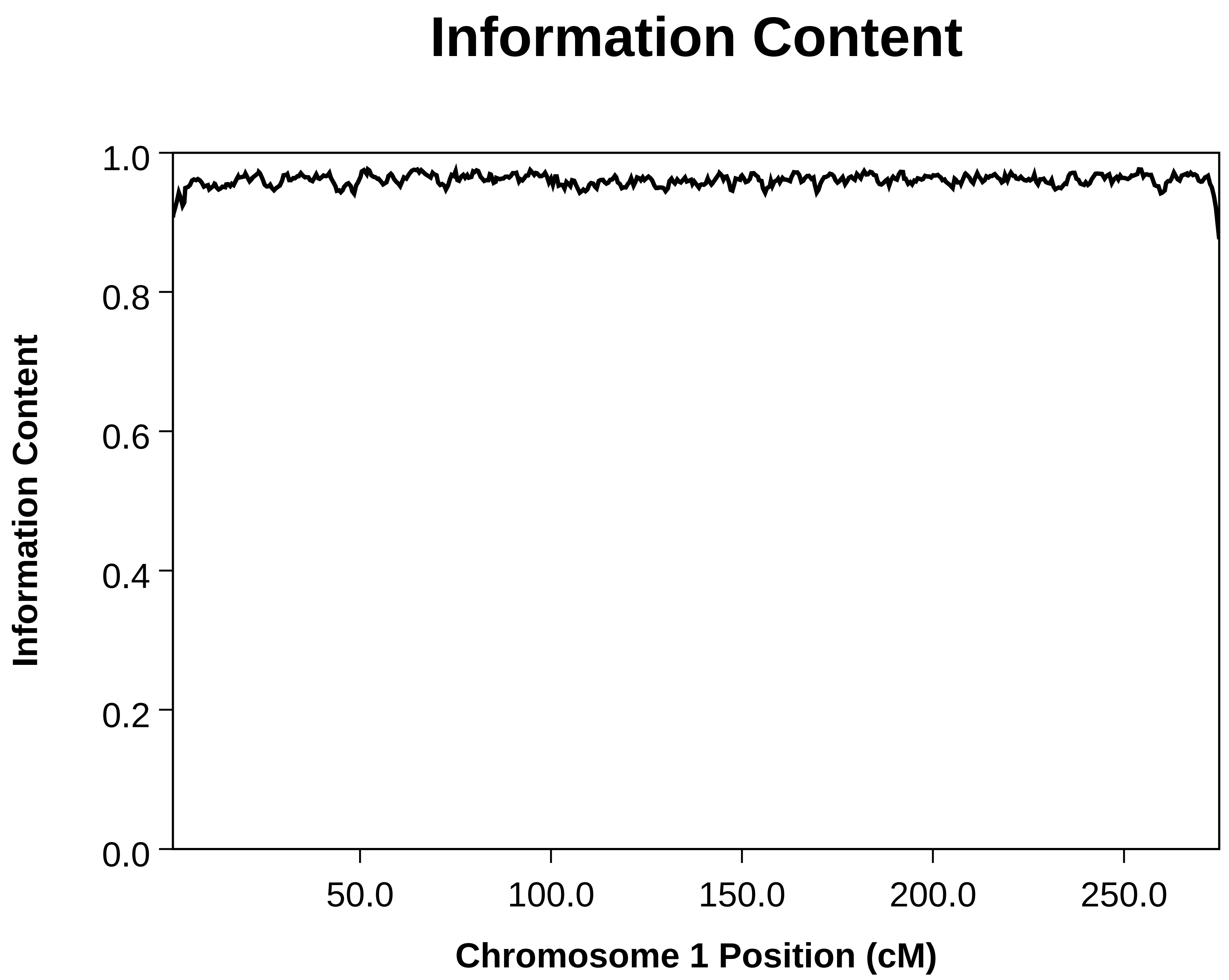

Information Content
1.0
0.8
0.6
0.4
0.2
0.0
50.0
100.0
150.0
200.0
250.0
Information Content
Chromosome 1 Position (cM)

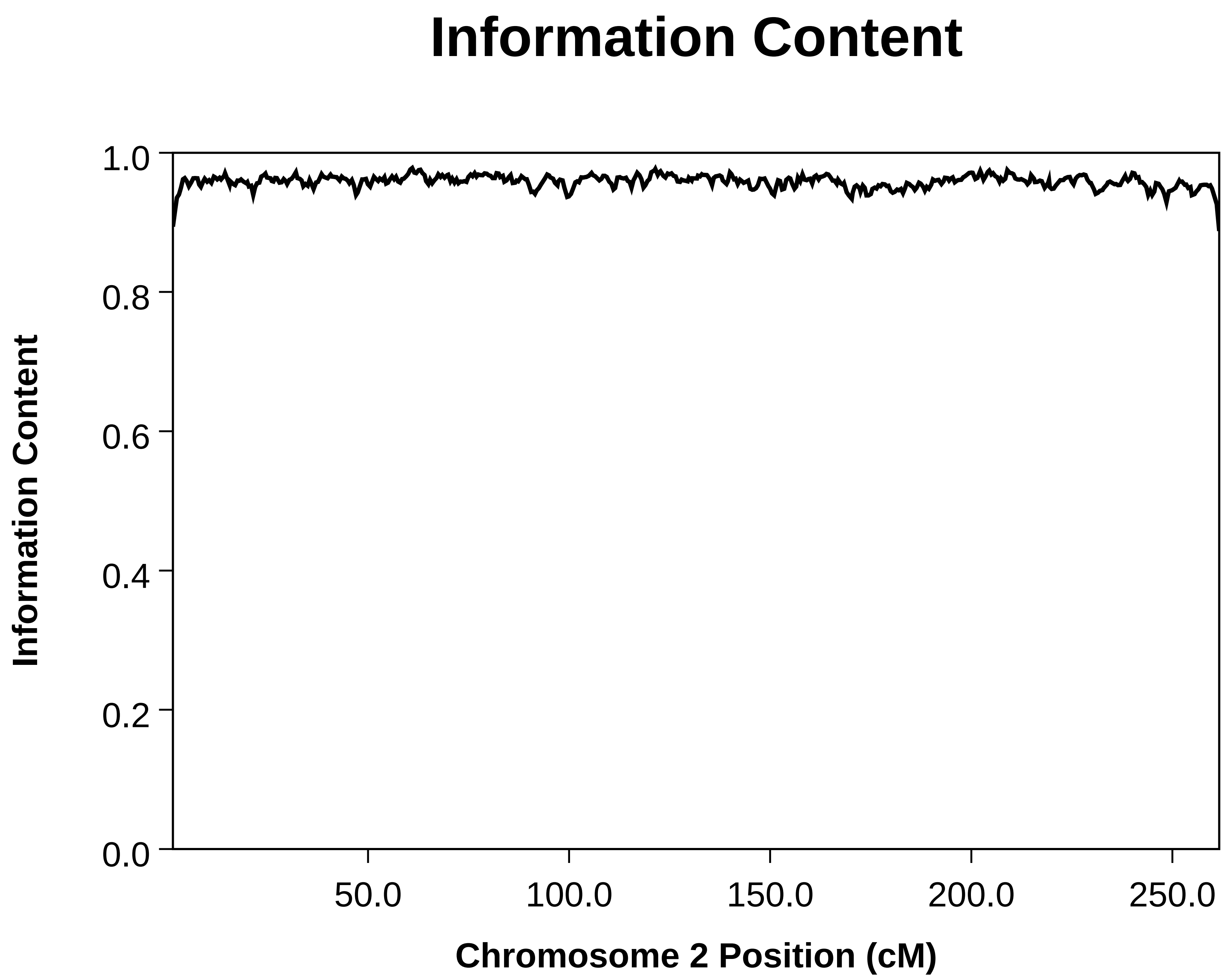
Information Content
1.0
0.8
0.6
0.4
0.2
0.0
Information Content
50.0
100.0
150.0
200.0
250.0
Chromosome 2 Position (cM)

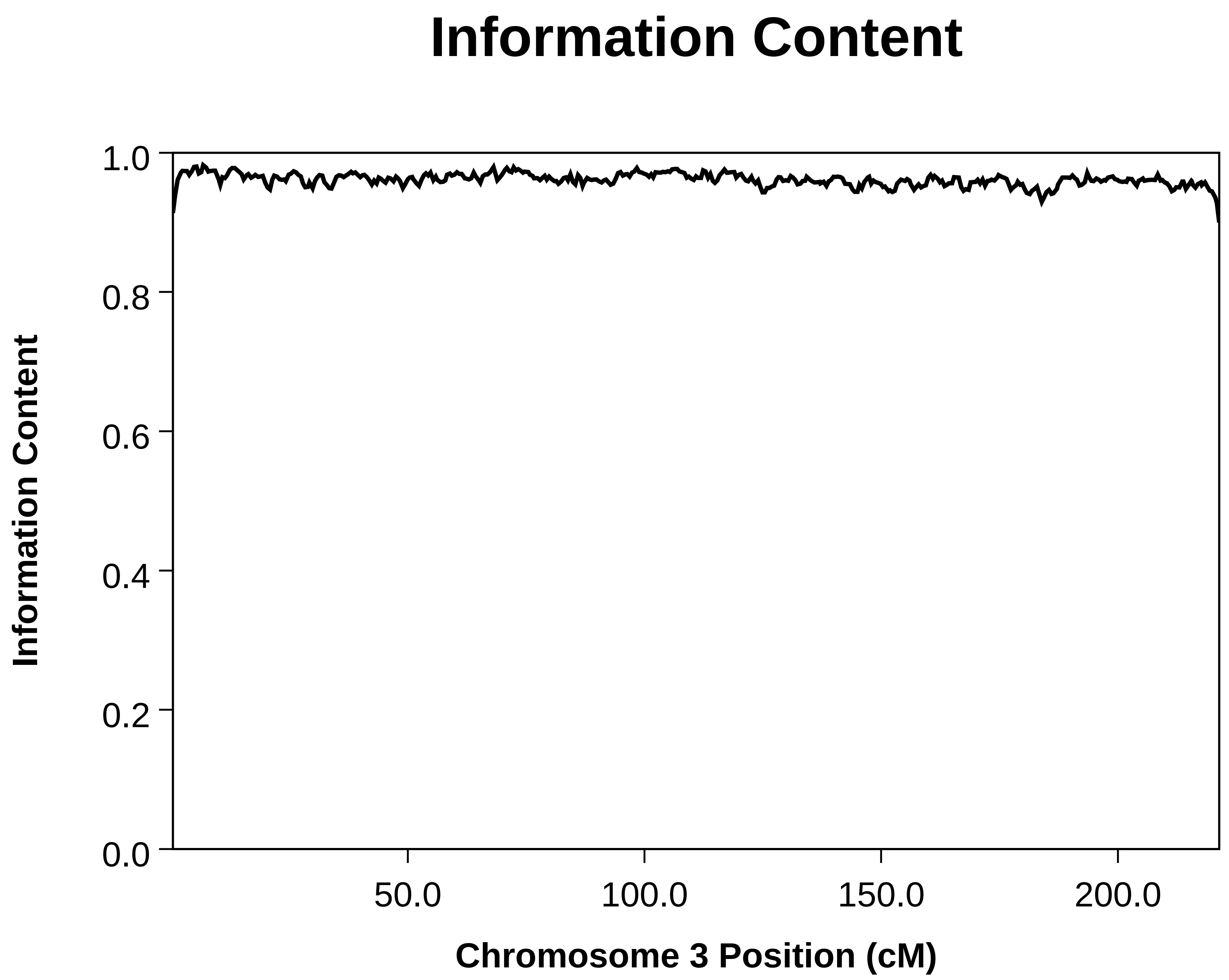
Information Content
1.0
0.8
0.6
0.4
0.2
0.0
50.0
100.0
150.0
200.0
Chromosome 3 Position (cM)
Information Content

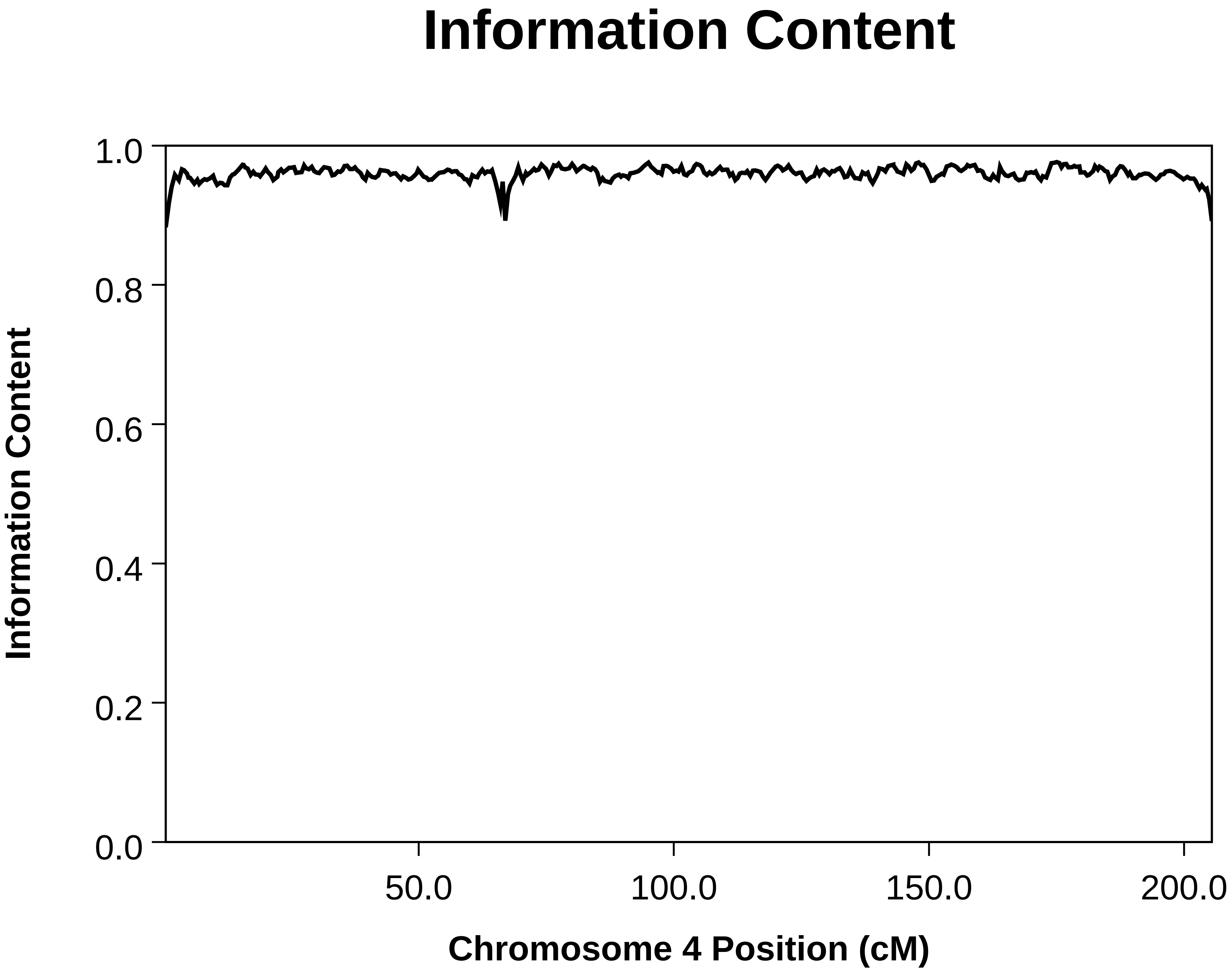
Information Content
1.0
0.8
0.6
0.4
0.2
0.0
Information Content
50.0
100.0
150.0
200.0
Chromosome 4 Position (cM)

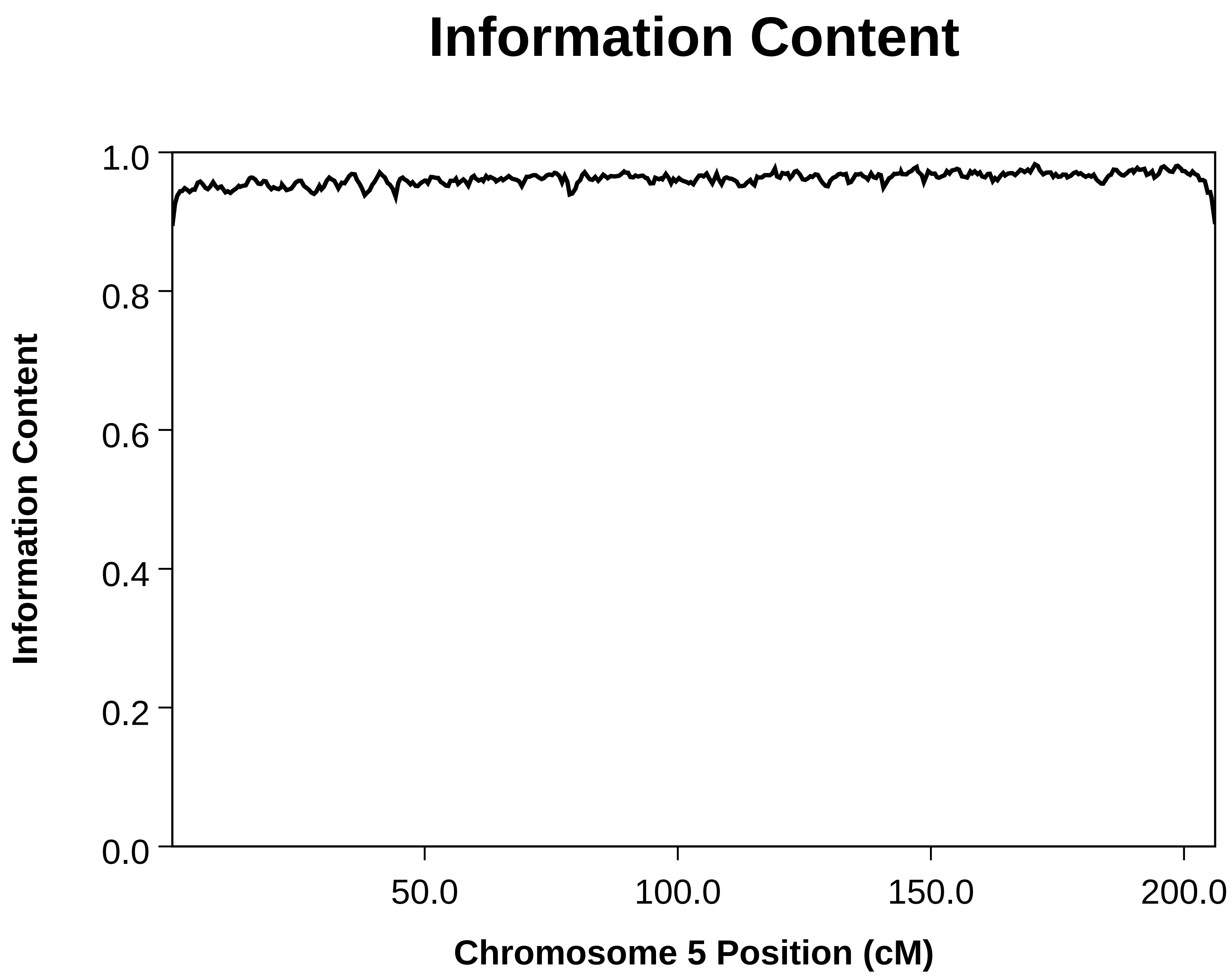
Information Content
1.0
0.8
0.6
0.4
0.2
0.0
50.0
100.0
150.0
200.0
Chromosome 5 Position (cM)
Information Content

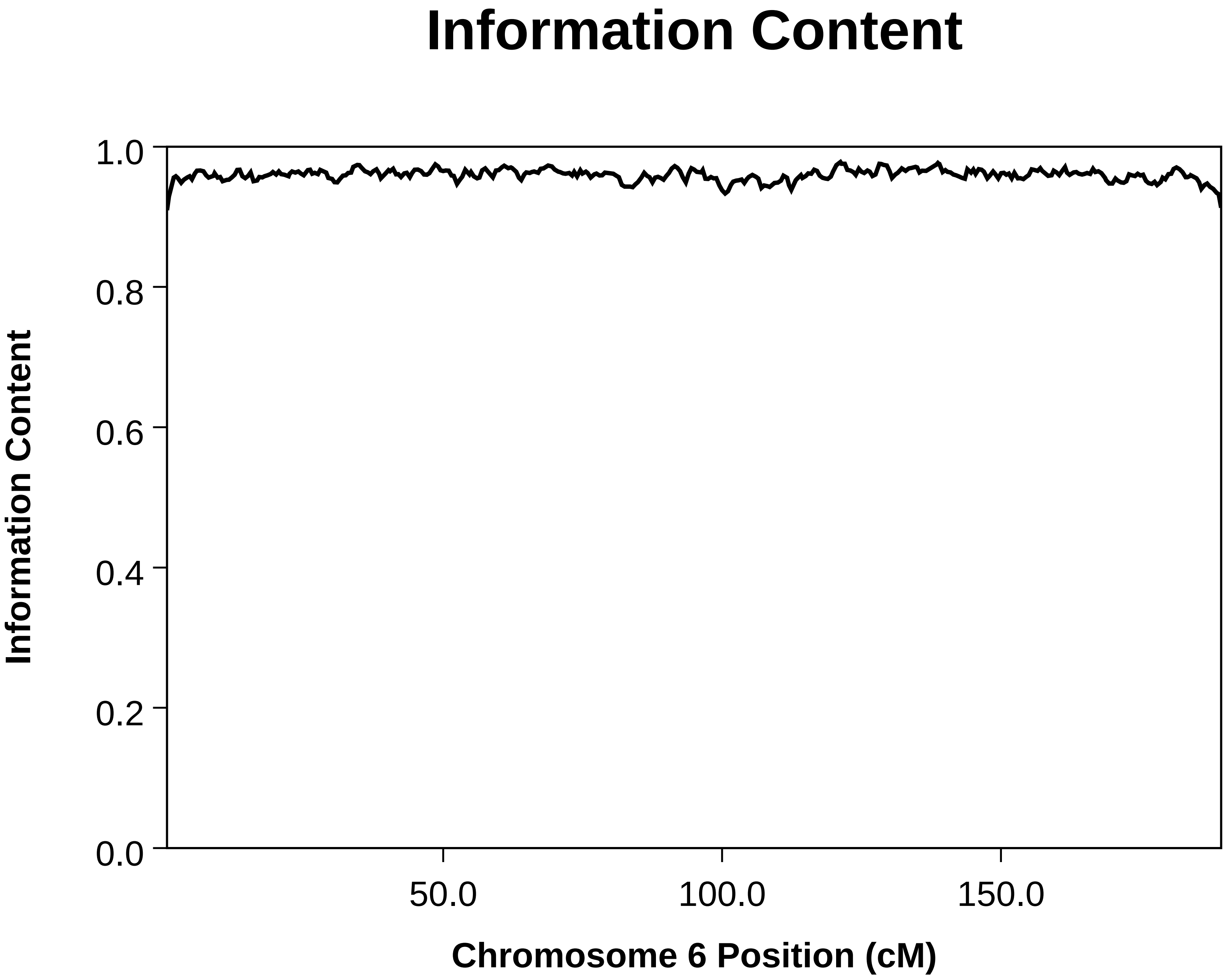

Information Content
1.0
0.8
0.6
0.4
0.2
0.0
Information Content
50.0
100.0
150.0
Chromosome 6 Position (cM)

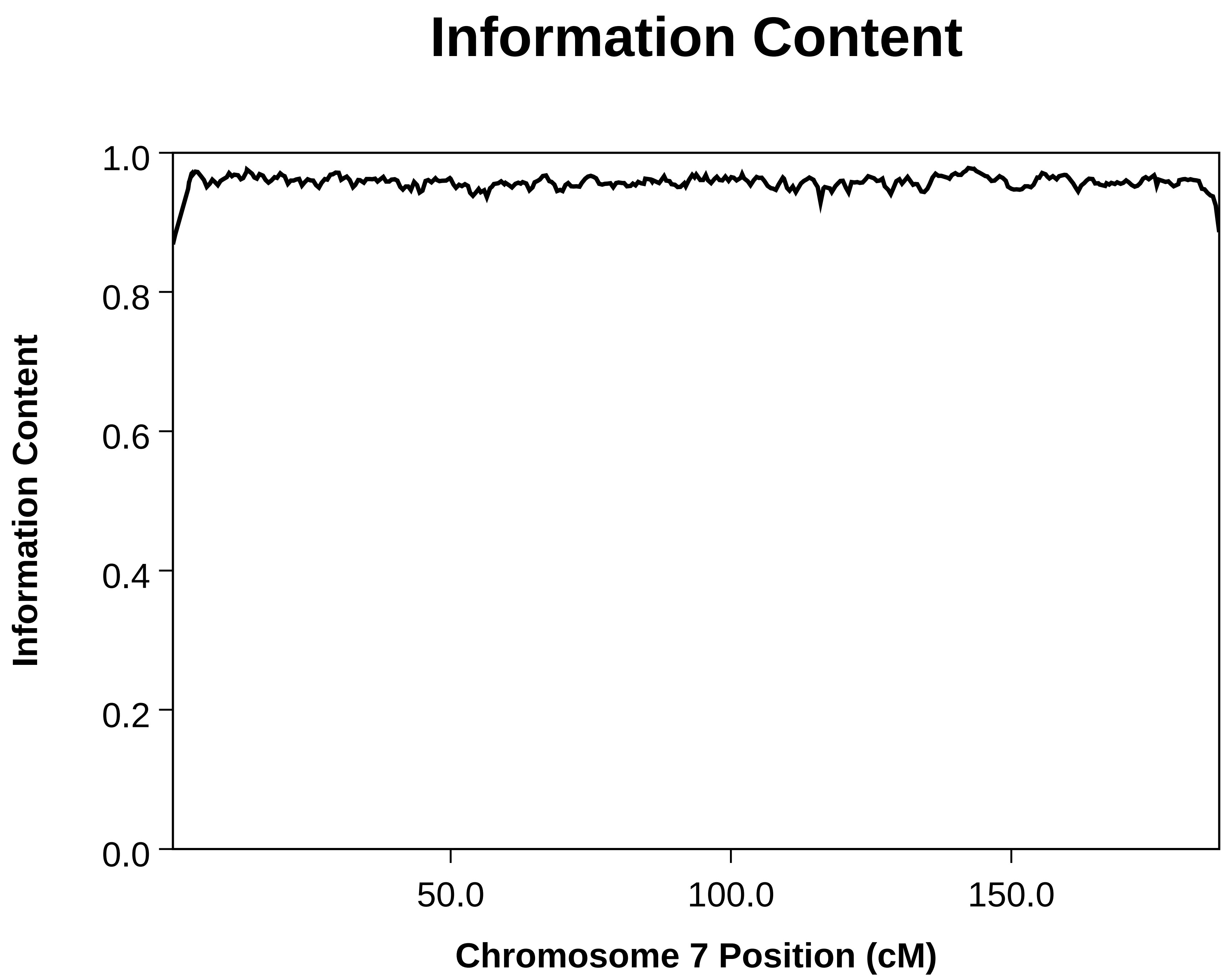
Information Content
1.0
0.8
0.6
0.4
0.2
0.0
Information Content
50.0
100.0
150.0
Chromosome 7 Position (cM)

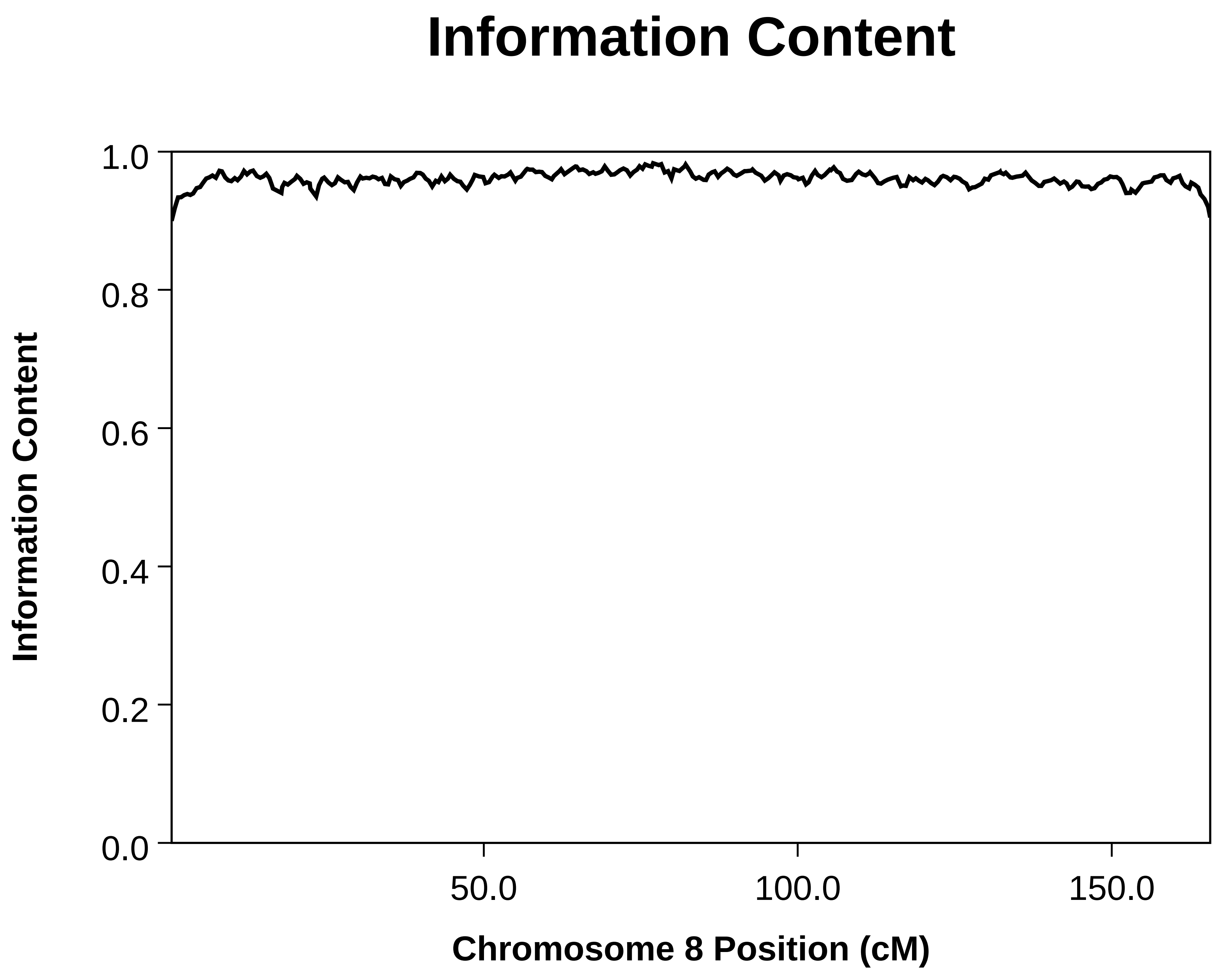
Information Content
1.0
0.8
0.6
0.4
0.2
0.0
50.0
100.0
150.0
Information Content
Chromosome 8 Position (cM)

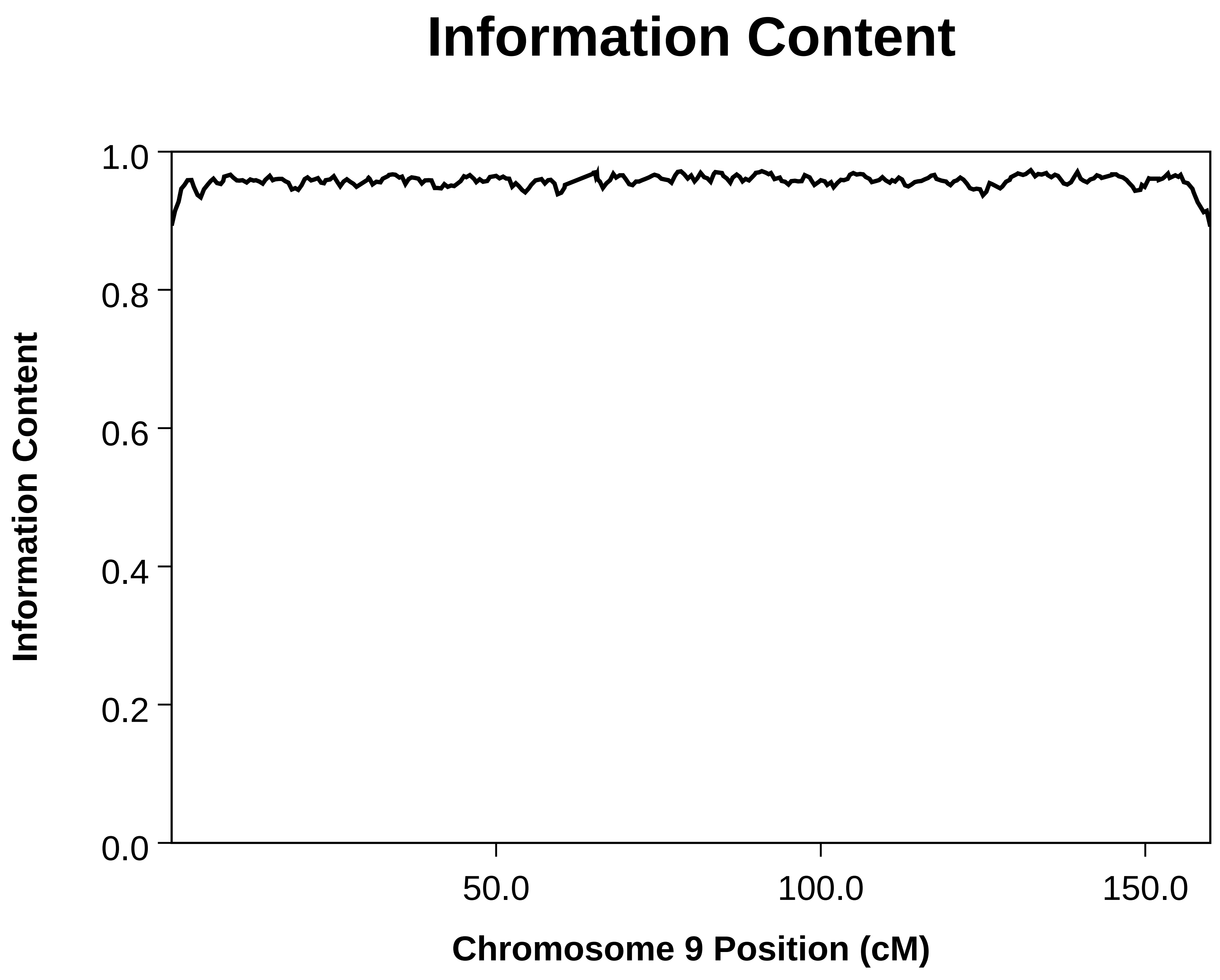
Information Content
1.0
0.8
0.6
0.4
0.2
0.0
Information Content
50.0
100.0
150.0
Chromosome 9 Position (cM)

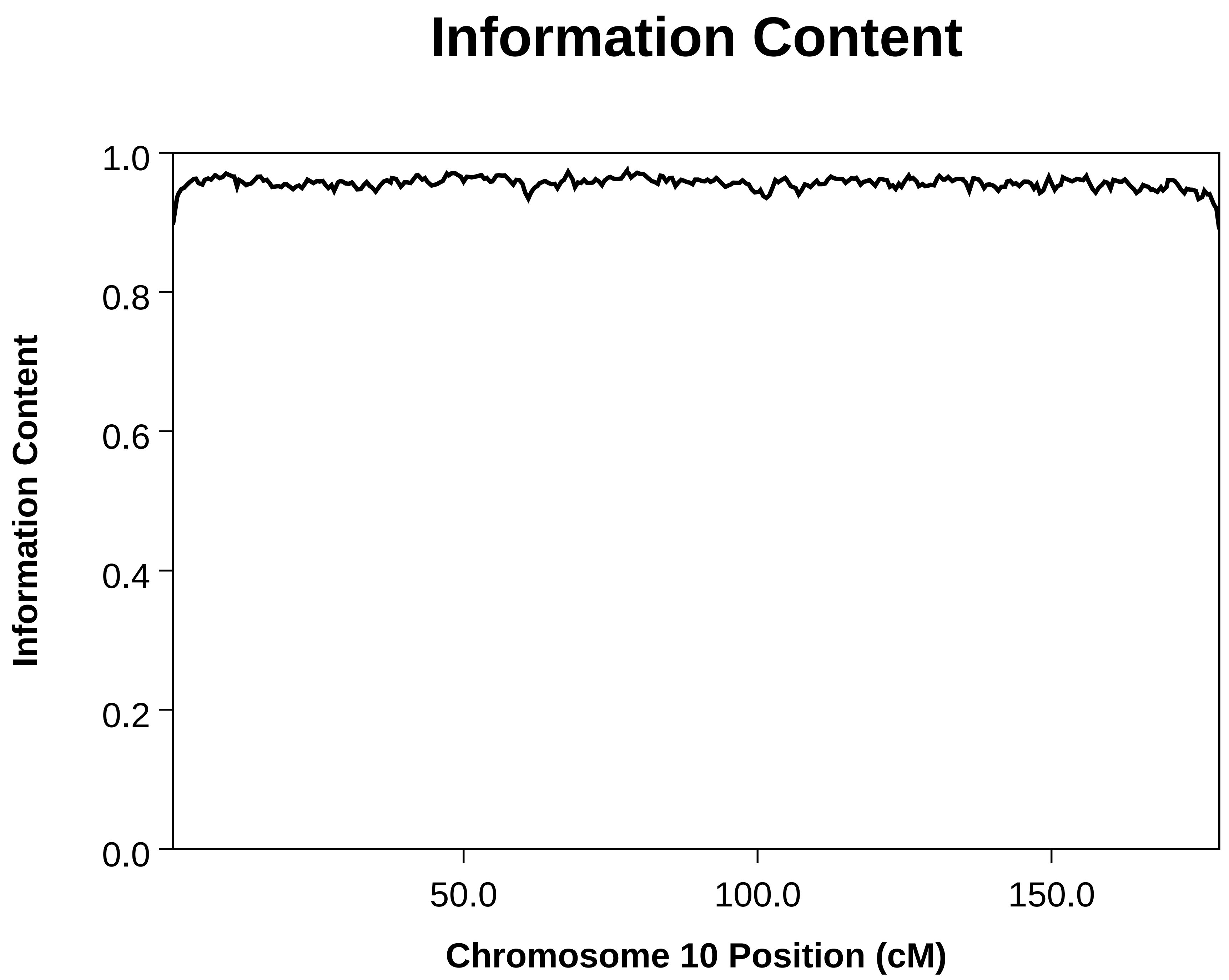

Information Content
1.0
0.8
0.6
0.4
0.2
0.0
Information Content
50.0
100.0
150.0
Chromosome 10 Position (cM)

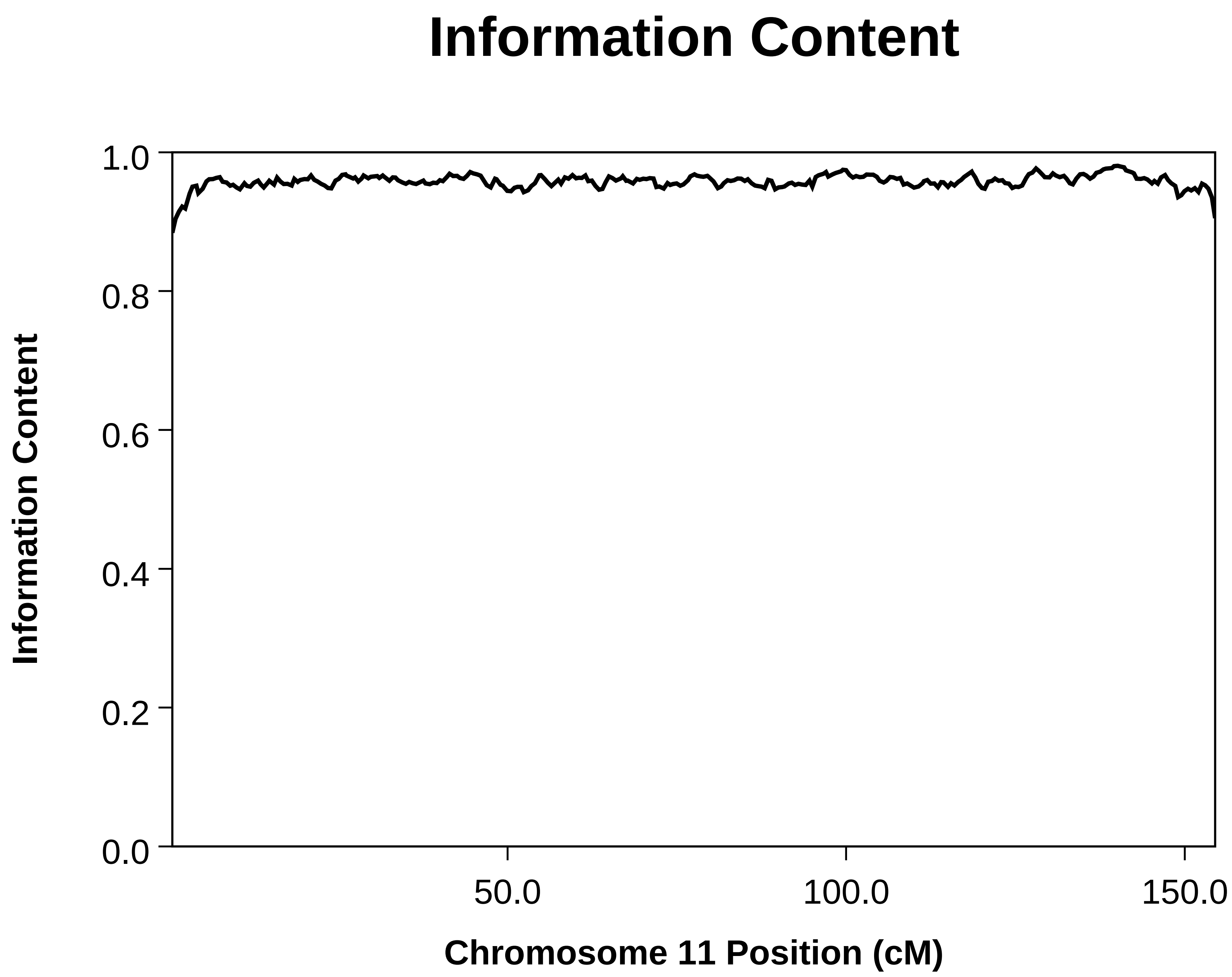
Information Content
1.0
0.8
0.6
0.4
0.2
0.0
Information Content
50.0
100.0
150.0
Chromosome 11 Position (cM)

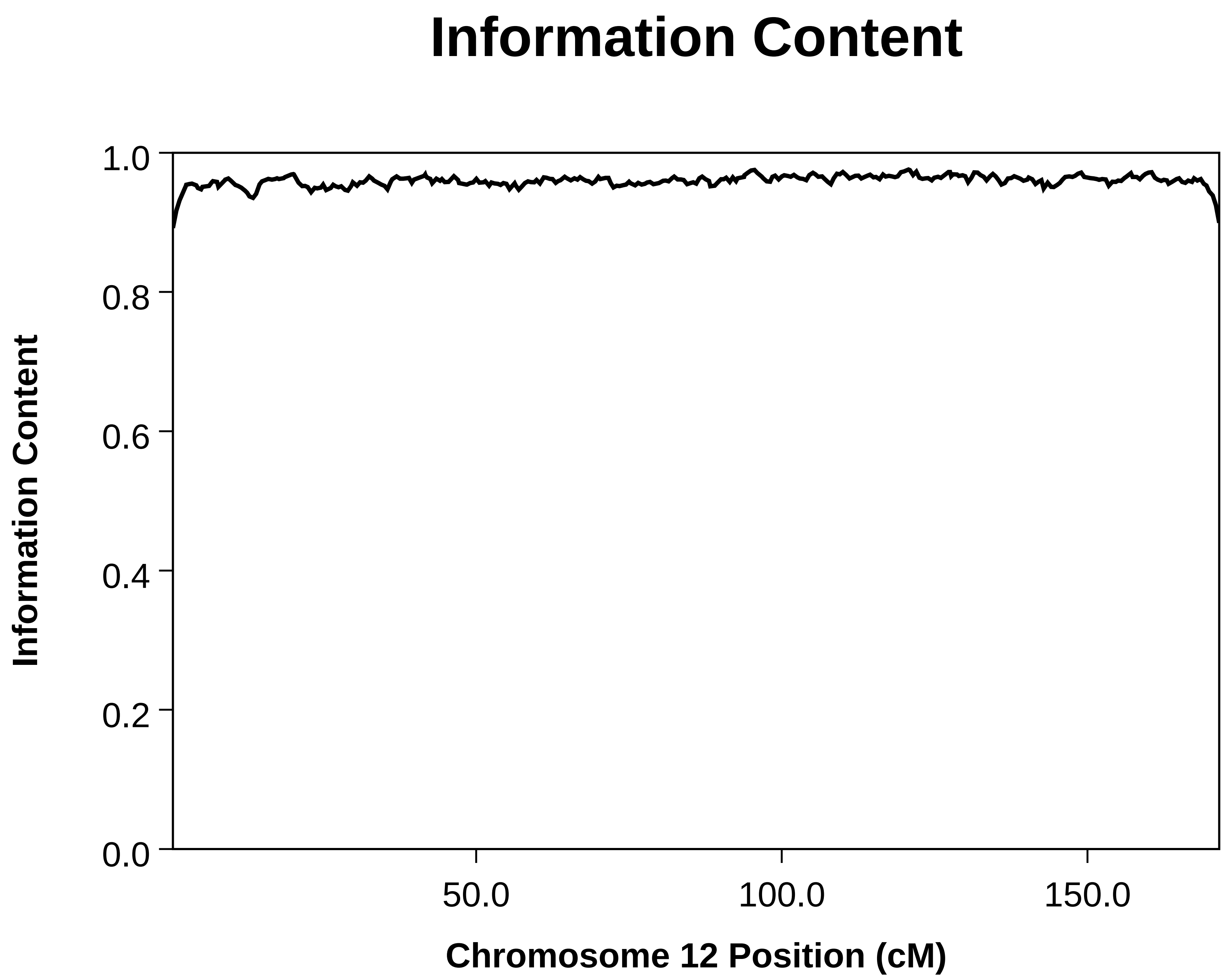

Information Content
1.0
0.8
0.6
0.4
0.2
0.0
50.0
100.0
150.0
Information Content
Chromosome 12 Position (cM)

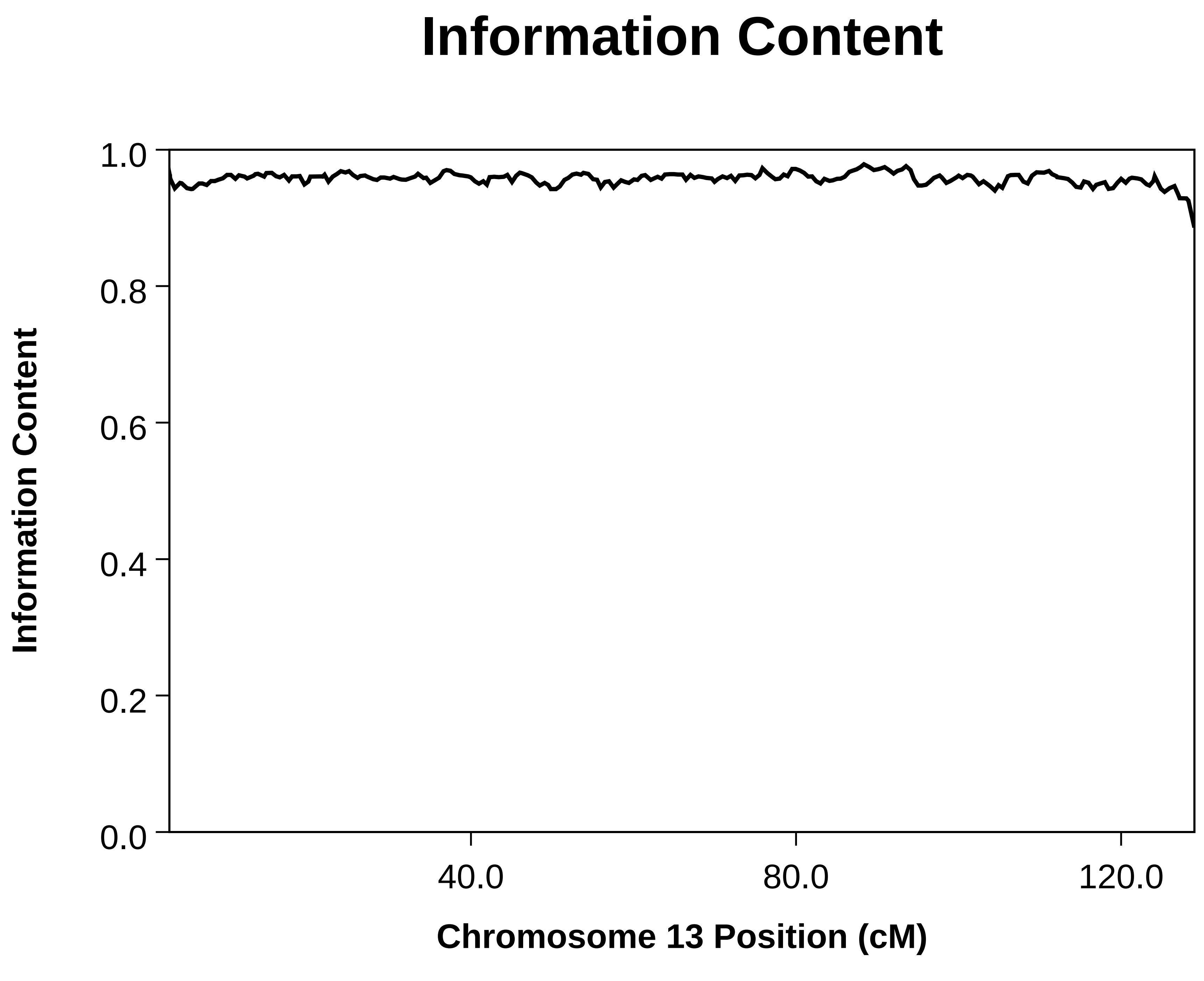

Information Content
1.0
0.8
0.6
0.4
0.2
0.0
Information Content
40.0
80.0
120.0
Chromosome 13 Position (cM)

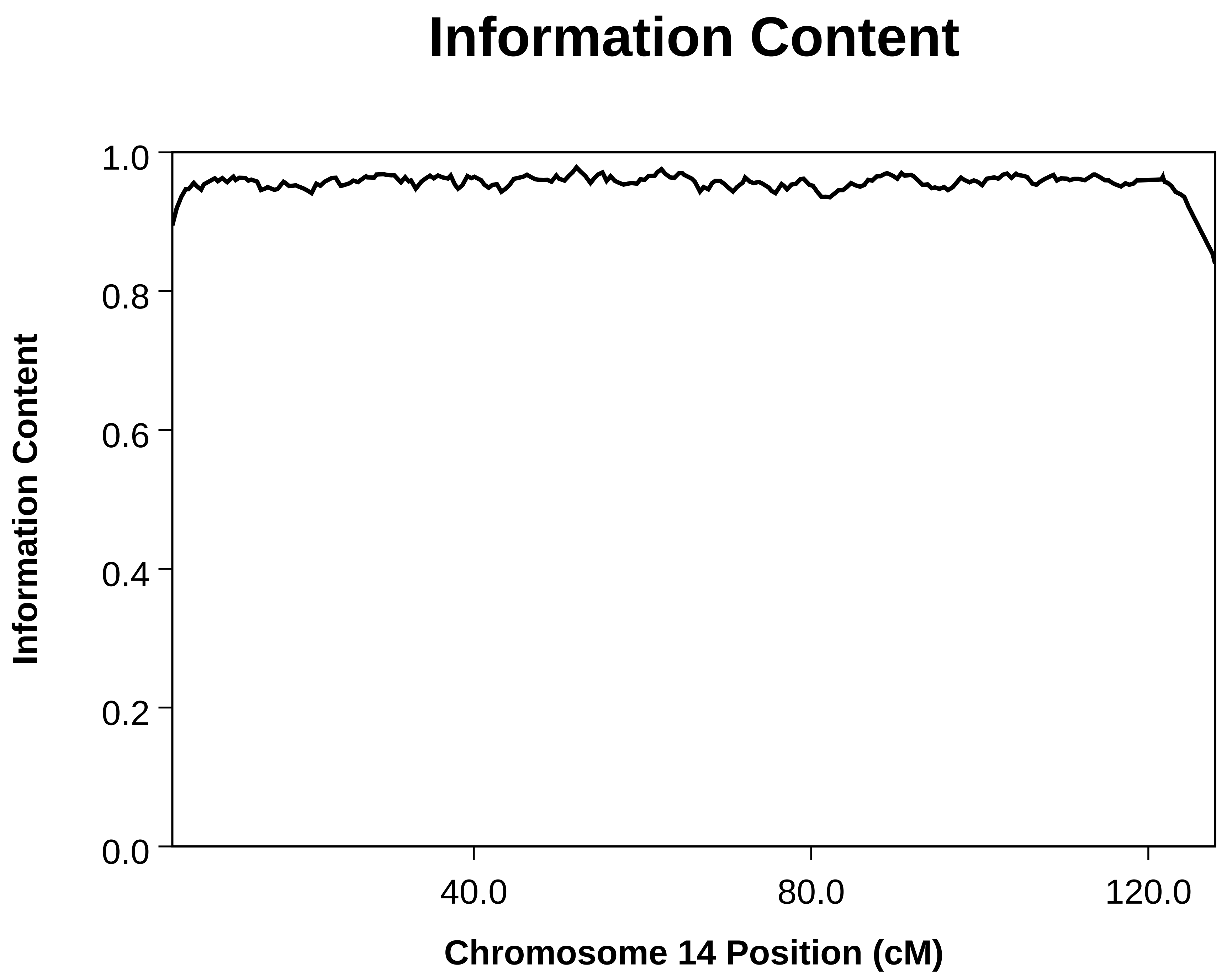
Information Content
1.0
0.8
0.6
0.4
0.2
0.0
40.0
80.0
120.0
Information Content
Chromosome 14 Position (cM)

Information Content

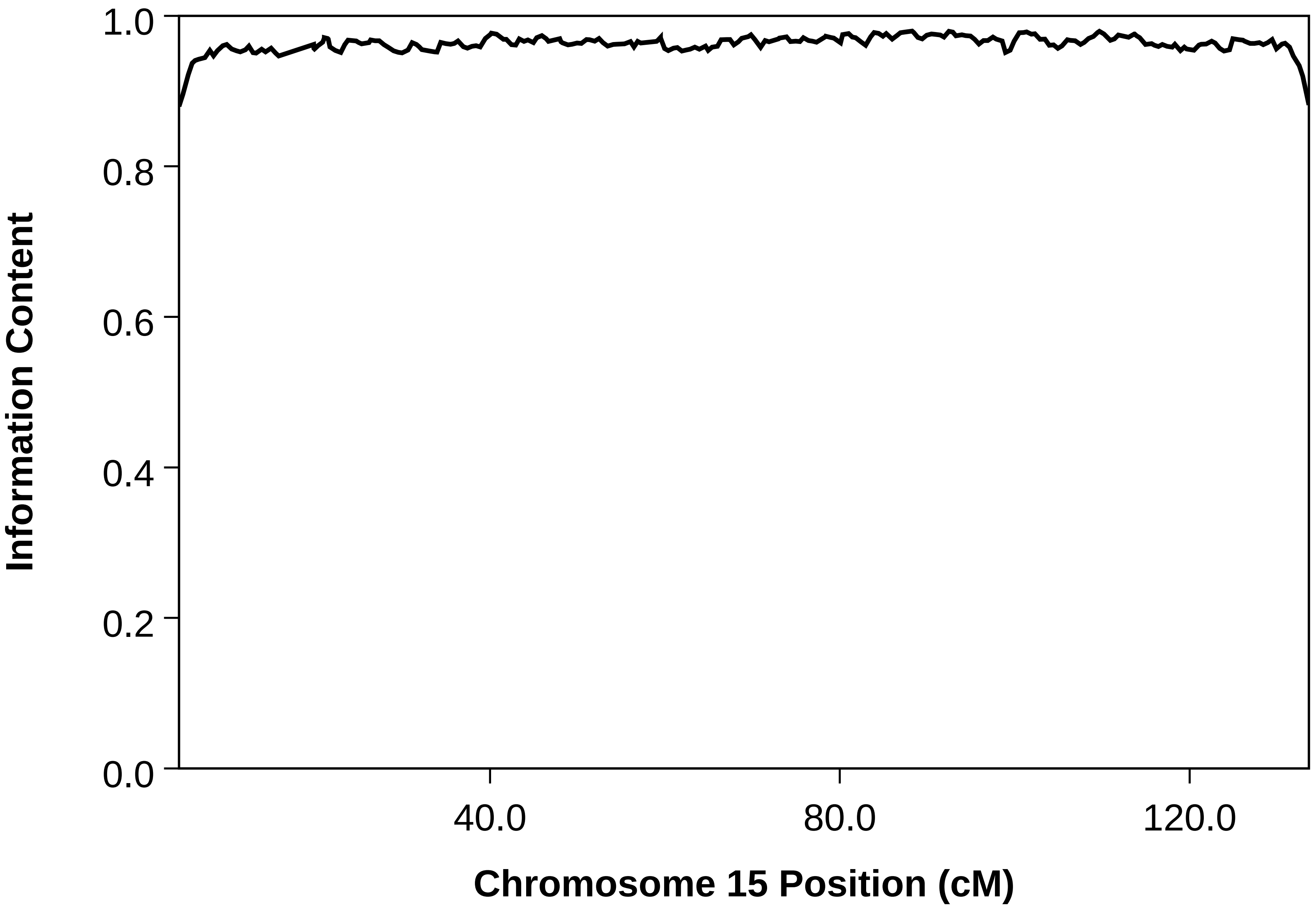

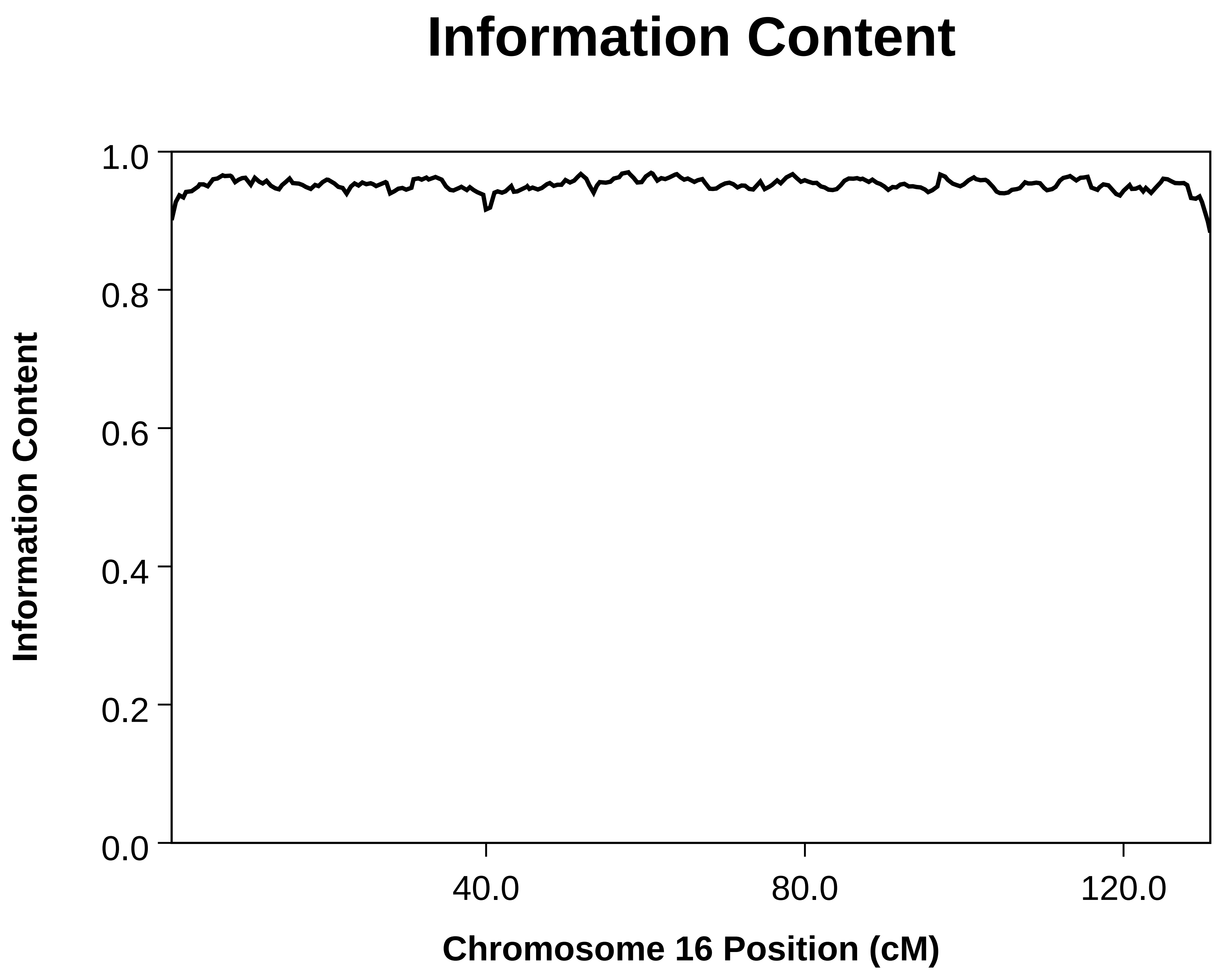
Information Content
Information Content
1.0
0.8
0.6
0.4
0.2
0.0
40.0
80.0
120.0
Chromosome 16 Position (cM)

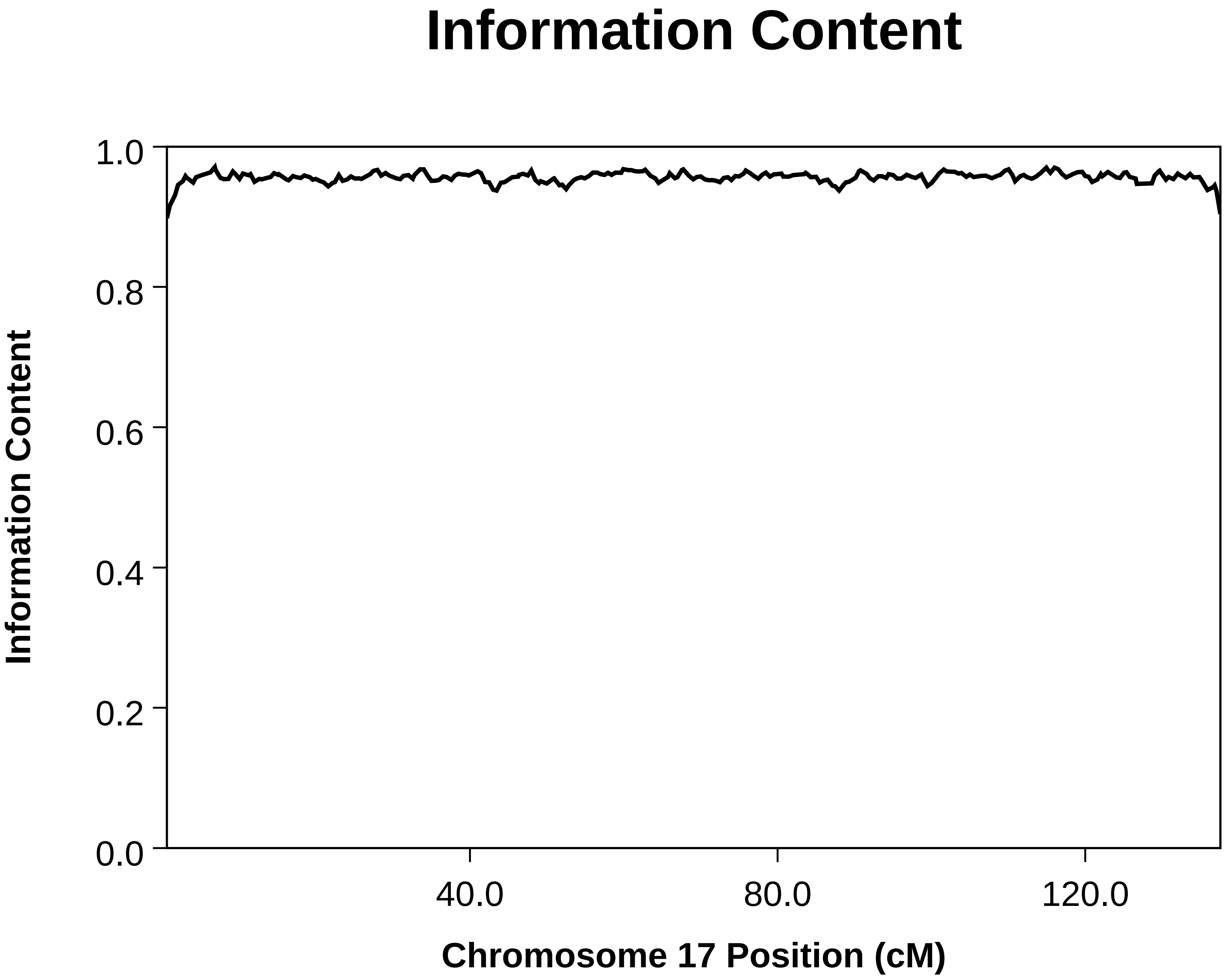

Information Content
1.0
0.8
0.6
0.4
0.2
0.0
Information Content
40.0
80.0
120.0
Chromosome 17 Position (cM)

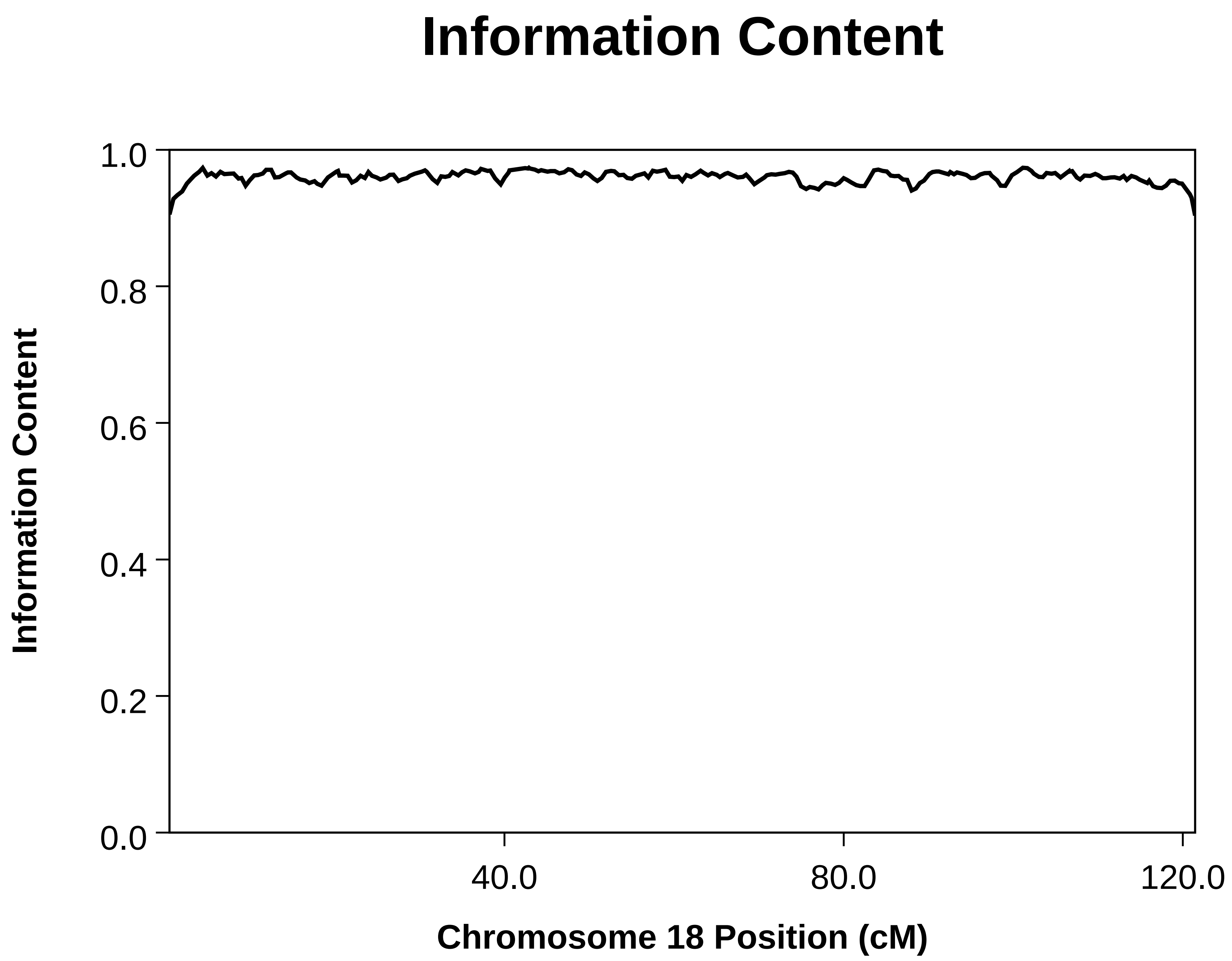
Information Content
1.0
0.8
0.6
0.4
0.2
0.0
40.0
80.0
120.0
Chromosome 18 Position (cM)
Information Content

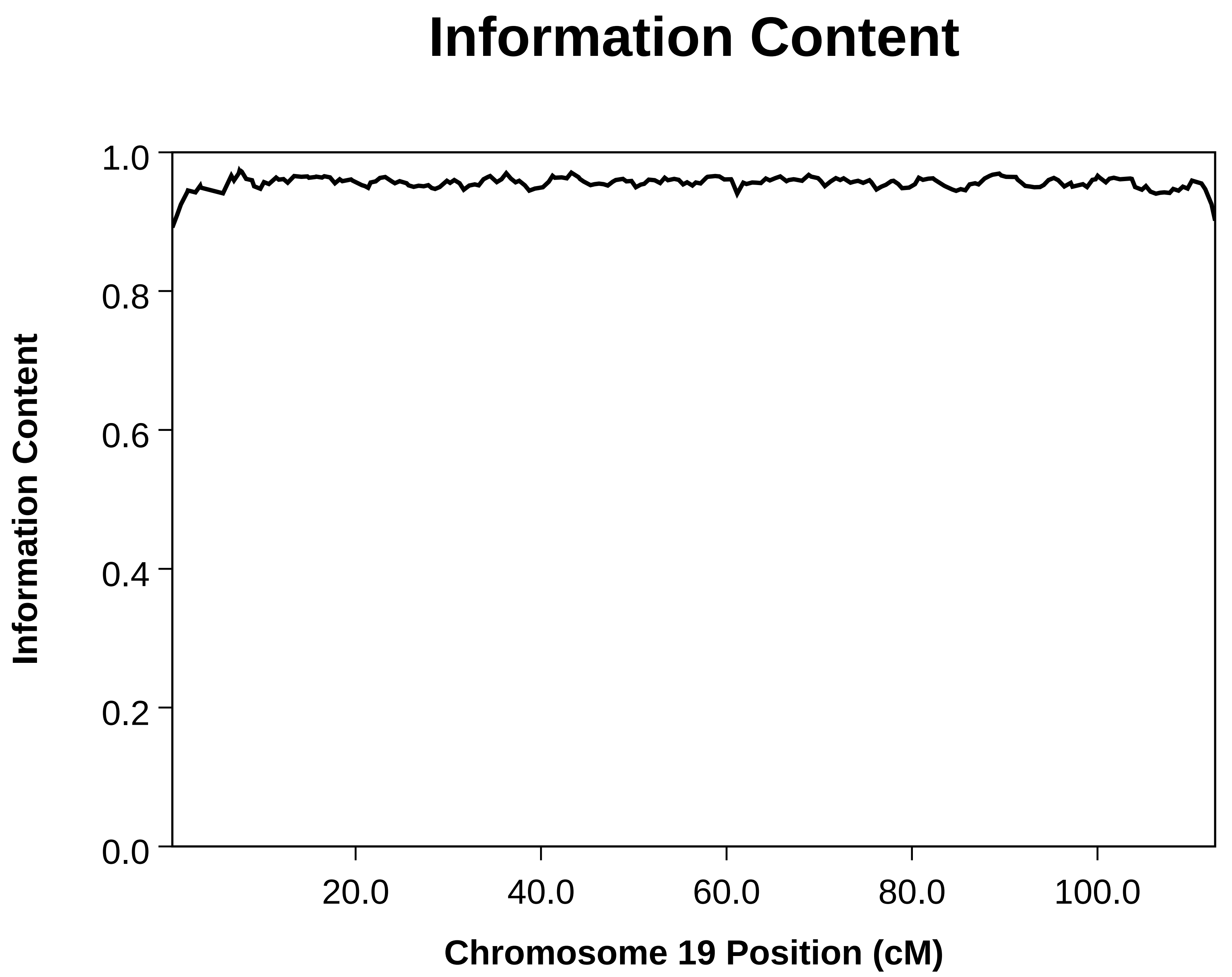
Information Content
Information Content
1.0
0.8
0.6
0.4
0.2
0.0
20.0
40.0
60.0
80.0
100.0
Chromosome 19 Position (cM)

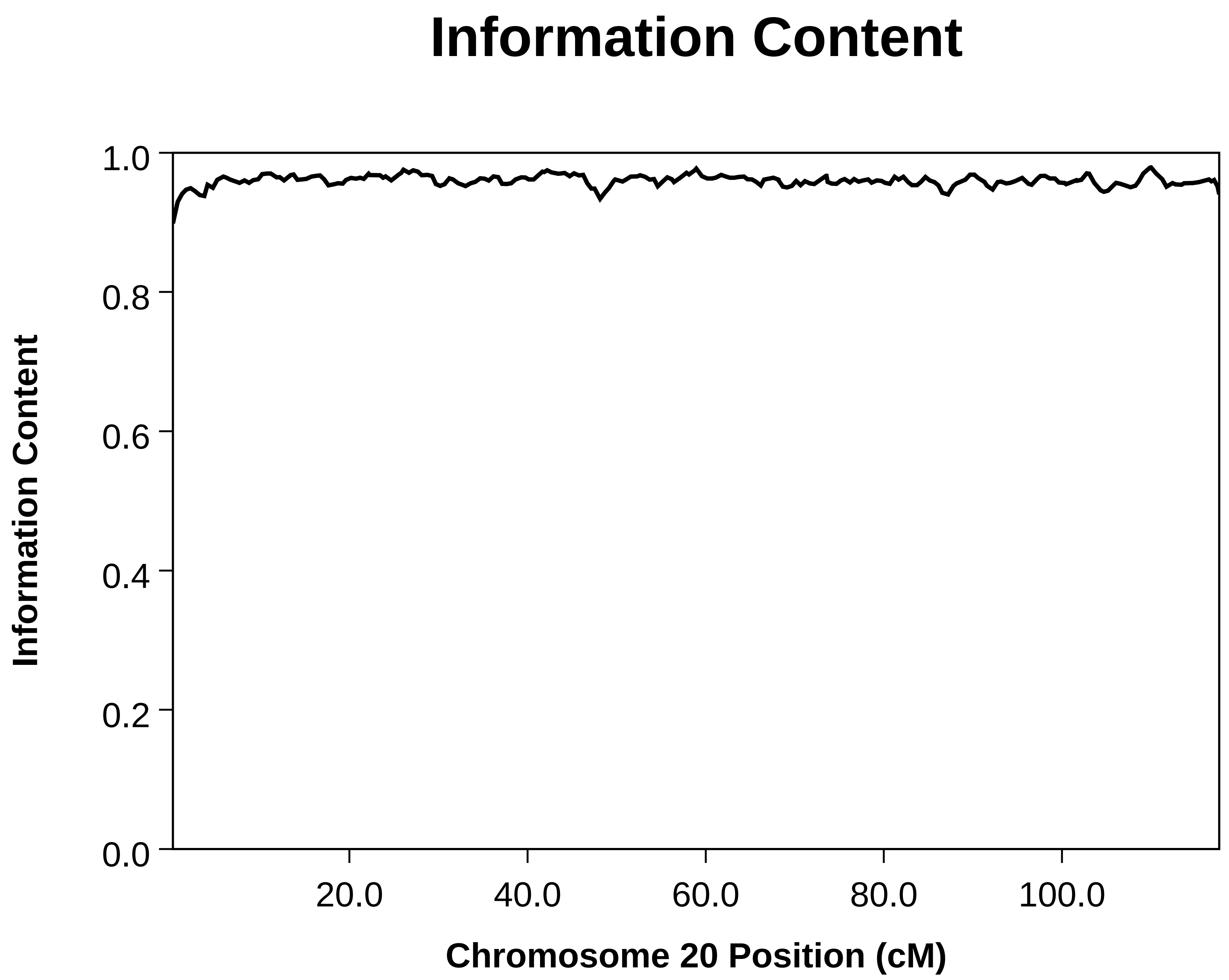
Information Content
1.0
0.8
0.6
0.4
0.2
0.0
Information Content
20.0
40.0
60.0
80.0
100.0
Chromosome 20 Position (cM)

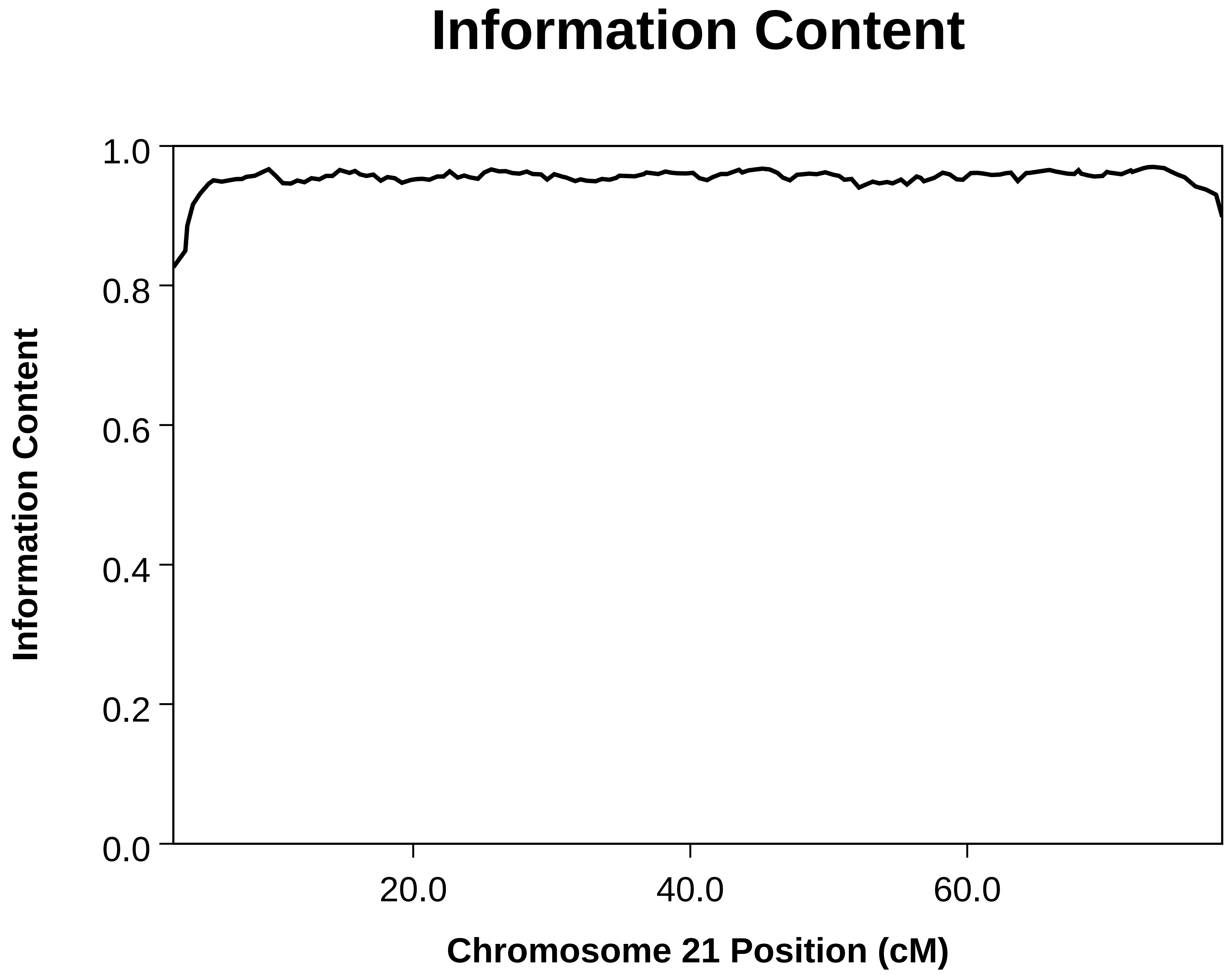
Information Content
1.0
0.8
0.6
0.4
0.2
0.0
Information Content
20.0
40.0
60.0
Chromosome 21 Position (cM)

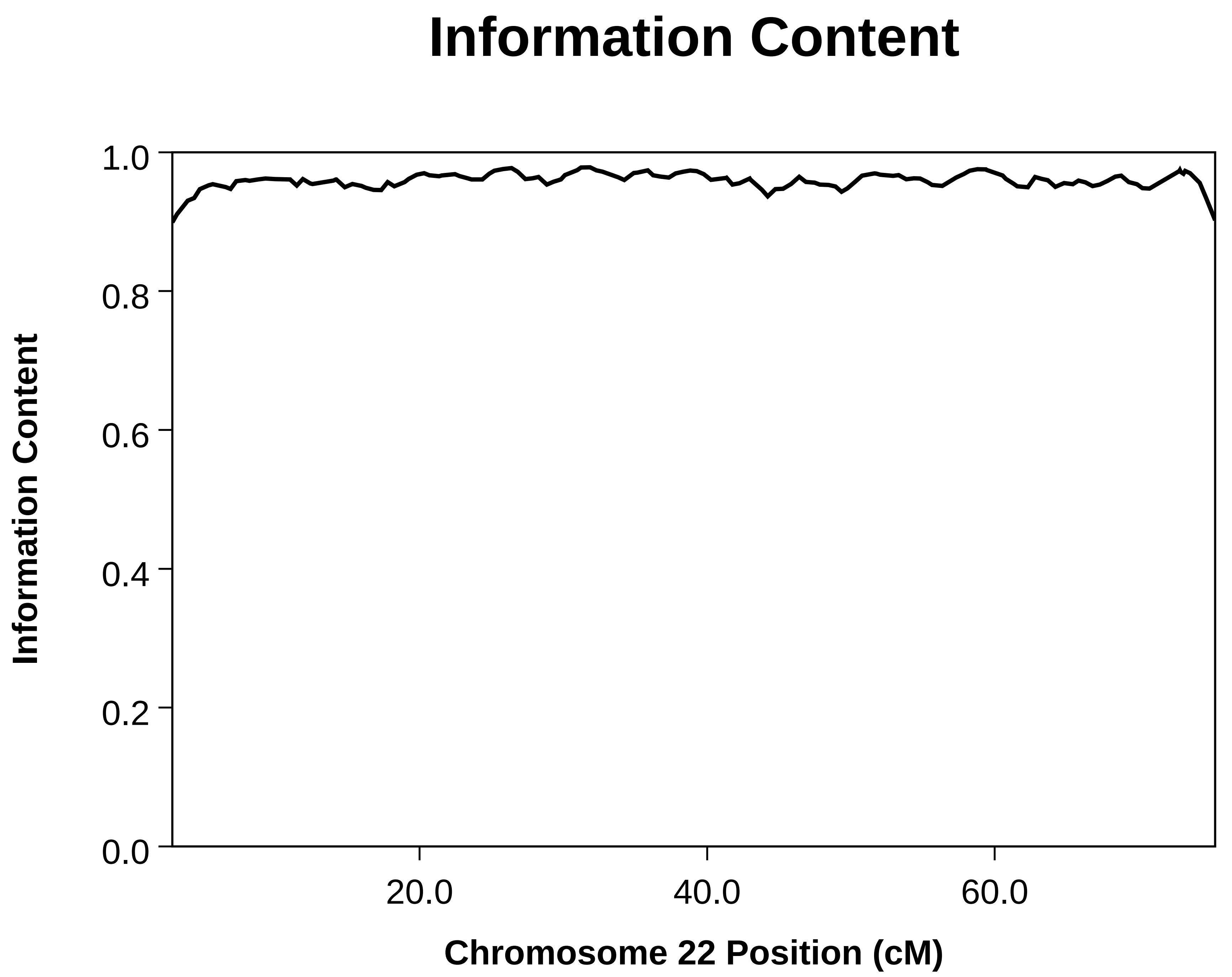
Information Content
1.0
0.8
0.6
0.4
0.2
0.0
Information Content
20.0
40.0
60.0
Chromosome 22 Position (cM)
