# Supplementary figures and images for: A new genome scan for primary nonsyndromic vesicoureteric reflux emphasizes high genetic heterogeneity and shows linkage and association with various genes already implicated in urinary tract development
Source: Mol Genet Genomic Med. 2013 Jul 7;2(1):7–29. doi: 10.1002/mgg3.22 (PMC3907909; doi:10.1002/mgg3.22)

# Information Content

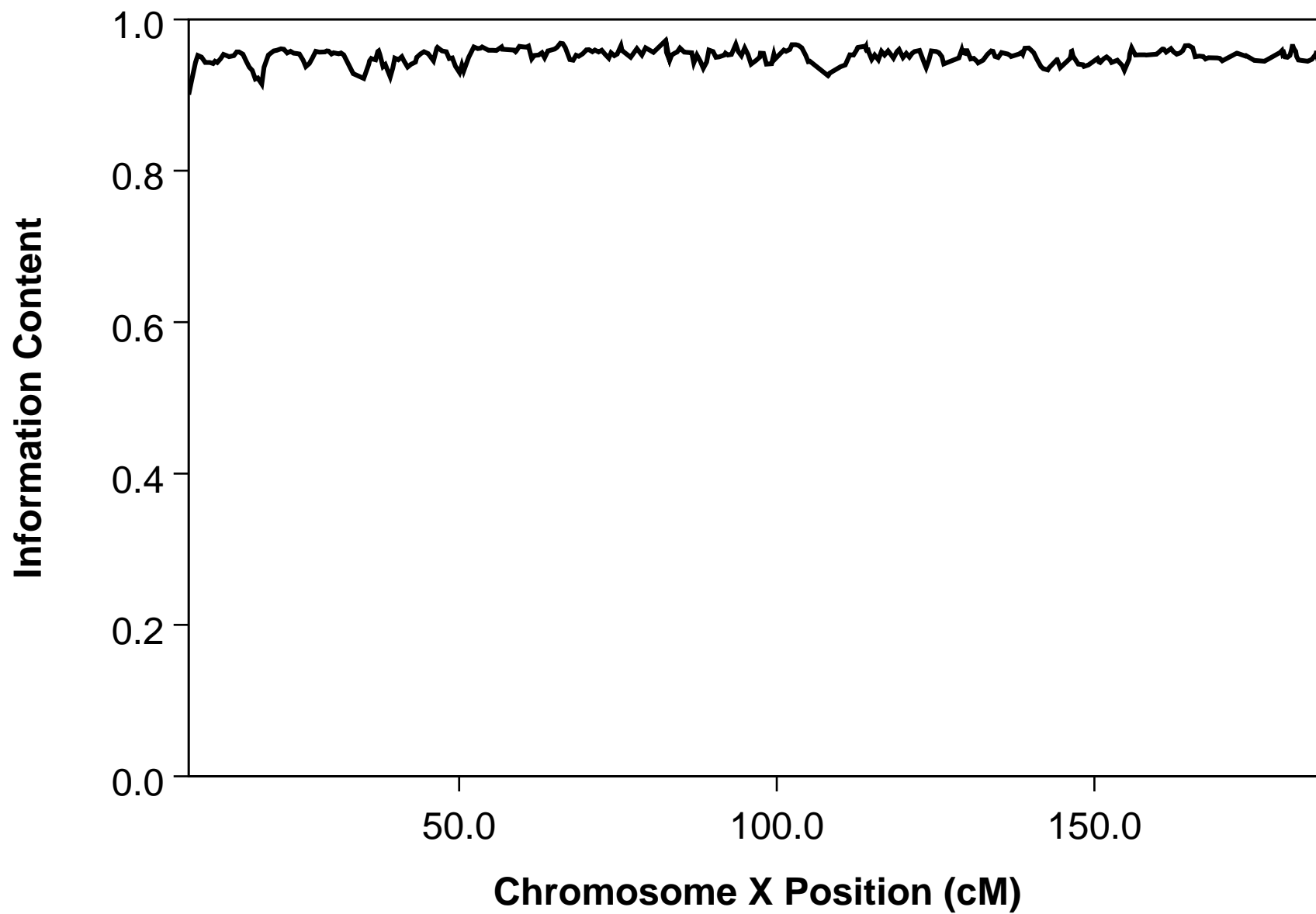

Supplement: Figure S2 — Information content across the X chromosome by the SNPs used for linkage. [file mgg30002-0007-sd2.pdf]

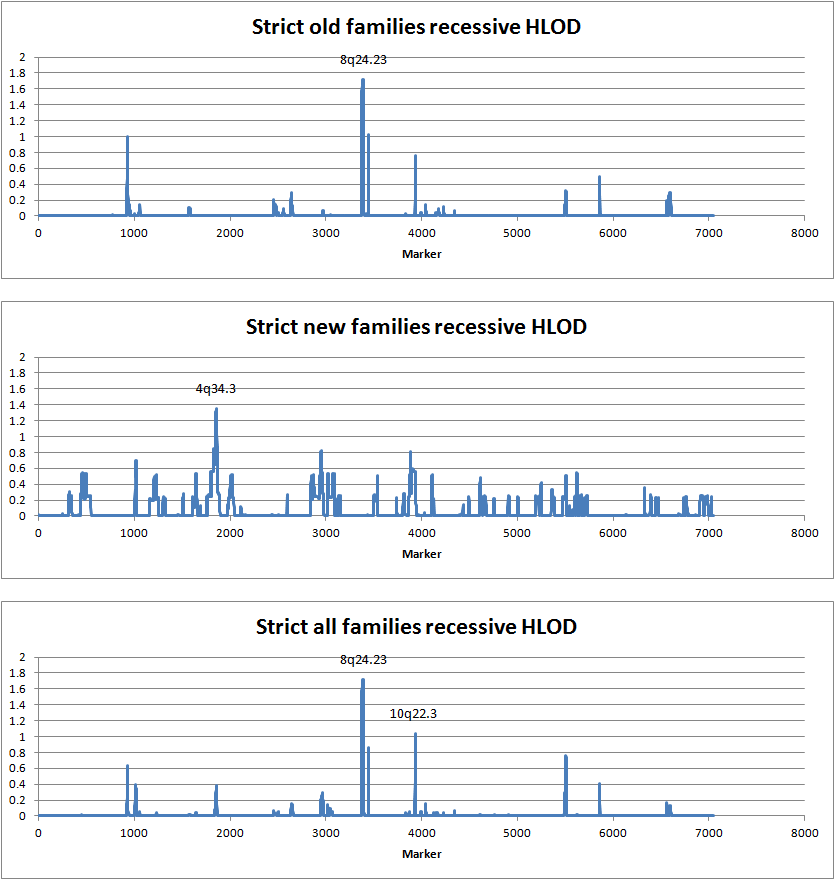


**Supplementary Figure S4**. HLOD analysis with a recessive model of inheritance.

Supplement: Figure S4 — HLOD analysis with a recessive model of inheritance. [file mgg30002-0007-sd4.doc]

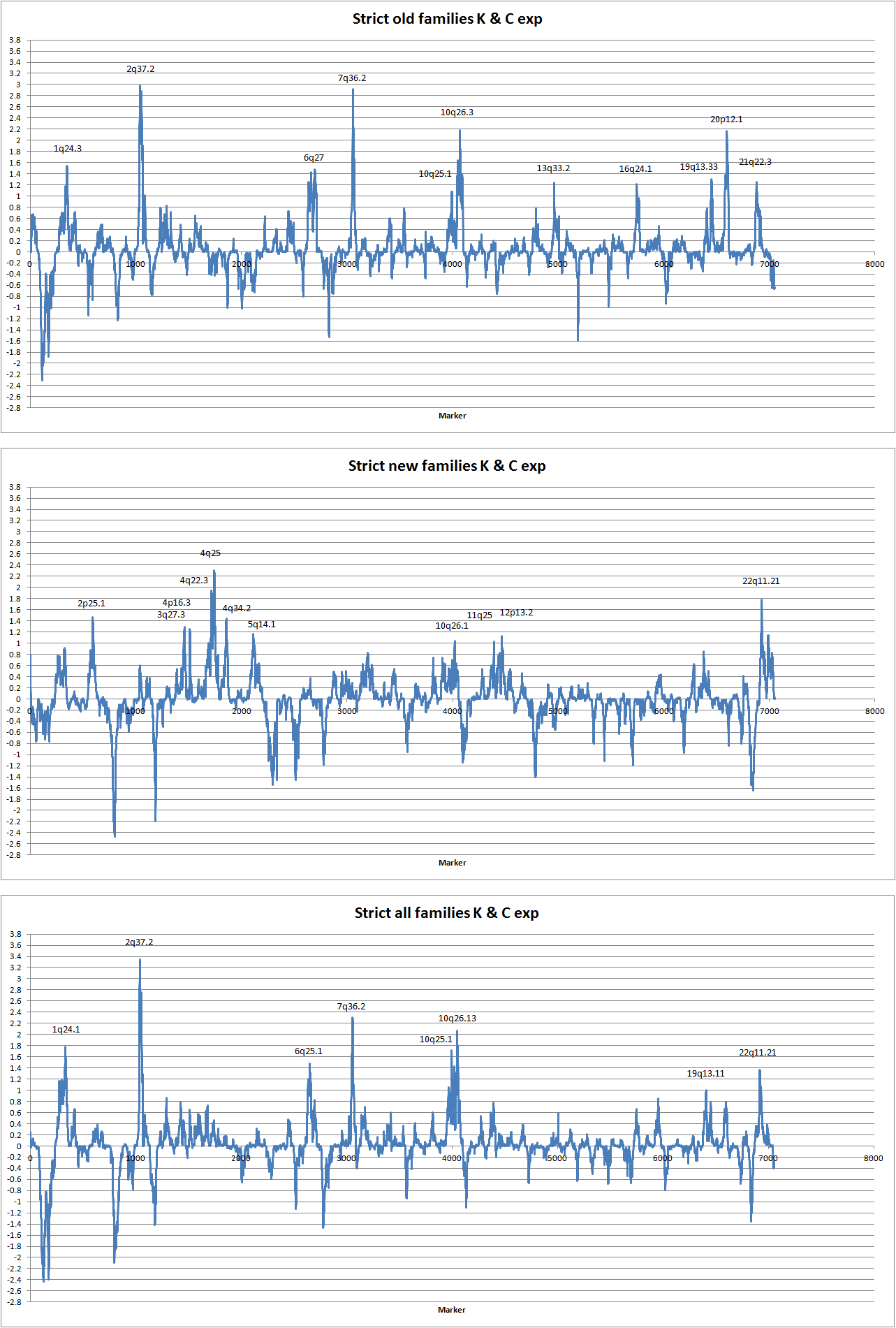


**Supplementary Figure S5**. Nonparametric analyses for the old, new and all families.

Supplement: Figure S5 — Nonparametric analyses for the old, new, and all families. [file mgg30002-0007-sd5.doc]

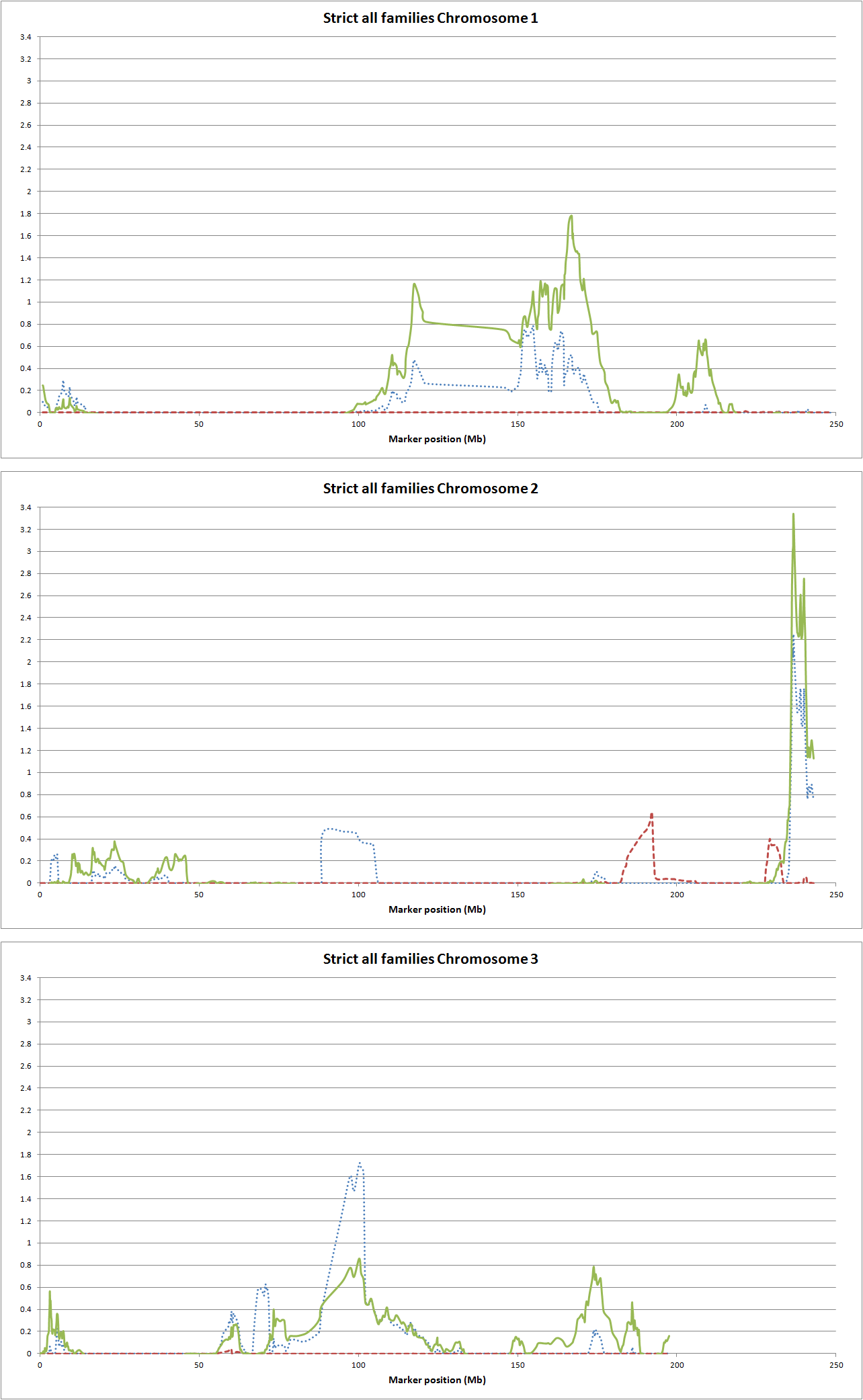


**Supplementary Figure S6.** Linkage in all families

······ HLOD dominant; ----- HLOD recessive; ~~-----~~ ZLRLOD.


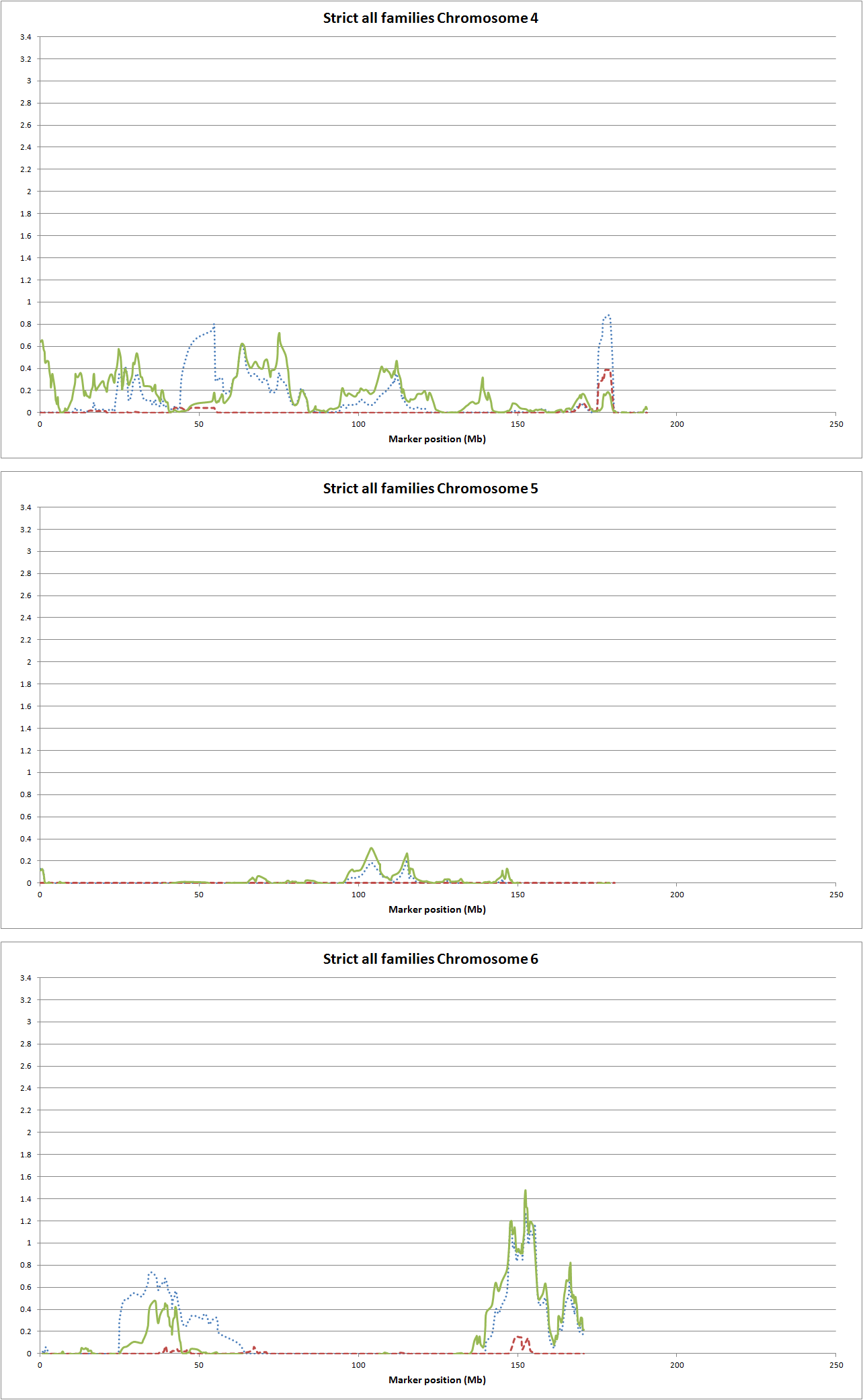


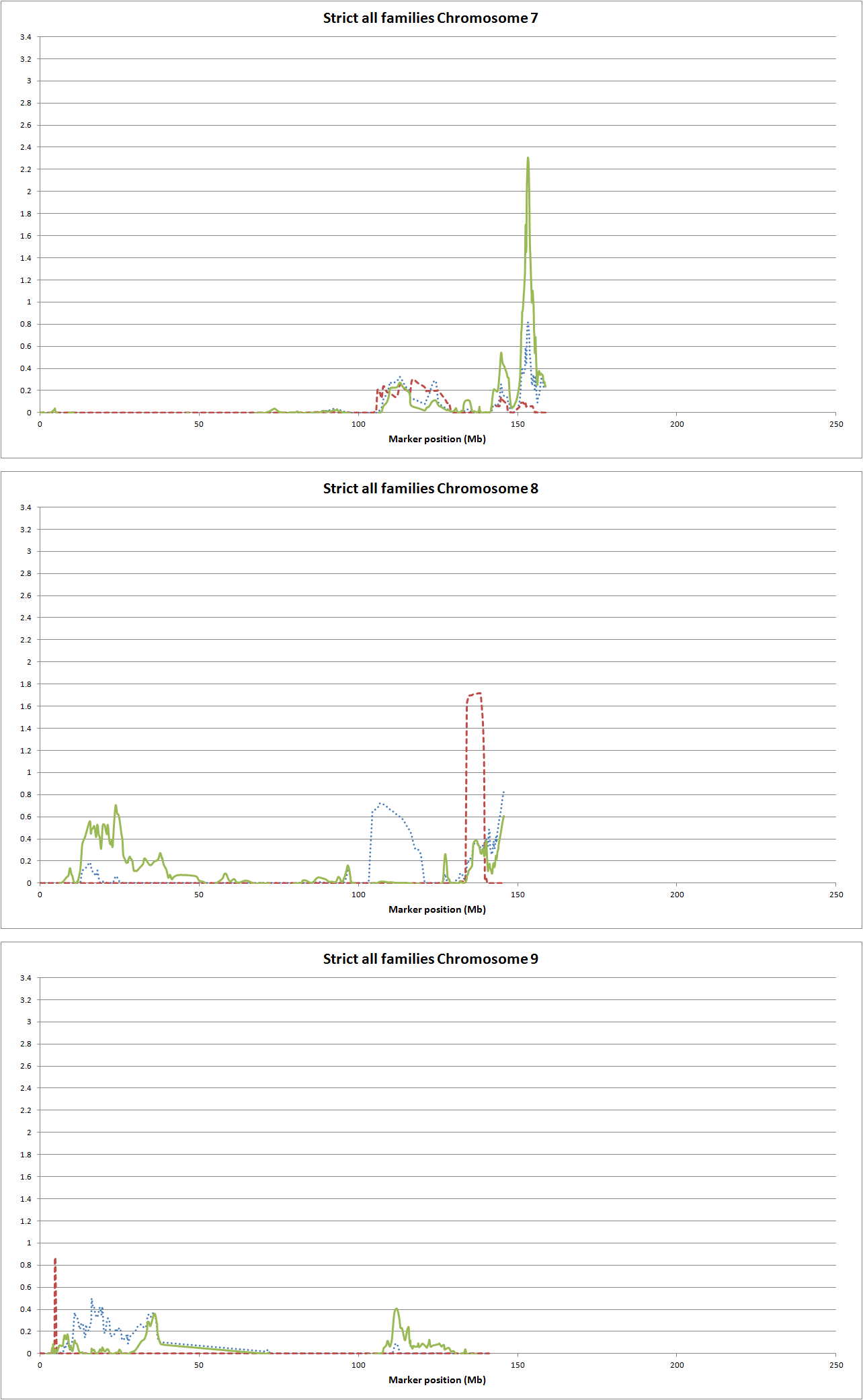


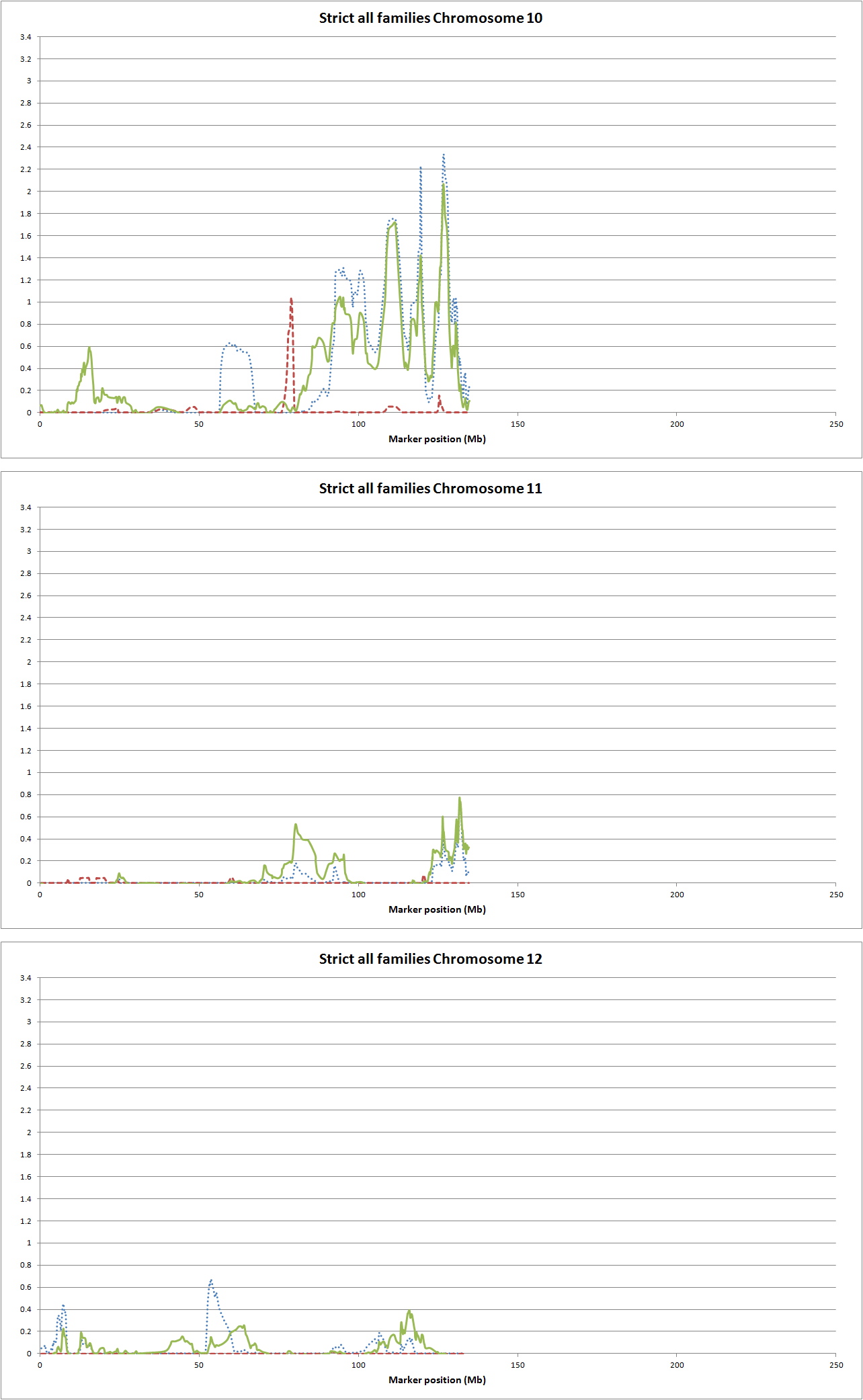


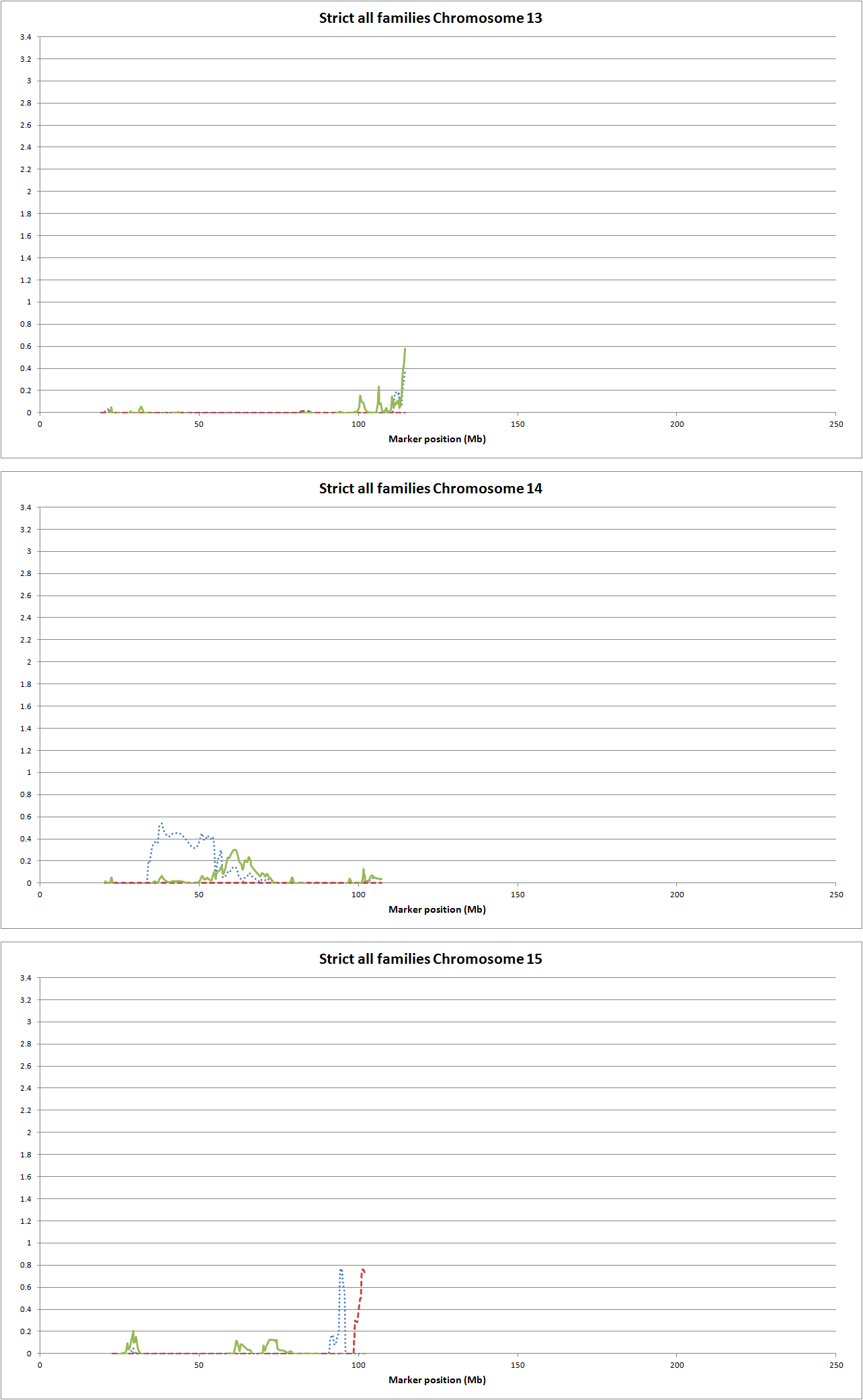


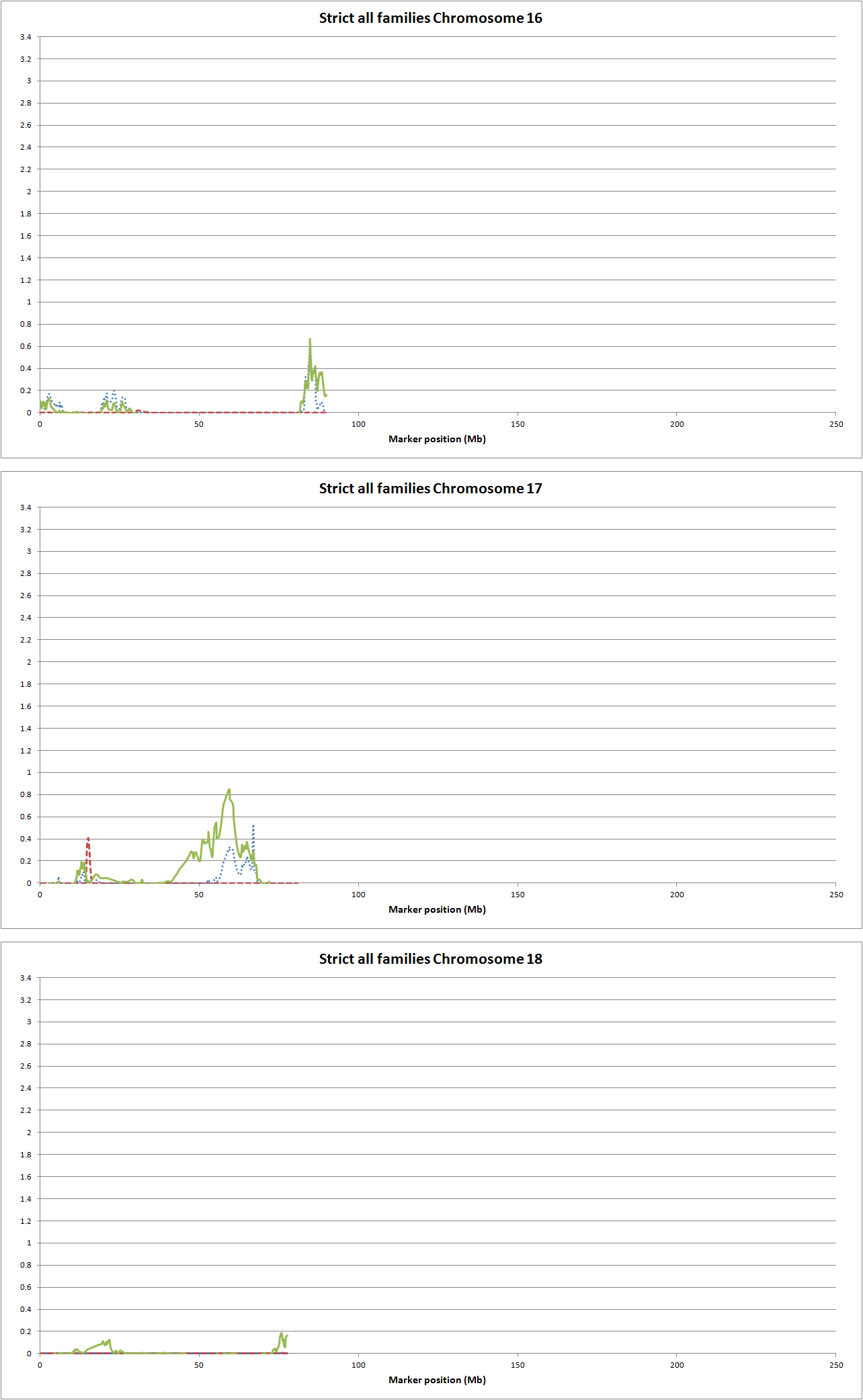


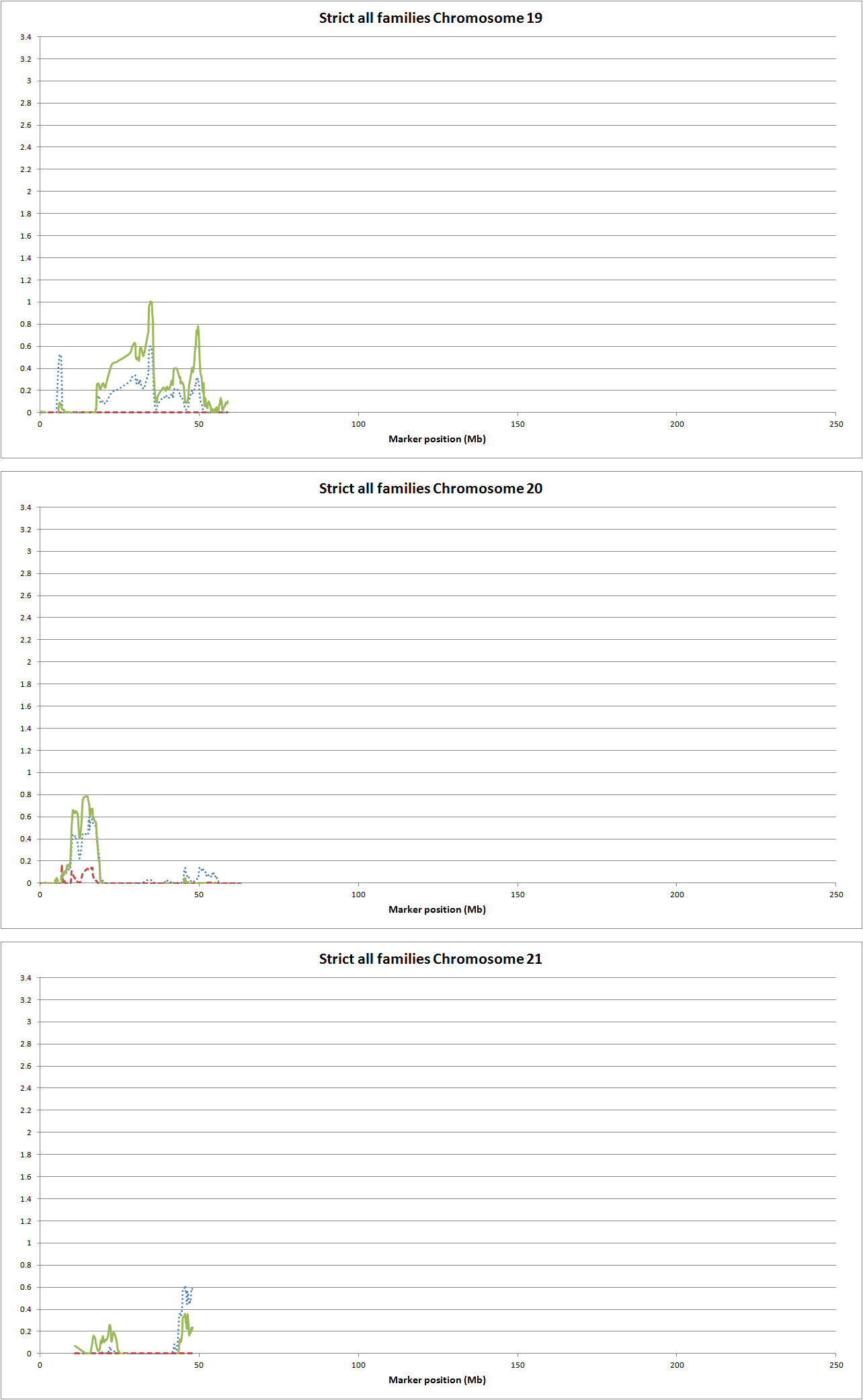


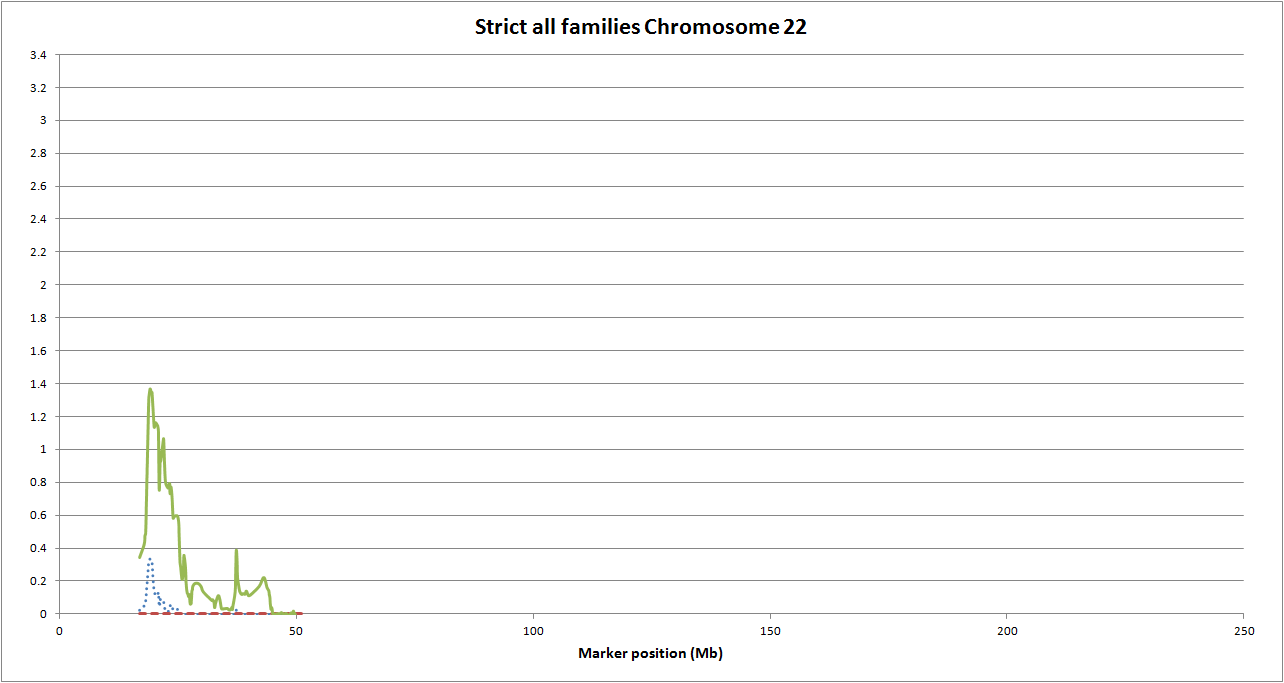

Supplement: Figure S6 — Linkage in all families ______ HLOD dominant; ------- HLOD recessive; ——ZLRLOD. [file mgg30002-0007-sd6.doc]

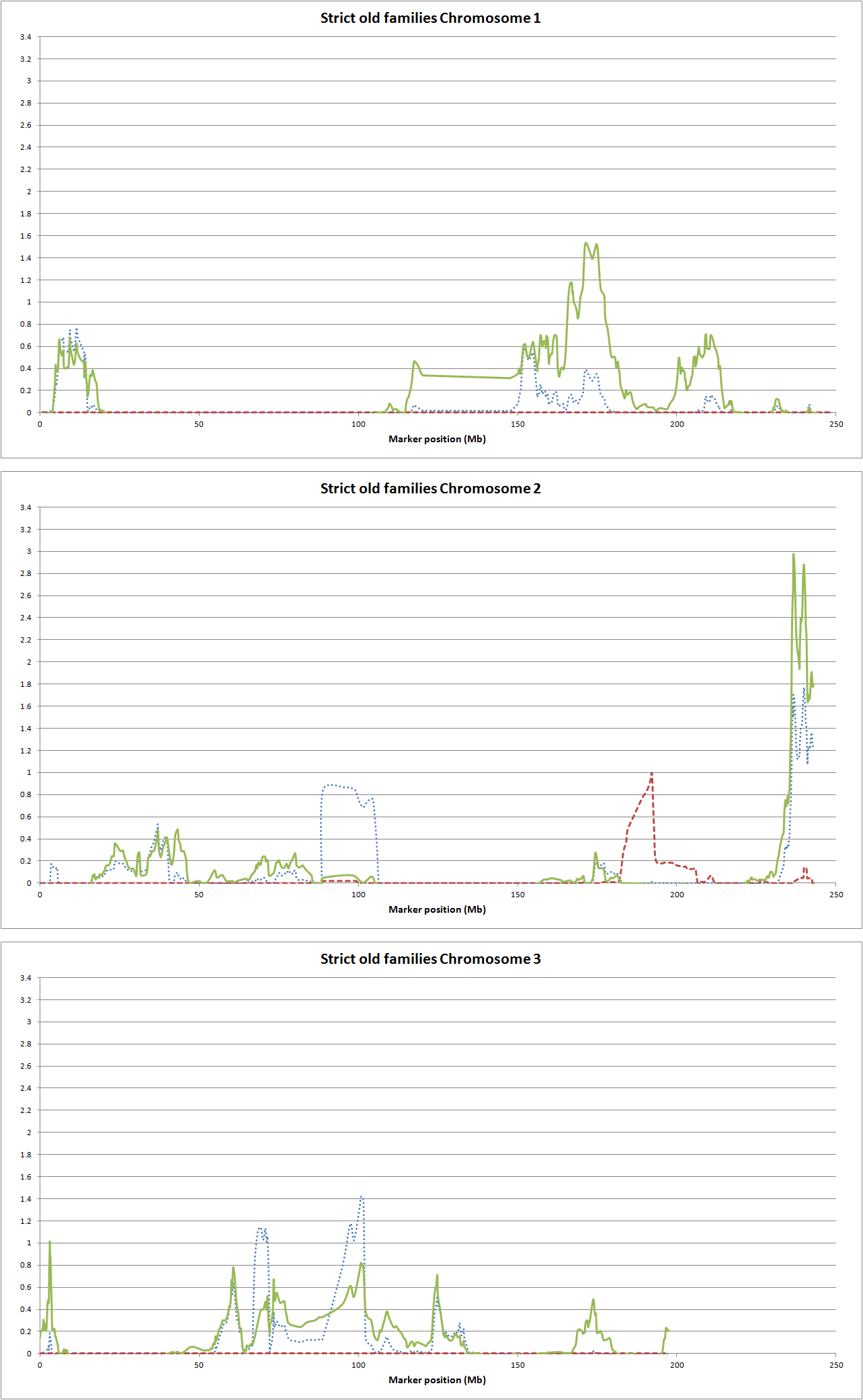


**Supplementary Figure S7.** Linkage in the ‘old’ families

······ HLOD dominant; ----- HLOD recessive; ~~-----~~ ZLRLOD.


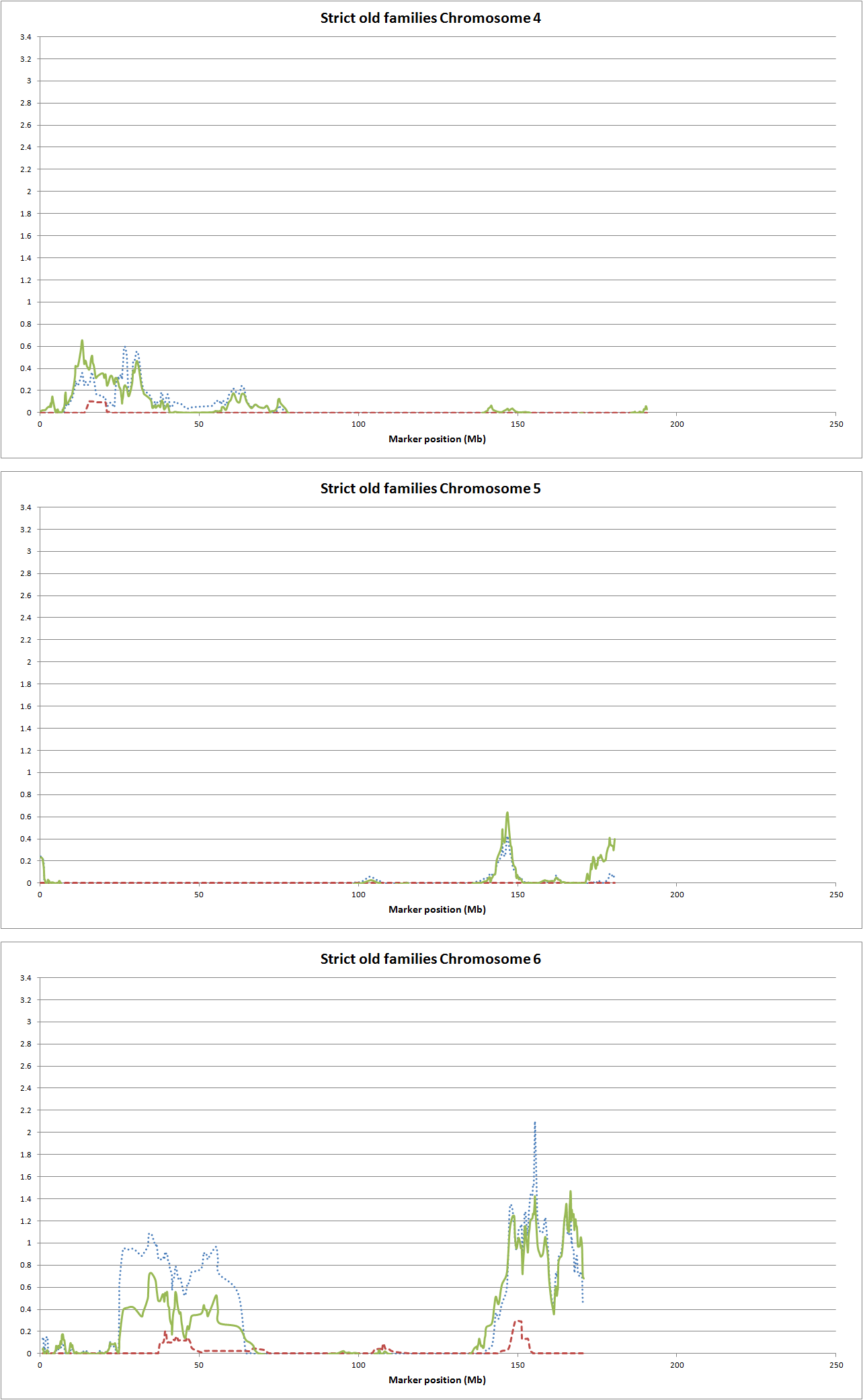


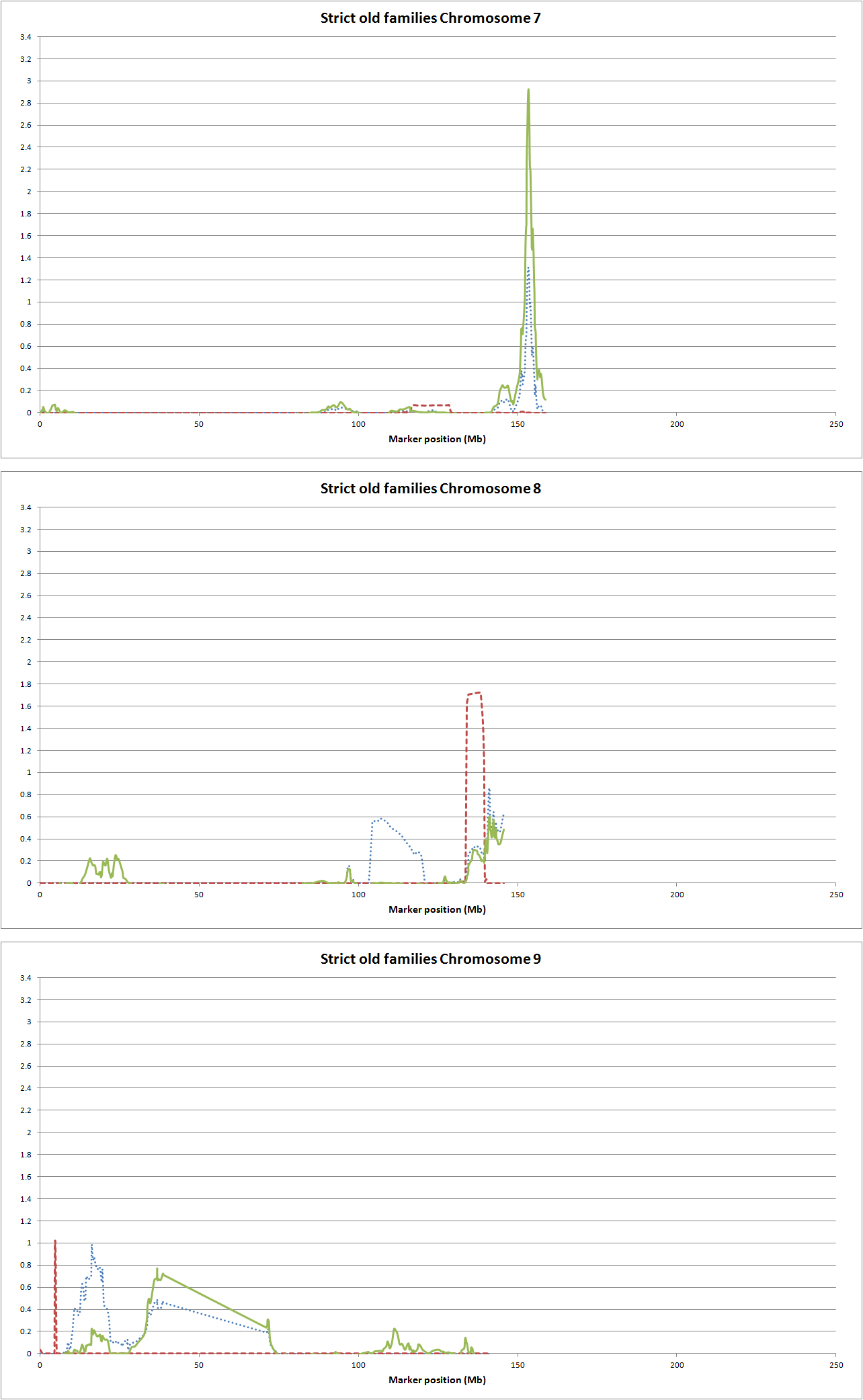


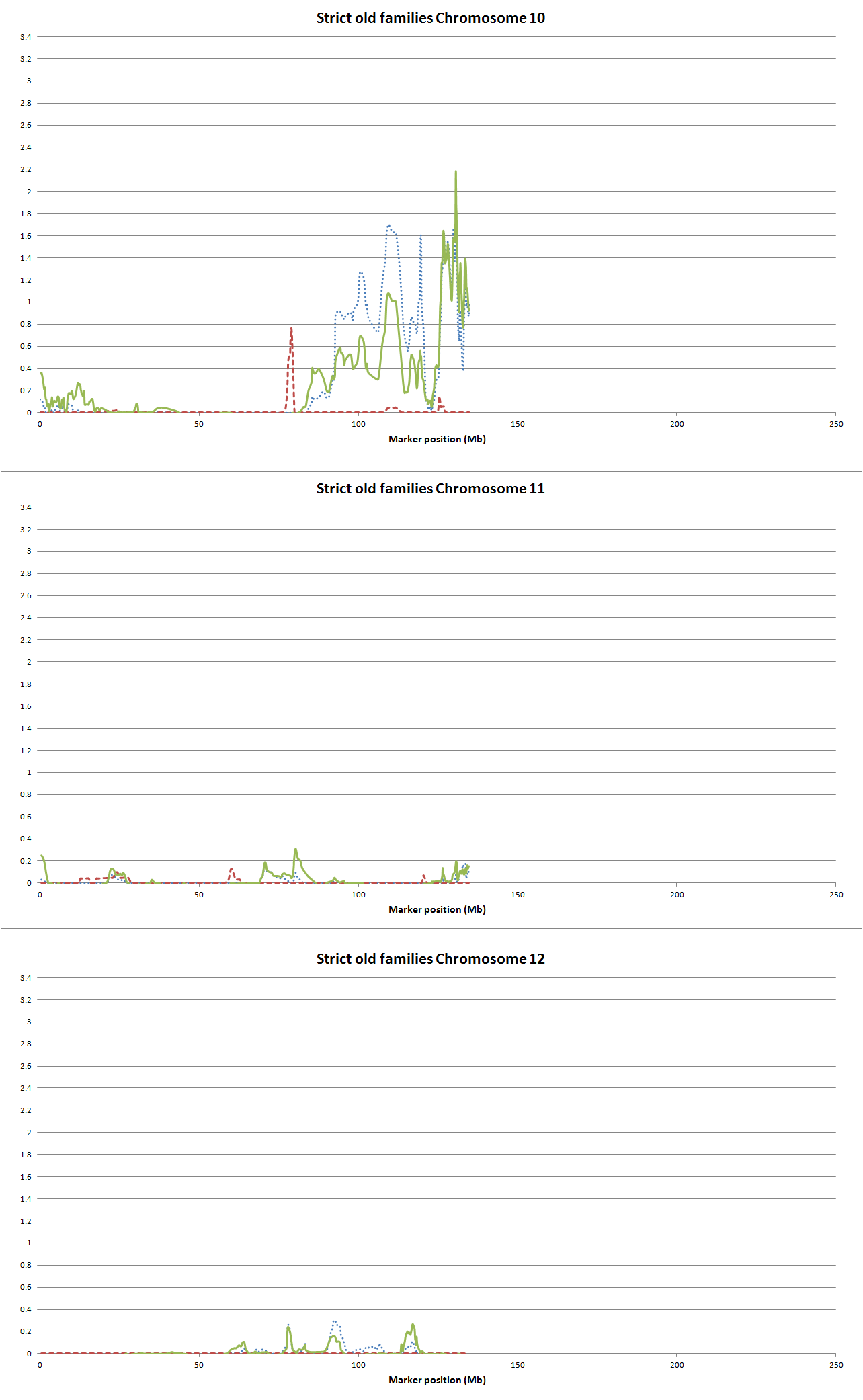


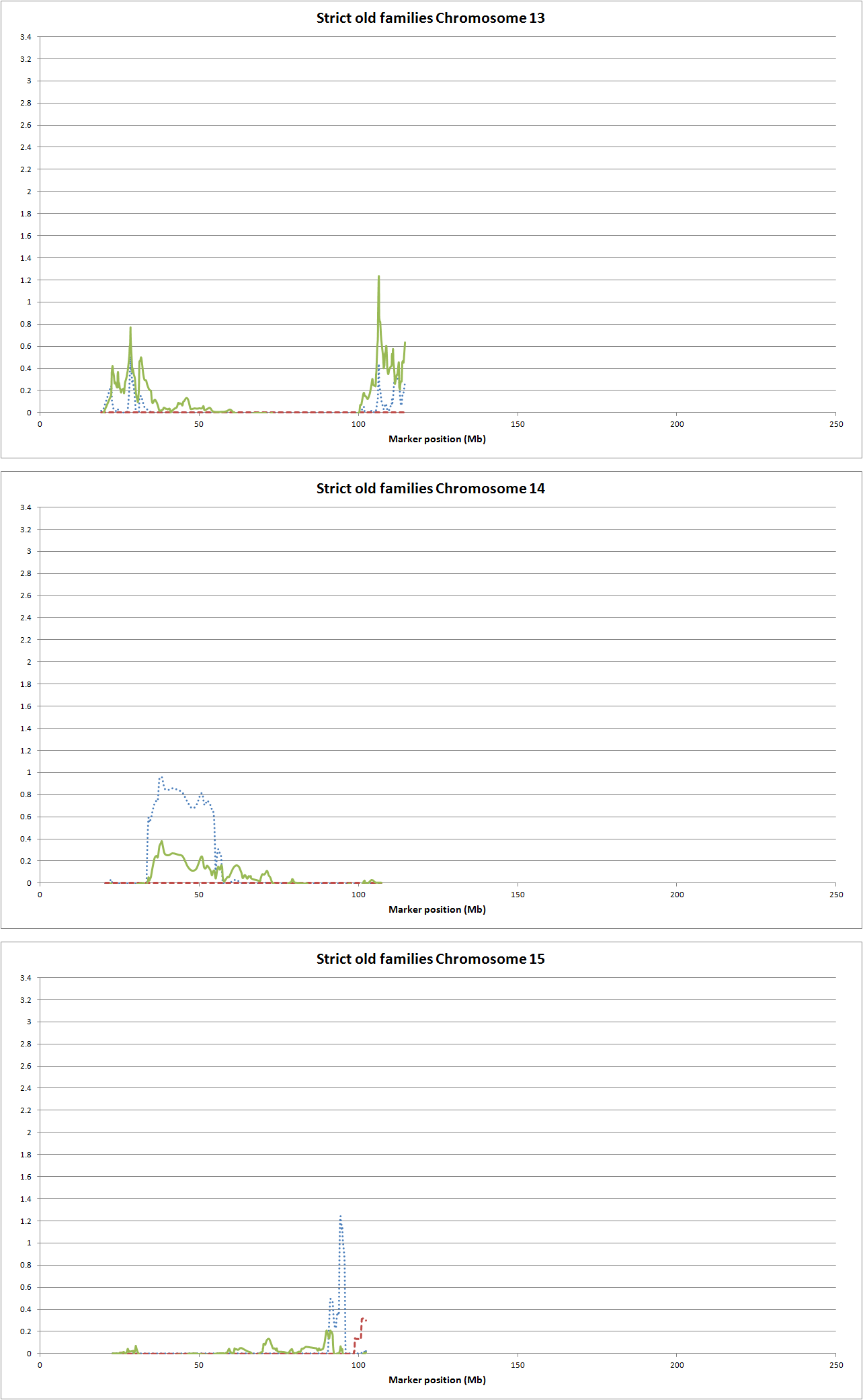


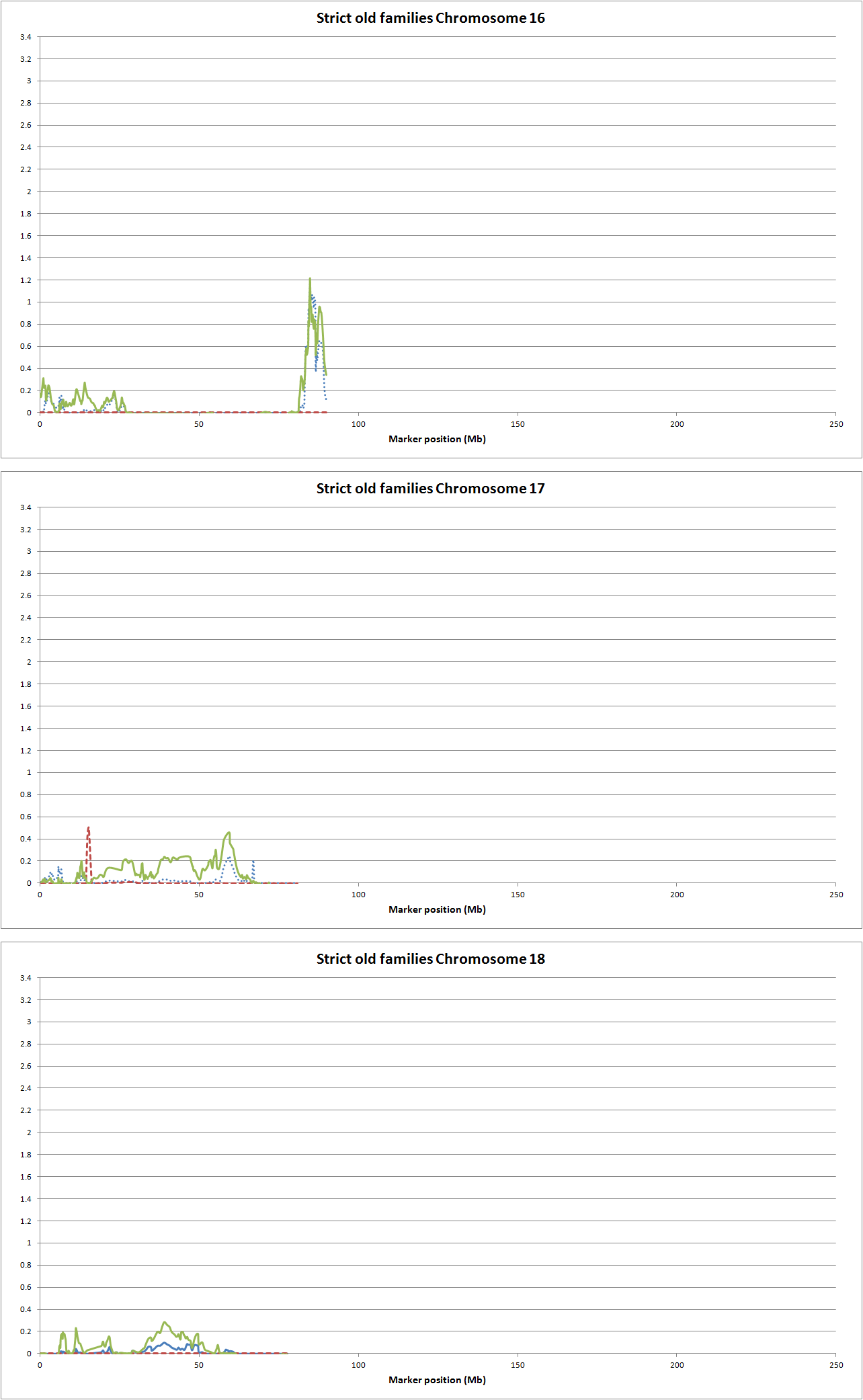


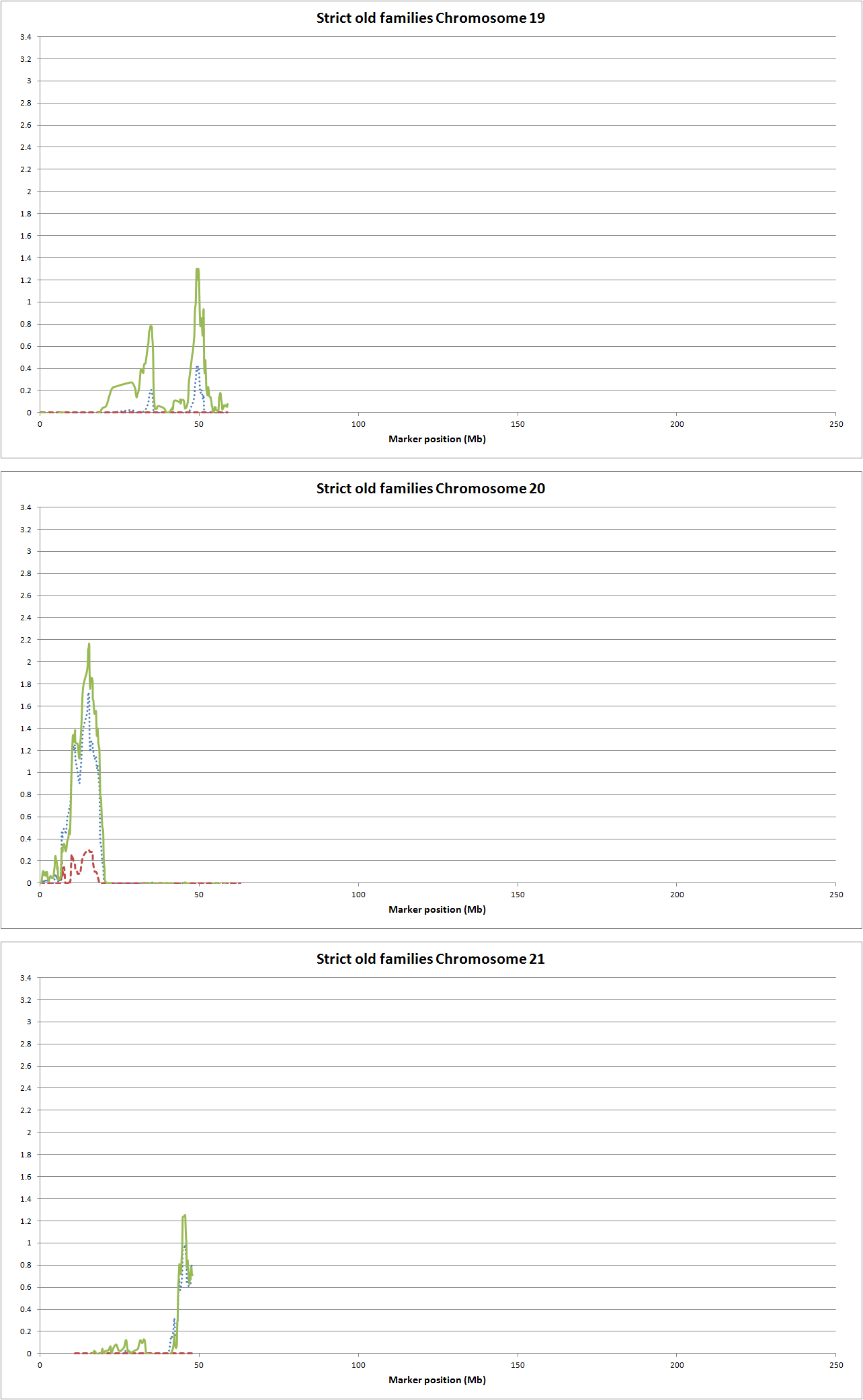


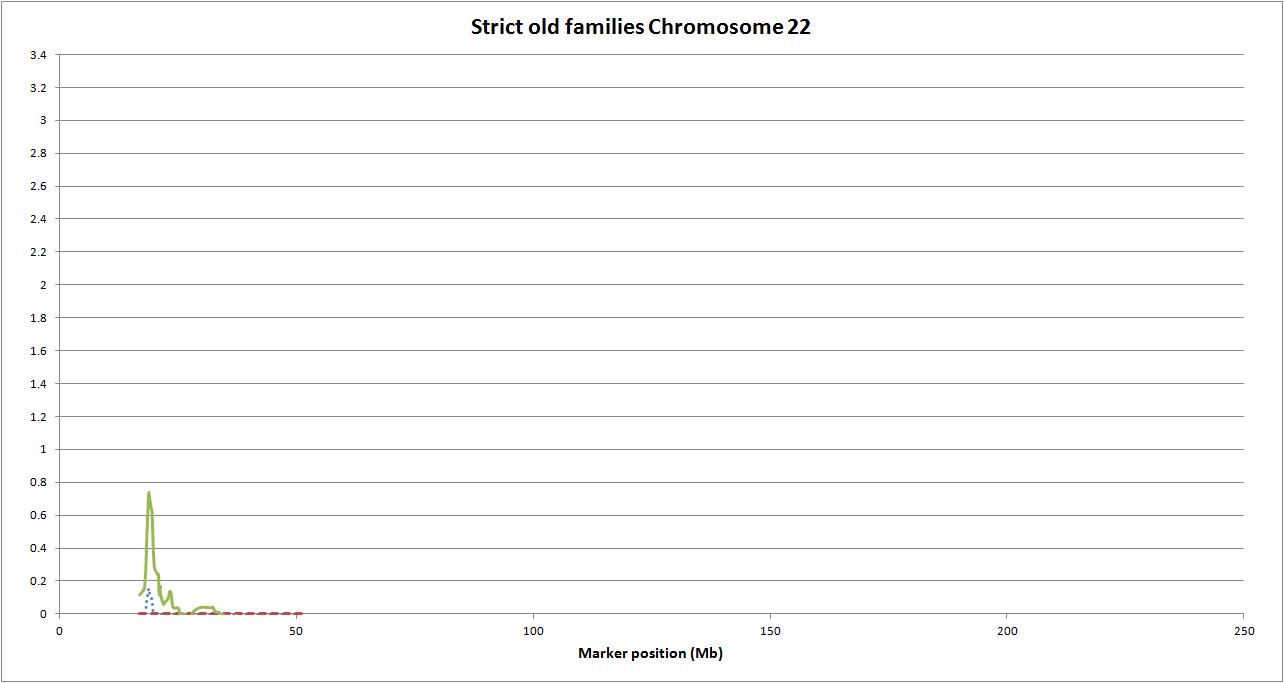

Supplement: Figure S7 — Linkage in the “old” families ______ HLOD dominant; ------- HLOD recessive; —— ZLRLOD [file mgg30002-0007-sd7.doc]

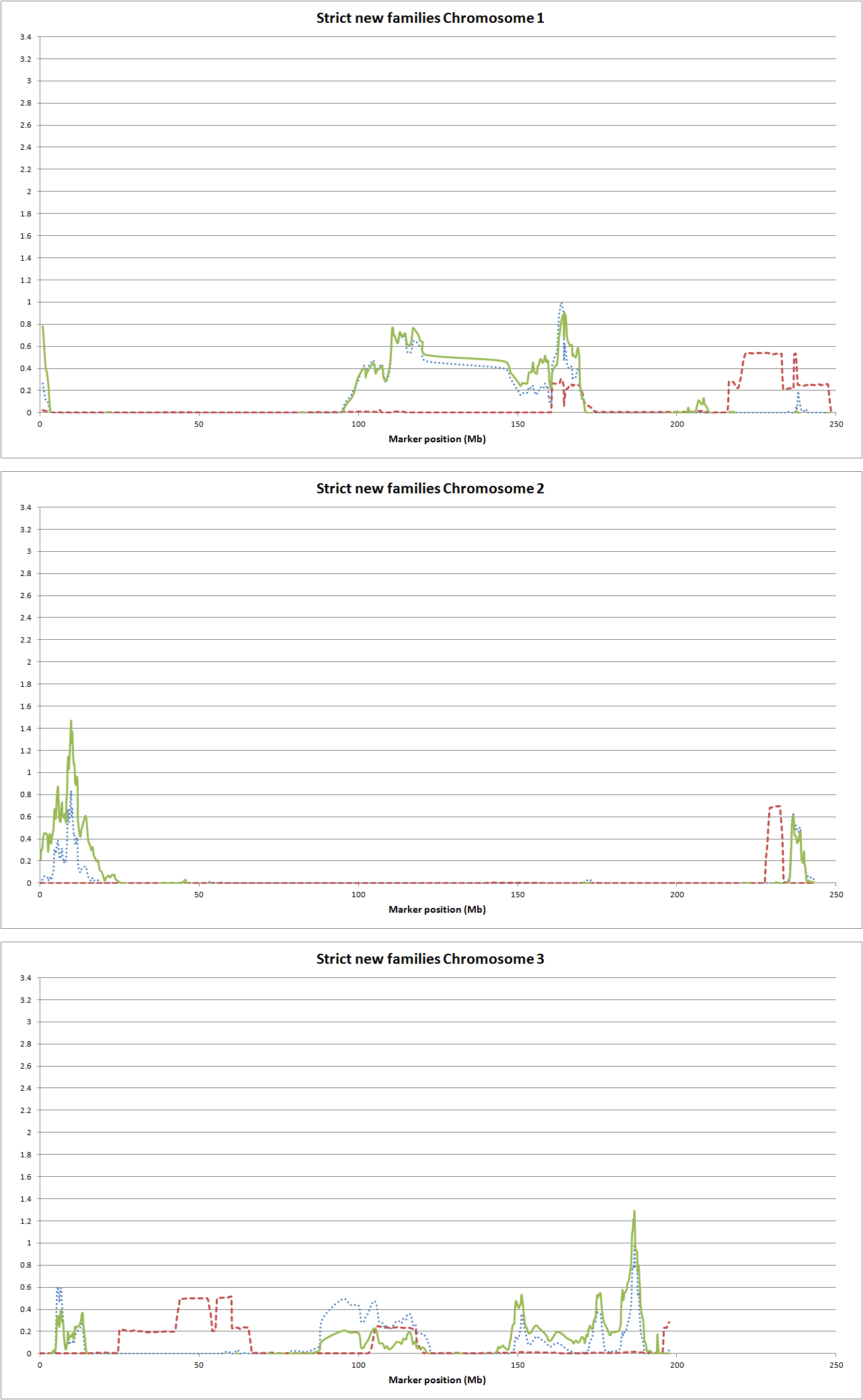


**Supplementary Figure S8.** Linkage in the ‘new’ families

······ HLOD dominant; ----- HLOD recessive; ~~-----~~ ZLRLOD.


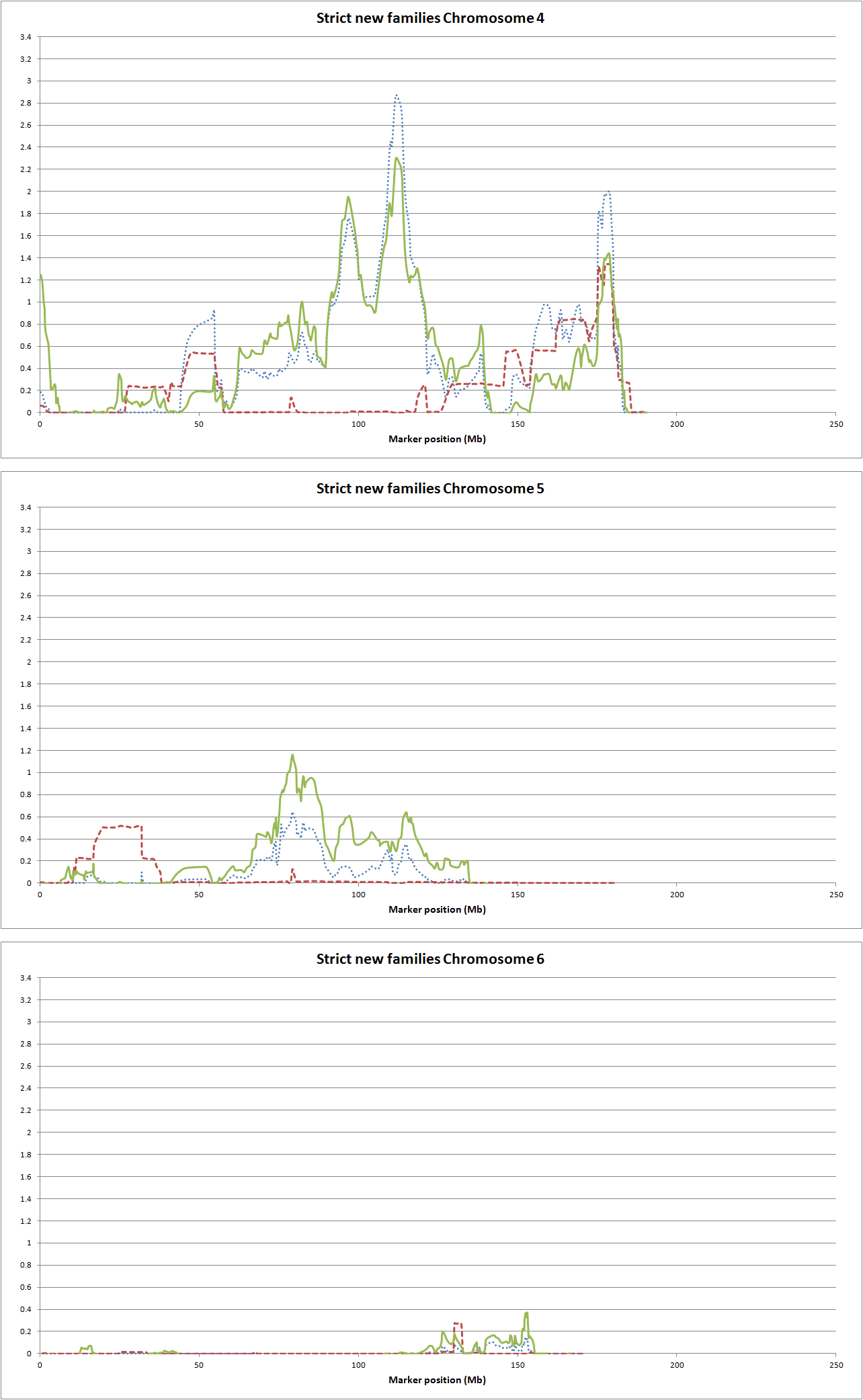


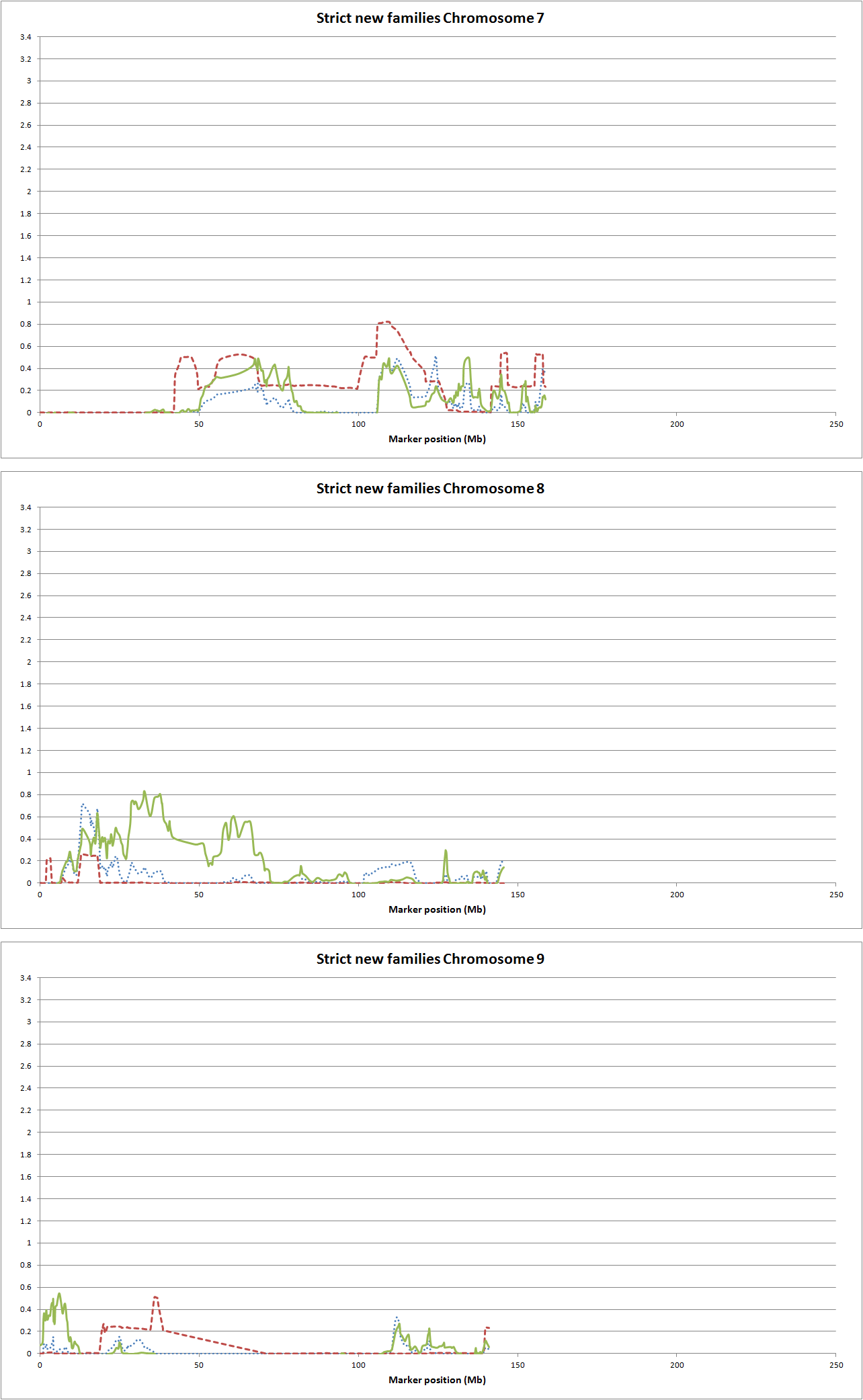


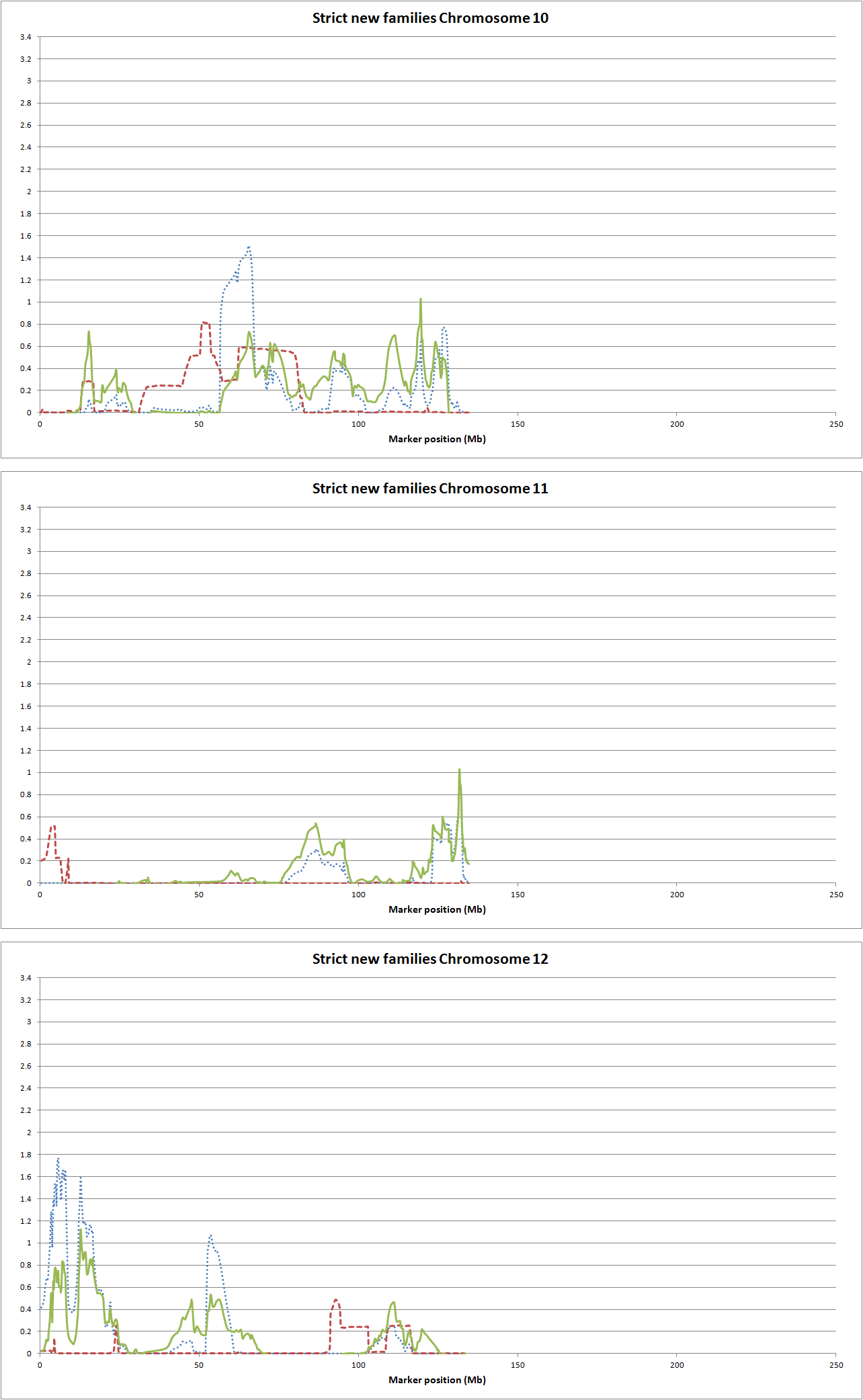


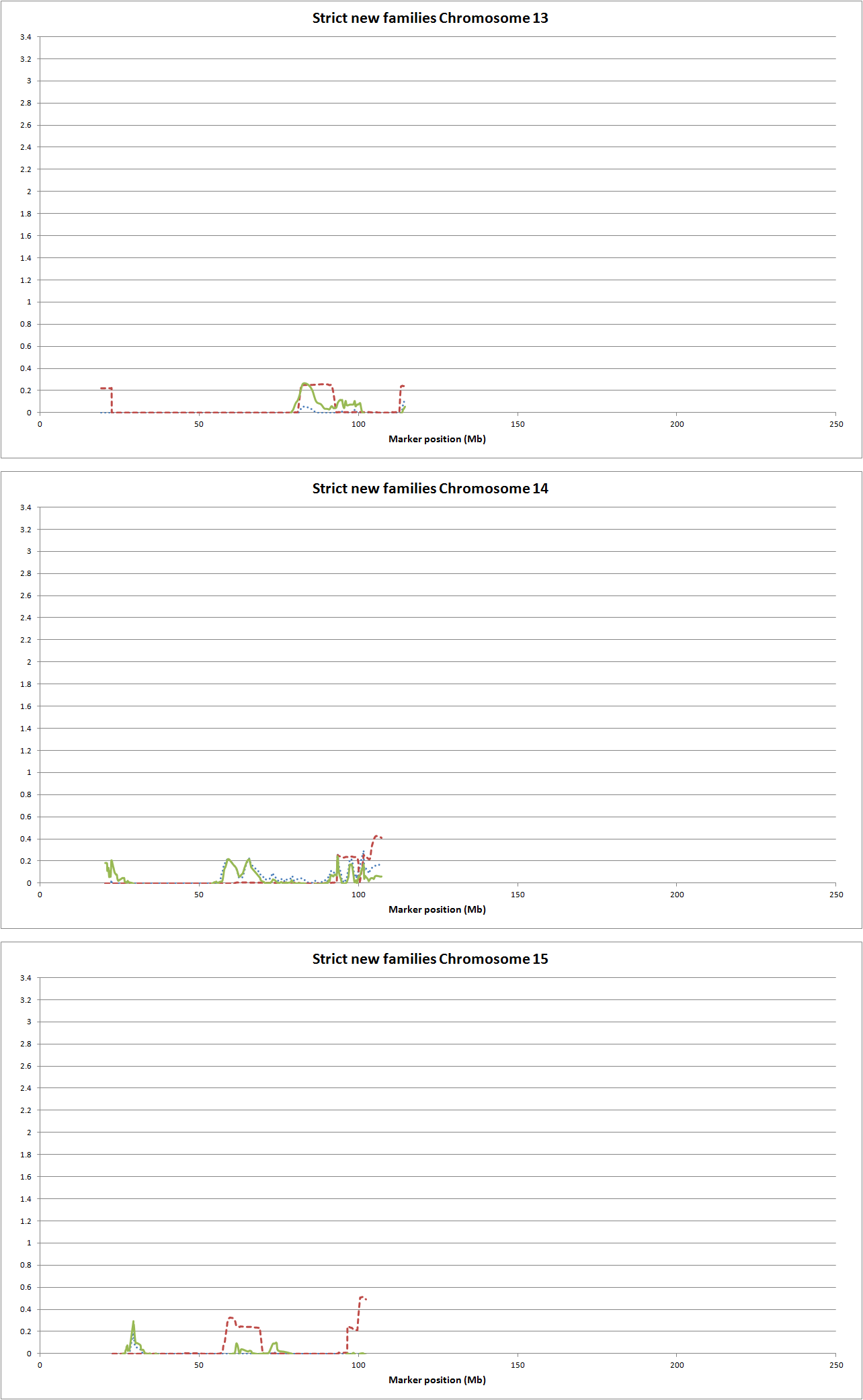


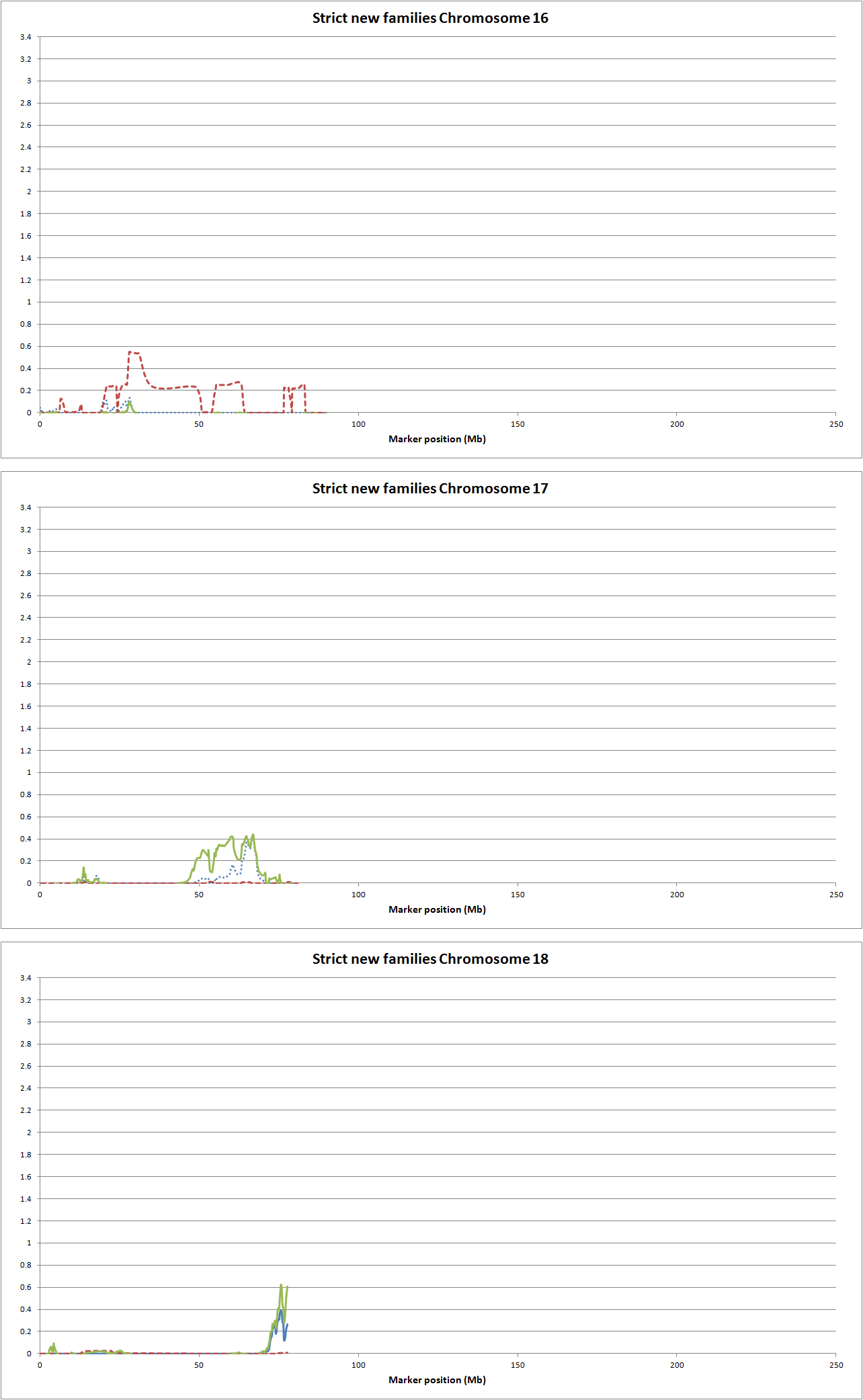


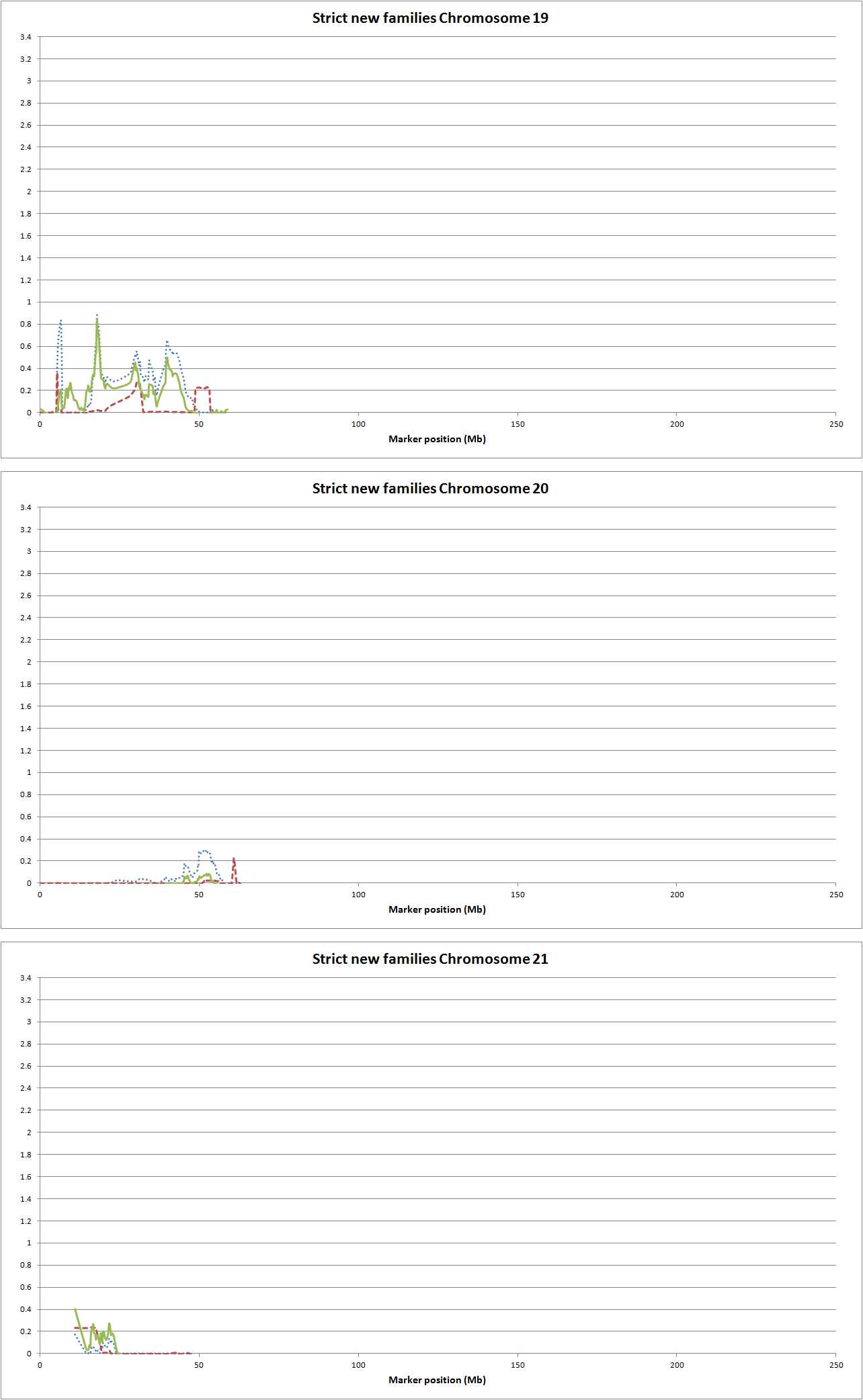


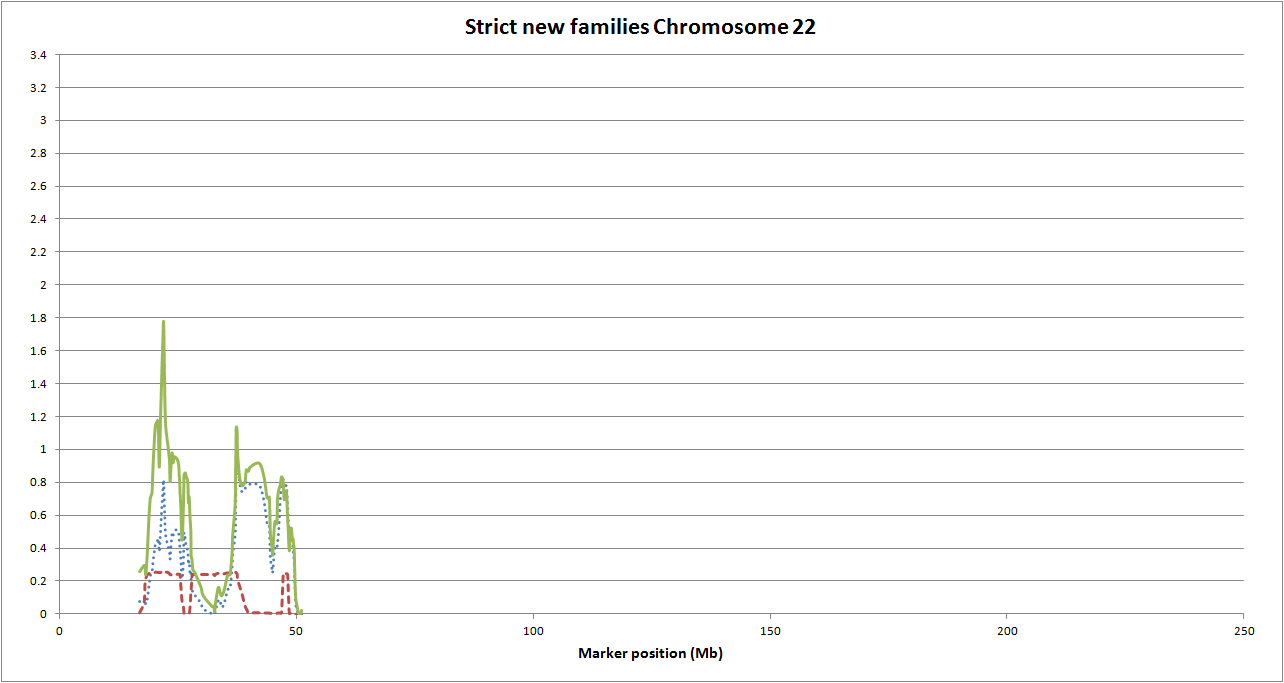

Supplement: Figure S8 — Linkage in the “new” families ______ HLOD dominant; ------- HLOD recessive; —— ZLRLOD. [file mgg30002-0007-sd8.doc]
