# Supplementary material for: A new genome scan for primary nonsyndromic vesicoureteric reflux emphasizes high genetic heterogeneity and shows linkage and association with various genes already implicated in urinary tract development
Source: Mol Genet Genomic Med. 2013 Jul 7;2(1):7–29. doi: 10.1002/mgg3.22 (PMC3907909; doi:10.1002/mgg3.22)
Supplement: Figure S3 — HLOD analysis with a dominant model of inheritance for the original families (top) the new and all families together. [file mgg30002-0007-sd3.doc]

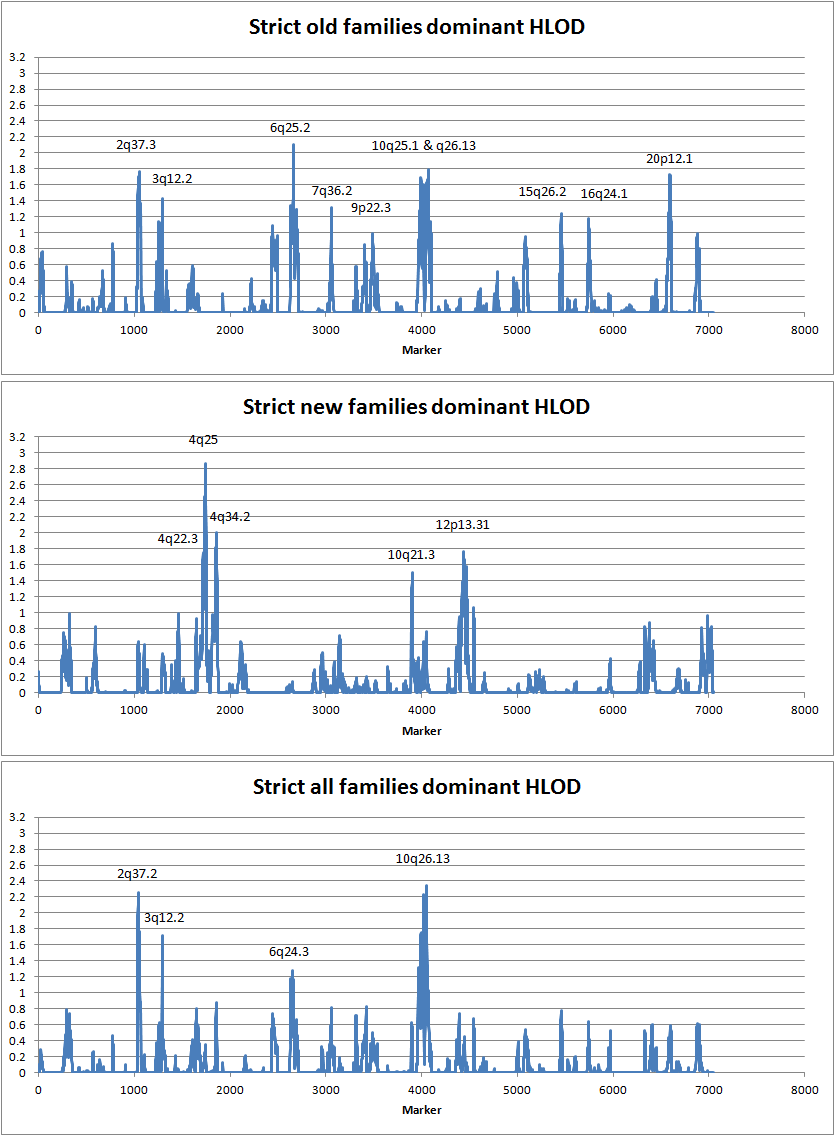


**Supplementary Figure S3**. HLOD analysis with a dominant model of inheritance for the original families (top) the new, and all families together.
