# Supplementary material for: A new genome scan for primary nonsyndromic vesicoureteric reflux emphasizes high genetic heterogeneity and shows linkage and association with various genes already implicated in urinary tract development
Source: Mol Genet Genomic Med. 2013 Jul 7;2(1):7–29. doi: 10.1002/mgg3.22 (PMC3907909; doi:10.1002/mgg3.22)
Supplement: Supplementary file 9 [file mgg30002-0007-sd9.doc]

**Supplementary Table S1. Numbers of samples used for ‘strict’ linkage.**

|  | Number of Families | Number of Samples | Number of affected | Number of Males | Affected Males | Number of Females | Affected Females |
| --- | --- | --- | --- | --- | --- | --- | --- |
| Old | 118 | 513 | 281 | 233 | 116 | 280 | 165 |
| New | 81 | 336 | 186 | 152 | 76 | 184 | 110 |
| Total | 199 | 849 | 467 | 385 | 192 | 464 | 275 |
